# DFT Surface Infers Ten-Vertex Cationic Carboranes from the Corresponding Neutral *closo* Ten-Vertex Family: The Computed Background Confirming Their Experimental Availability

**DOI:** 10.3390/molecules28083645

**Published:** 2023-04-21

**Authors:** Michael L. McKee, Jan Vrána, Josef Holub, Jindřich Fanfrlík, Drahomír Hnyk

**Affiliations:** 1 Department of Chemistry and Biochemistry, Auburn University, Auburn, AL 36849, USA; mckeeml@auburn.edu; 2 Faculty of Chemical Technology, University of Pardubice, CZ-532 10 Pardubice, Czech Republic; jan.vrana@upce.cz; 3 Institute of Inorganic Chemistry of the Czech Academy of Sciences, CZ-250 68 Husinec-Řež, Czech Republic; holub@iic.cas.cz; 4 Institute of Organic Chemistry and Biochemistry of the Czech Academy of Sciences, CZ-166 10 Praha 6, Czech Republic; jindrich.fanfrlik@uochb.cas.cz

**Keywords:** carboranes, N-heterocyclic carbenes, cations, DFT, reaction pathways

## Abstract

Modern computational protocols based on the density functional theory (DFT) infer that polyhedral *closo* ten-vertex carboranes are key starting stationary states in obtaining ten-vertex cationic carboranes. The rearrangement of the bicapped square polyhedra into decaborane-like shapes with open hexagons in boat conformations is caused by attacks of N-heterocyclic carbenes (NHCs) on the *closo* motifs. Single-point computations on the stationary points found during computational examinations of the reaction pathways have clearly shown that taking the “experimental” NHCs into account requires the use of dispersion correction. Further examination has revealed that for the purposes of the description of reaction pathways in their entirety, i.e., together with all transition states and intermediates, a simplified model of NHCs is sufficient. Many of such transition states resemble in their shapes those that dictate *Z*-rearrangement among various isomers of *closo* ten-vertex carboranes. Computational results are in very good agreement with the experimental findings obtained earlier.

## 1. Introduction

The exclusive chemistry of polyhedral carborane clusters is a result of the presence of delocalized electron-deficient bonding [1,2] based on the formation of multi-center–two-electron (mc-2e) bonds, which do not exist in classical organic chemistry, characterized by classical two-center–two-electron (2c-2e) bonds. When two carbons in a carborane cluster form the nearest-neighbor separation, such bonding is also of the 2c-2e nature [3]. The BH vertices in the carboranes are assembled in trigonal faces, which enables the creation of three-dimensional shapes, with octahedron [4], icosahedron, and bicapped square antiprism [2] being among the most prevalent, and are related to *closo* systems [1,2]. The preparation and subsequent reactivity of these clusters has been extensively studied by experiments [1,2].

In contrast to well-understood reaction mechanisms in organic chemistry, those in boron cluster chemistry can be very complex because there are very small energy differences between many possible intermediates and transition states. On that basis, the reaction of boron hydrides may involve many competing pathways [5]. Consequently, relatively little progress has been made so far in the understanding of the reaction mechanisms of boron hydrides and carboranes of various molecular shapes [6,7,8]. Apart from the reaction of the icosahedral carboranes [1,2], a few conversions of ten-vertex *closo* carboranes have already emerged.

**Scheme 1:**
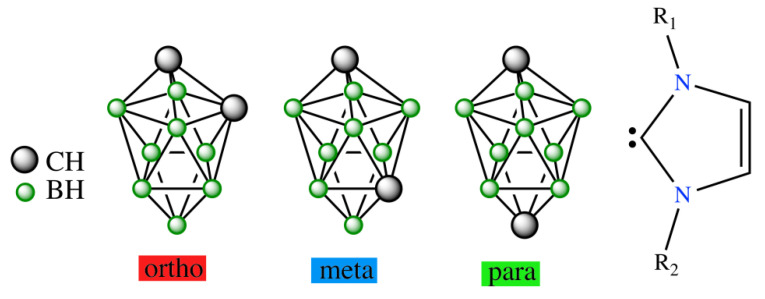
Molecular diagrams of *closo*-1,N-C_2_B_8_H_10_ (red: N = 2, blue: N = 6, green: N = 10) and N-heterocyclic carbenes.

The *closo*-C_2_B_8_H_10_ molecular shape adopting a bicapped square antiprism (Scheme 1) exists in seven positional isomers, only three of which (1,2-, 1,6-, and 1,10-isomers) are known experimentally [2]. The most stable one is the 1,10-isomer and the least stable one is the 2,3-isomer [9]. The mutual isomerization of these carboranes has recently been interpreted as a result of *Z*-rearrangement, which is characterized by a transition state of the decaborane molecular shape between various pairs of these seven isomers. Therefore, upon reaction with hydroxides and amines 1,2-isomer is converted into open *arachno* systems: see [10,11], respectively. These reaction pathways have recently been searched computationally [12]; these attempts have shown that the products are negatively charged or neutral. The same applies to the reaction of this isomer with “wet” fluoride [13]. Such charge conditions are very common in boron cluster chemistry. However, when three isomers of *closo*-1,N-C_2_B_8_H_10_ (N = 2,6,10) were allowed to react with N-heterocyclic carbenes (NHCs, Scheme 1), the cationic arrangements of decaborane shapes were obtained by HCl acidification of the neutral species of the decaborane shapes [14]. Note that such shapes were already spotted when these *closo* structural motifs originated by click chemistry [15], also including the aforementioned *Z*-rearrangement [16]. The existence of cationic ten-vertex carboranes, which retain the bicapped square antiprismatic arrangement only in the case of *closo*-1,10-C_2_B_8_H_10_, prompted us to search the corresponding reaction pathways of their formation computationally by employing modern computational protocols.

## 2. Results and Discussion

### 2.1. The Reactions of closo-1,2-C_2_B_8_H_10_ (o) with NHC

**Figure 1:**
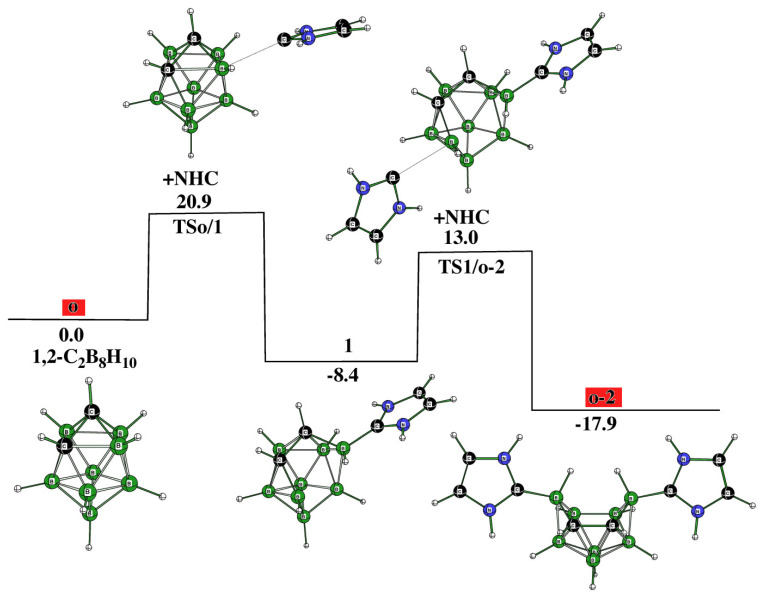
Relative free energies (kcal.mol^−1^) of the individual stationary points on the potential energy surface (PES) of the formation of **o-2** (see Table 1 and Table S1 and the Section 3 for details).

The reaction starts with the attack of a lone electron pair of a NHC on one of the most positive boron atom pairs within the cage, i.e., on B(3) [10,11,12], and following this proceeds very smoothly; **o-2**, conforming to the decaborane shape, is afforded (in line with Ref. [14]) via two transition states and one intermediate **1**, in which the opening of the square antiprismatic cage is marked: see Figure 1. The position of the C-C vector in **o-2** is consistent with that observed in the transition state of the isomerization of *closo*-1,2-C_2_B_8_H_10_ to itself due to *Z*-isomerization, with the latter being experimentally observed in the R-substituted derivatives of *closo*-1,2-C_2_B_8_H_10_ [16].

**Figure 2:**
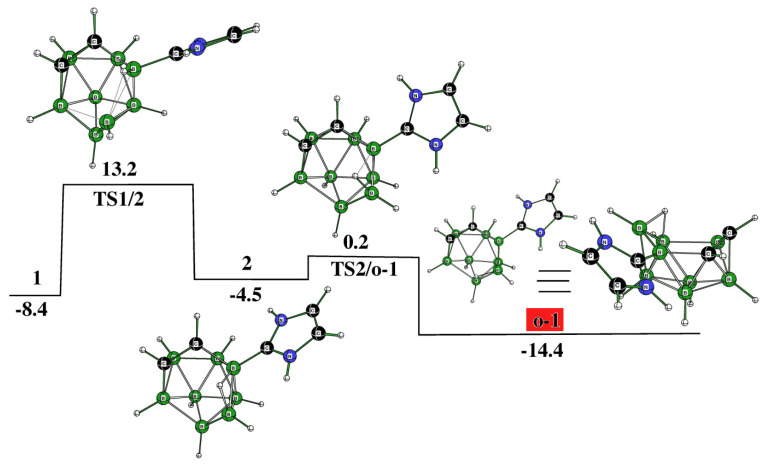
Relative free energies (kcal.mol^−1^) of the individual stationary points on the potential energy surface (PES) of the formation of **o-1** (see Table 1 and Table S1 and Section 3 for details).

However, **1** appears to be an intermediate that brings about the formation of not only **o-2** but also other experimentally available decaborane-like shaped **o-1**, which can originate, as seen, from the kinetics depicted in Figure 2. Like **o-2**, **o-1** also occurs through two TSs and one intermediate, i.e., **2** in this case. This reaction path shows that the cage deformation, demonstrated by inspecting TS1/2, is achieved without using another NHC, with the latter being necessary for obtaining **o-2**. Both **o-1** and **o-2** have space for accommodating an extra hydrogen bridge to arrive at the cationic motifs **o-1a** and **o-2a**. The molecular shape of **o-1** is identical to the shape of the TS, through which the isomerization of 1,2- to 2,6-C_2_B_8_H_10_ occurs due to *Z*-rearrangement [16].

**Figure 3:**
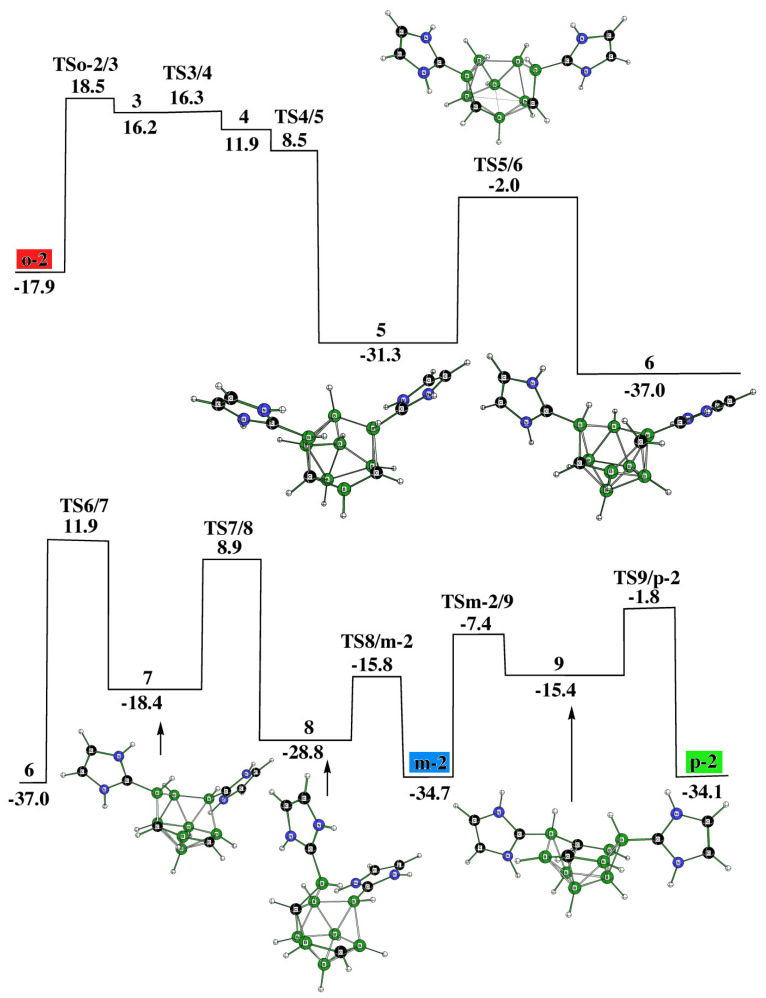
Relative free energies (kcal.mol^−1^) of the individual stationary points on the potential energy surface (PES) of the formation of **m-2** and **p-2** from **o-2**. Only a few TSs and intermediates are shown for simplicity (see Table 1 and Table S1 and Section 3 for details).

It has already been mentioned that the individual species within the *closo*-C_2_B_8_H_10_ family are prone to mutual isomerization. This process was also detected experimentally among the 1,2-(**o**), 1,6-(**m**), and 1,10-(**p**) isomers and was proposed to be explained in terms of *Z*-isomerization [16], although the previously suggested diamond-square-diamond (DSD) mechanism cannot be completely ruled out [18]. Guided by these results, we also examined whether such a linkage could exist among the corresponding C_2_B_8_H_10_ architectures of the decaborane shape, i.e., among **o-2** (see Figure 1), **m-2** (see below), and **p-2** (see below). Indeed, such isomerizations have been computed to be feasible, as depicted in Figure 3. Seemingly, this overall process is intrinsically more demanding and occurs through a cascade of nine TSs and seven intermediates until the conversion from **o-2** to **p-2**, such as from **o** to **p**, has been accomplished: see below for the molecular diagrams of **m-2** and **p-2**.

### 2.2. The Reactions of closo-1,6-C_2_B_8_H_10_ (m) with NHC

**Figure 4:**
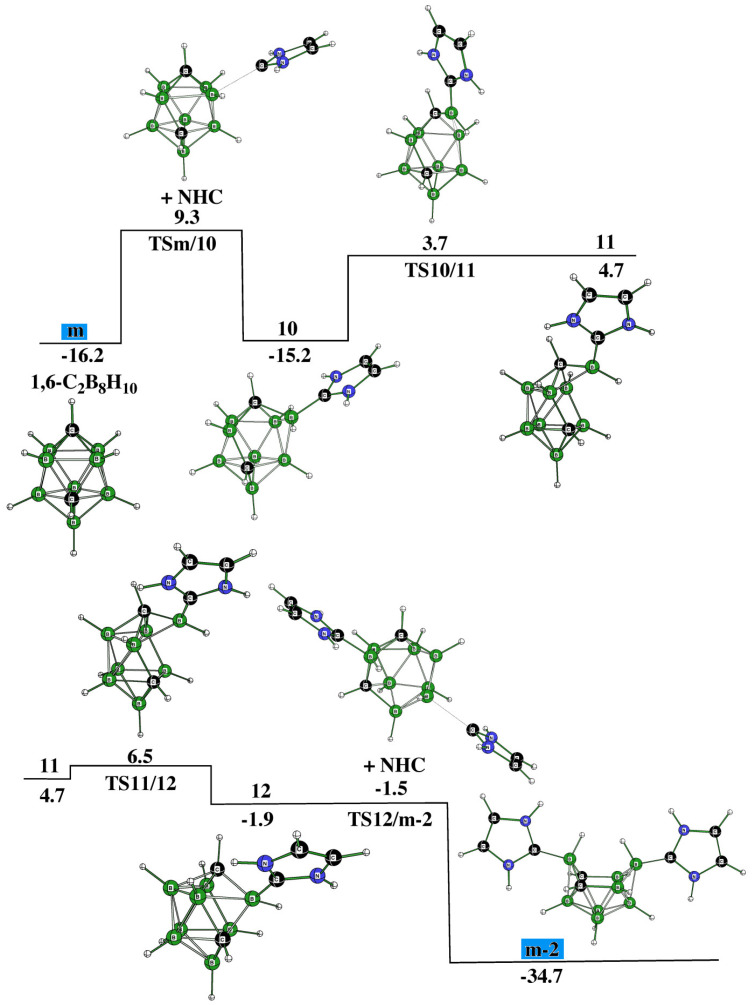
Relative free energies (kcal.mol^−1^) of the individual stationary points on the potential energy surface (PES) of the formation of **m-2** from **m** (see Table 1 and Table S1 and Section 3 for details).

Under favorable circumstances, **m-2** can directly originate from **o-2**; nevertheless, we tried to model the experimentally proved reaction [14] that transforms 1,6-C_2_B_8_H_10_, **m**, to experimentally detected **m-2** [14]. **TSm/10** is a result of the NHC attack, see Figure 4, on the positive B(3) atom, which is in analogy with the formation of **o-2**. However, by continuing from this stationary point, the reaction pathway becomes quite complex as proven by locating many more TSs and intermediates on this surface (Figure 4).

### 2.3. The Reactions of closo-1,10-C_2_B_8_H_10_ (p) with NHC

As in the **o**-to-**o-2** transformation, two TSs and one intermediate (i.e., **13**) are needed to obtain **p-2** from **p**. In other words, such a process is relatively easy to accomplish. As in the preceding two *closo* to electron-count-obeying *hypercloso*-like transformations (note that the NHC acts as an electron donor to the electron-deficient *closo* shape, with the latter appearing to be a slight electron acceptor experimentally [19]), it is the positively charged B(3)H vertex that accepts a lone pair of the C atoms within the carbene molecule. Intermediate **13** demonstrates the initiation of such a cage-opening process.

**Figure 5:**
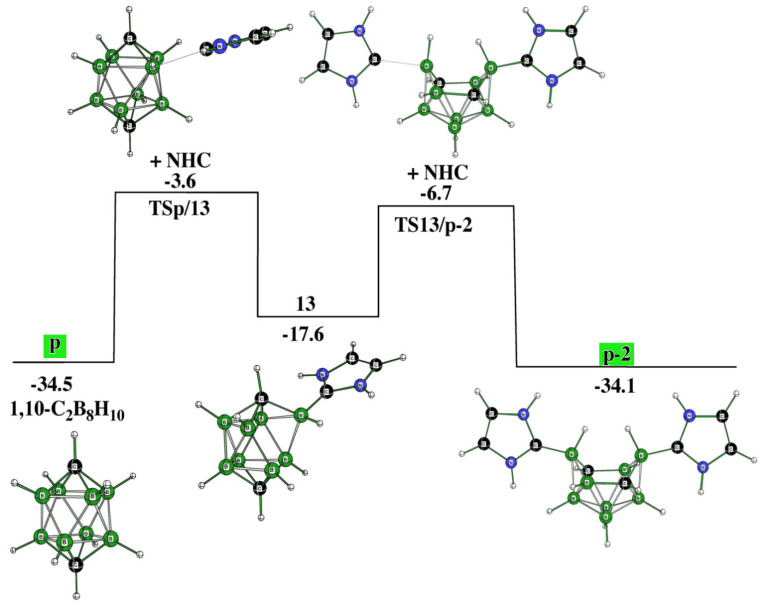
Relative free energies (kcal.mol^−1^) of the individual stationary points on the potential energy surface (PES) of the formation of **p-2** from **p** (see Table 1 and Table S1 and the Section 3 for details).

Interestingly, a feature of the experimentally known **p-2** bears a strong resemblance to the transition state of the 2,7- to 1,10-C_2_B_8_H_10_ isomerization under the recently coined *Z*-rearrangement [16], see Figure 5.

**Figure 6:**
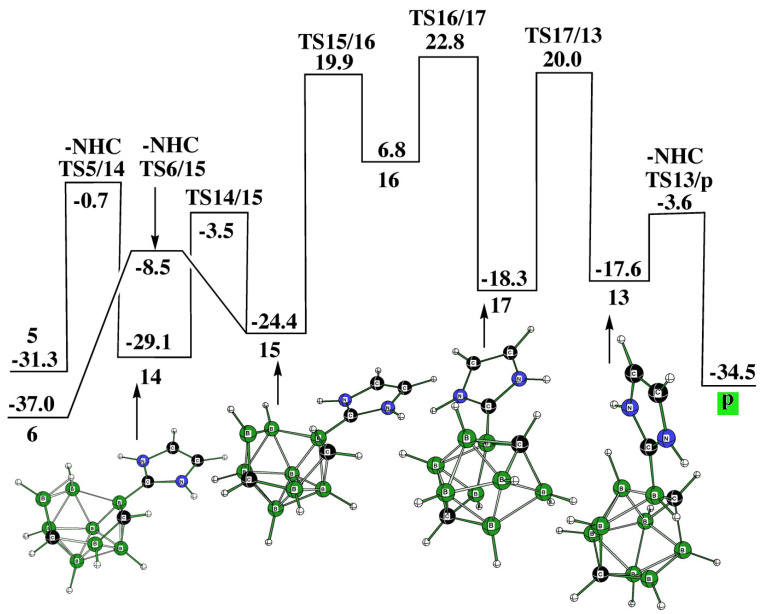
Relative free energies (kcal.mol^−1^) of the individual stationary points on the potential energy surface (PES) of the reformation of **p** from both **o-2** and **m-2** (see Table 1 and Table S1 and Section 3 for details).

Synthetic routes [16] have also revealed the cage closure from both **o-2** and **m-2** to obtain **p**, with the latter being the starting stationary point to obtain **p-2** as described in [16] and computed in this work. In other words, the connection between **o-2**, **m-2,** and **p-2** examined experimentally has been confirmed computationally. Relatively stable intermediates **5** and **6**, located also in the pathway from **o-2** to **p-2** (see Figure 3), open the cage closure with the resulting **p** (Figure 6). This complex procedure is surmounted by six TSs, which proceed through four intermediates. The fifth intermediate, **13**, responsible for the elimination of the second NHC, links this pathway together with that describing the cage opening **p** to **p-2** (Figure 3). In essence, Figure 3, Figure 5 and Figure 6 illustrate the possible reversibility of the **p**-to-**p-2** and **p-2**-to-**p** processes.

**Figure 7:**
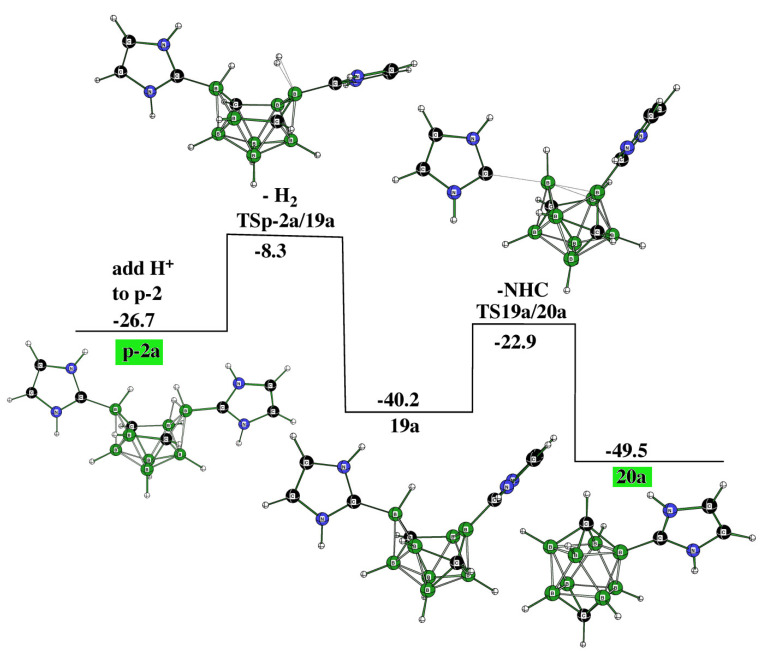
Relative free energies (kcal.mol^−1^) of the individual stationary points on the potential energy surface (PES) of the entire protonation of **“p-2a”** (see Table 1 and Table S1 and Section 3 for details).

In essence, both **o-2** and **m-2** are very easily protonated (with HCl) to provide the *cationic* structures **o-2a** and **m-2a**. These processes are exothermic, e.g., the energy gain of the first process is computed to be 15.3 kcal.mol^−1^. In contrast, the protonation of **p-2** is mechanistically more complex: **“p-2a”** (not the final product of the protonization) with the same molecular shape as **o-2a** and **m-2a** is not the final product of this acidification. The presence of the extra hydrogen bridge in the **“p-2a”** arrangement results in the elimination of one NHC; through two cationic TSs and one intermediate, the cationic product **20a** is then formed as the final product, see Figure 7.

**Figure 8:**
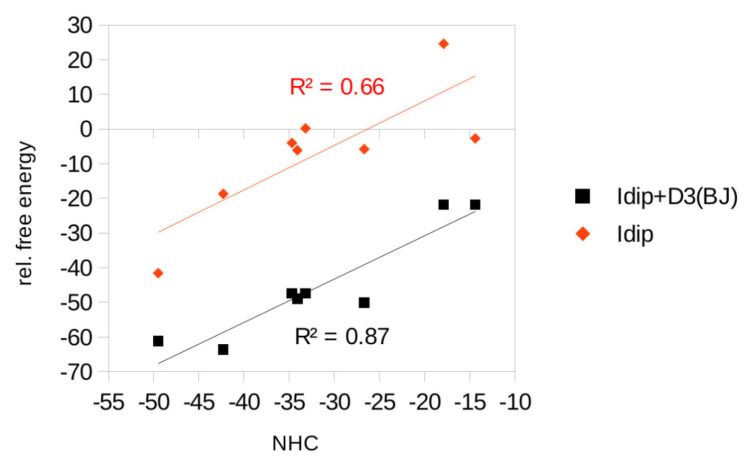
The correlation of the relative free energies of the existing species given in Table 1, optimized both with dispersion correction (black, Idip + D3(BJ)) and without it (red, Idip), with those related to stationary points in which Idip is simplified with NHC.

Table 1 shows that the energetic balance in both columns exhibits a very reasonable correlation (see also Figure 8). In other words, the necessity of including the dispersion correction when recomputing the energetic criteria with the carbene bearing 1,3-*i*Pr2-C_6_H_3_, i.e., with Idip (b) with respect to (a), indicates agreement with the fact that only the electron sextet on the carbene moiety justifies the modeling of the Idip with simpler NHCs in the search of the reaction pathways, to reduce the problem to more manageable dimensions. Note that for the cationic species, the necessity of considering dispersion energy is less crucial.

### 2.4. Further Possible Ten-Vertex Carbocations

**Figure 9:**
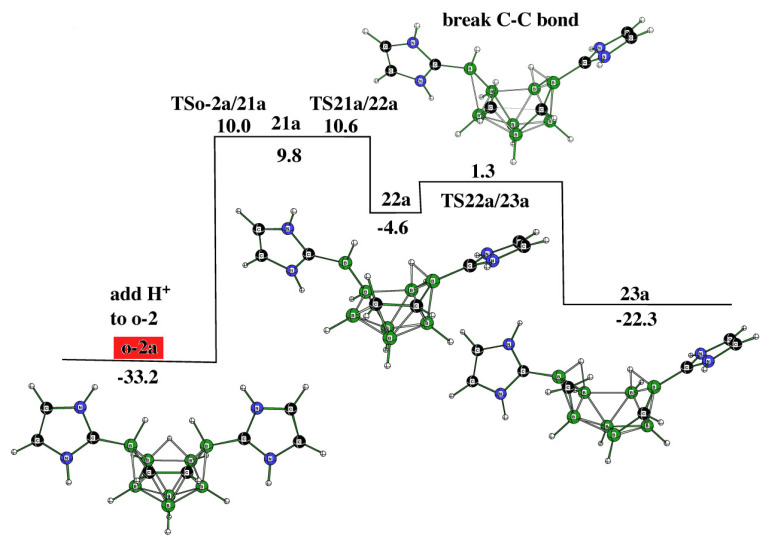
Relative free energies (kcal.mol^−1^) of the individual stationary points on the potential energy surface (PES) of the formation of the cation, **23a**, through another cation, **22a** (see Table 1 and Table S1 and Section 3 for details).

**o-2** is the most stable cation among those derived earlier. The very stable C-C bond in the carborane cage might be one of the driving forces to support this. The possible cleavage of this bond may generate another cation, **23a**, which resembles the TS responsible for the isomerization of the 2,3-C_2_B_8_H_10_ to the 1,6-isomer (see Figure 9). The driving force of this pathway is the formation of CH_2_, which is, like the C-C vector, a stable moiety. Moreover, such a pathway clearly goes through the stable but not isolable cation **22a**, resembling **o-2a** from which it differs in the position of the hydrogen bridge and the mutual position of the NHC-type pentagons.

**Figure 10:**
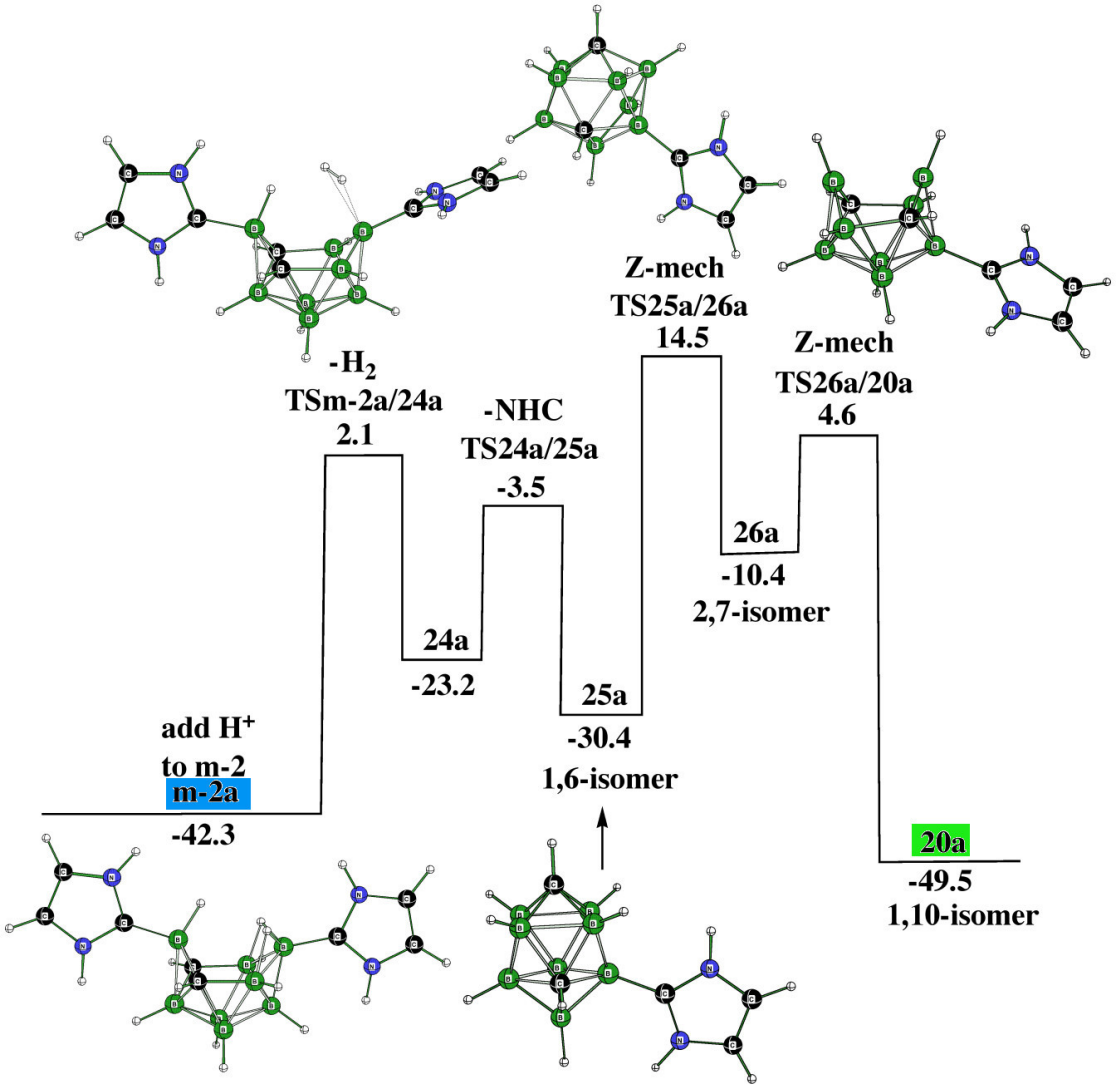
Relative free energies (kcal.mol^−1^) of the individual stationary points on the potential energy surface (PES) of the transformation of **m-2a** through the cationic **25a** based on the 1,6-isomer and two *Z*-rearrangements to the final **20a** (see Table 1 and Table S1 and Section 3 for details).

**m-2a** is more stable than **“p-2a”**, on the basis of which it can serve as the more important intermediate in further pathway linkages. Figure 10 illustrates how this cation, by losing two hydrogens, can also be converted to **20a**, the latter being located also in the pathway depicted in Figure 7. Such a conversion occurs via the cation **25a**, which appears to be in analogy with **20a**. Interestingly, such a process is accomplished through two *Z*-rearrangements, as revealed by the last two TSs [16].

## 3. Methods

Free energies of all the stationary points appeared in the reactions of *closo*-1,2-, 1,6-, and 1,10-C_2_B_8_H_10_ with a NHC in which both bulky Rs, represented in Ref. [14] with 1,3-*i*Pr_2_-C_6_H_3_ and abbreviated as Idip, were replaced with hydrogens during the pathway examinations (NHC in further notations below), and the SMD(DiethylEther)/B3LYP/6-311+G(2d,p)//B3LYP/6-31G(d) computational protocol was used. Seemingly, DiethylEther turned out to be a good approximation for CPME (not in the library of G16), in the latter protonizations that occurred [14]. The reactants and products were connected from the corresponding transition states in terms of computing the intrinsic reaction coordinates (IRC). However, such computations were not carried out in cases where the reactant and product were obvious from inspecting the corresponding transition vector, with the latter being revealed from the second derivative analysis. Such physically correct TSs are recognized as being responsible, e.g., for a simple addition of a NHC or a hydrogen movement. Due to the presence of benzene rings and *i*Pr groups in Idip, it has many non-bonded distances that are below the sum of the van der Waals radii. Therefore, the importance of the inclusion of a dispersion correction was obvious. This was resolved by adding the D3(BJ) correction [17,20] to the B3LYP/6-311+G(2d,p) model chemistry in the single-point computations of all experimentally detectable species, as shown in Table 1 when Idip was used in the computations. The “a” indicates a cation, the integers are unobserved intermediates, and TSs (“reactant”/“products”) refers to transition states; these notations are common for the reaction pathways below. All the computations were performed using Gaussian16, in which the above model chemistries and basis sets are incorporated [21]. *closo*-1,2-, 1,6-, and 1,10-C_2_B_8_H_10_ and the corresponding stationary points are marked in red trace, blue trace, and green trace, respectively.

## 4. Conclusions

The computational protocol based on the density functional theory computational tool has revealed the formation of various cationic polyhedral structures of *nido* and *closo* molecular shapes, as a result of the reaction of ten-vertex *closo* dicarboranes with a N-heterocyclic carbene modeled with a simple C_3_N_2_H_4_ pentagonal belt. Since previously obtained experimental ^11^B chemical shifts of the cations were very successfully reproduced by high-level computations using this simple carbene, this approximation is justified. Based on that, all previously experimentally observed cations were computationally processed in the single-point computations in the same manner as the syntheses reported in Ref. [14], i.e., with a N-heterocyclic carbene in which nitrogen-bonded hydrogens were replaced with 1,3-*i*Pr-C_6_H_3_. Seemingly, the corresponding computed energetic balance was in line with that in which the simple carbene was considered only if the correction for dispersion energy was used in these more demanding computational efforts. Further computational experiments open the possibility of the existence of other cationic isomers of such nature, which offers experimenters further challenges.

## Figures and Tables

**Table 1:** Free energies (kcal.mol^−1^) of the experimentally inferred products (characterized by ab initio/GIAO/NMR and X-ray diffraction structural tools [14]) relative to 1,2-C_2_B_8_H_10_ (o) + one or two NHC(s).

Reactant to Product	B3LYP ^a^	B3LYP ^b^
**o** to **o-1**	−14.4	−21.8 (−2.7)
**o** to **o-2**	−17.9	−21.8 (24.6) ^c^
**m** to **m-2**	−34.7	−47.4 (−4.0) ^c^
**p** to **p-2**	−34.1	−49.0 (−6.1)
**o-2** to **o-2a**	−33.2	−47.4 (0.2)
**m-2** to **m-2a**	−42.3	−63.6 (−18.7)
**p-2** to **p-2a**	−26.7	−50.1 (−5.8)
**p-2a** to **20a**	−49.5	−61.2 (−41.6)

^a^ SMD(DiethylEther)/B3LYP/6-311+G(2d,p)//B3LYP/6-31G(d), with NHC being employed for searching the reaction pathways as well as in single-point computations. ^b^ SMD(DiethylEther)/B3LYP/6-311+G(2d,p)+D3(BJ)//B3LYP/6-31G(d): single-point computations of the stationary points were carried out with Idip under the same reaction pathways as in (a). The values in parentheses result from not considering dispersion correction by Grimme [17] (D3(BJ)) in the single-point computations. ^c^ Of the two isomers differing in the mutual positions of the carbene moieties, only that with the lower energy is tabulated.

## Data Availability

Data supporting reported results may be available on demand (to the corresponding author).

## Supplementary Materials

The following supporting information can be downloaded at: https://www.mdpi.com/article/10.3390/molecules28083645/s1, Table S1: Total Energies (hartrees) and Free Energies (kcal.mol^−1^) Relative to 2NHC+1,2-C_2_B_8_H_10_ or 2Idip+1,2-C_2_B_8_H_10_, Table S2: Cartesian Coordinates of Stationary Points listed in Table S1 Optimized at the B3LYP/6-31G(d) Level.
