# Supplementary material for: DFT Surface Infers Ten-Vertex Cationic Carboranes from the Corresponding Neutral closo Ten-Vertex Family: The Computed Background Confirming Their Experimental Availability
Source: Molecules. 2023 Apr 21;28(8):3645. doi: 10.3390/molecules28083645 (PMC10141709; doi:10.3390/molecules28083645)
Supplement: Supplementary file 1 [file molecules-28-03645-s001.zip › molecules-2338487-supplementary.pdf]

Supplementary Materials

DFT Surface Infers Ten-Vertex Cationic Carboranes from the Corresponding Neutral *Closo* Ten-Vertex Family: The Computed Background Confirming Their Experimental Availability

Table S1. Total Energies (hartrees) and Free Energies (kcal.mol^{-1}) Relative to 2NHC+1,2-$C_2B_8H_{10}$ or 2Idip+1,2-$C_2B_8H_{10}$

Notation	SMD(DiEthylEther)/ B3LYP/6-311+G(2d,p)// B3LYP/6-31G(d)[a,f]	ΔG(DEE,298K)[b]
h_2	-1.179382	
nhc	-226.266248	0.0
o	-281.182724	0.0
TSo/1	-507.432413	20.9
1	-507.484423	-8.4
TS1/o-2	-733.734716	13.0
o-2	-733.787922	-17.9
TS1/2	-507.447611	13.2
2	-507.477136	-4.5
TS2/o-1	-507.467559	0.2
o-1	-507.494329	-14.4
p	-281.241621	-34.5
TSp/13	-507.474530	-3.6
13	-507.498366	-17.6
TS13/p-2	-733.766674	-6.7
p-2	-733.814923	-34.1
TSo-2/3	-733.725576	18.5
3	-733.728037	16.2
TS3/4	-733.728110	16.3
4	-733.740811	11.9
TS4/5	-733.740909	8.5
5	-733.808048	-31.3
TS5/6	-733.758792	-2.0
6	-733.819073	-37.0
TS6/7	-733.735760	11.9
7	-733.786000	-18.4
TS7/8	-733.738867	8.9
8	-733.804284	-28.8
TS8/m-2	-733.781449	-15.8
TSm-2/9	-733.767404	-7.4
9	-733.779640	-15.4
TS9/p-2	-733.758536	-1.8
m	-281.209091	-16.2

TSm/10	-507.451673	9.3
10	-507.495484	-15.2
TS10/11	-507.463048	3.7
11	-507.460468	4.7
TS11/12	-507.458765	6.5
12	-507.472445	-1.9
TS12/m-2	-733.758184	-1.5
m-2	-733.814934	-34.7
TS5/14	-733.754639	-0.7
14	-507.517441	-29.1
TS14/15	-507.473069	-3.5
15	-507.509838	-24.4
TS6/15	-733.767806	-8.5
TS15/16	-507.436455	19.9
16	-507.459341	6.8
TS16/17	-507.431420	22.8
17	-507.498736	-18.3
TS17/13	-507.434923	20.0
p-2a	-734.253381	-26.7
TSp-2a19a	-734.219203	-8.3
19a	-733.073819	-40.2
TS19a/20a	-733.042901	-22.9
20a	-506.801367	-49.5
o-2a	-734.265808	-33.2
TSo-2a/21a	-734.191626	10.0
21a	-734.192156	9.8
TS21a/22a	-734.191438	10.6
22a	-734.216167	-4.6
TS22a/23a	-734.206516	1.3
	Exsisting entries below were made with SMD(DiEthylEther)/ B3LYP/6-311+G(2d,p)+D3(BJ)	
Idip	-1160.535292	0.0
o	-281.230118	0.0
Idip(o-1)	-1441.828914	-21.8
Idip (o-2)-a[d]	-2602.394971	-21.8
Idip (o-2)-b[d]	-2602.394966	-21.7
Idip (m-2)-a[e]	-2602.433192	-47.0
Idip (m-2)-b[e]	-2602.433390	-47.4
Idip (20a)	-1441.140689	-61.2
Idip (p-2)	-2602.435708	-49.0
Idip (o-2a)	-2602.887488	-47.3
Idip (m-2a)	-2602.909432	-63.6
Idip (p-2a)	-2602.886318	-50.1

(a) The calculation with the Idip ligands were done with the D3(BJ) correction.
(b) Zero-point correction, heat capacity correction, and entropy correction to 298K made with frequencies from the B3LYP/6-31G(d) level.
(c) Known species have the following designations:
o = 1,2-$C_2B_8H_{10}$
m = 1,6-$C_2B_8H_{10}$
p = 1,10-$C_2B_8H_{10}$

o-1 = $C_2B_8H_{10}$-NHC
o-2 = $C_2B_8H_{10}$-(NHC)$_2$
m-2 = $C_2B_8H_{10}$-(NHC)$_2$
p-2 = $C_2B_8H_{10}$-(NHC)$_2$
o-2a = $C_2B_8H_{11}$-(NHC)$_2$(+)
20a = $C_2B_8H_9$-NHC(+)
(d) The two structures of Idip(o-2) differ by the relative orientation of the two Idip groups, NIMAG=0.
(e) The two structures of Idip(m-2) differ by the relative orientation of the two Idip group, NIMAG=0.
(f) The energy of the cation entries (notation ending with "a") are with respect to 2NHC+1,2-$C_2B_8H_{10}$ + 2HCl or Idip2 +1,2-$C_2B_8H_{10}$ + 2HCl and yielding product + $ClHCl^-$.

Table S2. Cartesian Coordinates of Stationary Points listed in Table S1 Optimized at the B3LYP/6-31G(d) Level

nhc

Standard orientation:

Center Number	Atomic Number	Atomic Type	Coordinates (Angstroms) X	Y	Z
1	6	0	0.000000	0.000000	1.285368
2	7	0	-0.000000	1.049834	0.402871
3	1	0	-0.000000	2.006592	0.722116
4	6	0	-0.000000	0.677956	-0.940358
5	1	0	-0.000000	1.384836	-1.756166
6	7	0	-0.000000	-1.049834	0.402871
7	1	0	-0.000000	-2.006592	0.722116
8	6	0	-0.000000	-0.677956	-0.940358
9	1	0	-0.000000	-1.384836	-1.756166

o

Standard orientation:

Center Number	Atomic Number	Atomic Type	Coordinates (Angstroms) X	Y	Z
1	6	0	-0.885960	1.385836	0.000000
2	5	0	-0.354757	0.634905	1.317075
3	5	0	0.721068	1.333995	0.000000
4	5	0	-0.354757	-1.145914	0.916692
5	5	0	-0.354757	-1.145914	-0.916692
6	5	0	1.199044	-0.145125	0.927351
7	5	0	1.199044	-0.145125	-0.927351
8	1	0	-1.585602	2.208893	0.000000
9	1	0	-2.408401	-0.235563	0.000000
10	1	0	-0.758009	0.996298	-2.369171
11	1	0	1.316942	2.357588	0.000000
12	1	0	-0.912722	-1.855599	-1.687246
13	1	0	2.077355	-0.068376	1.724660
14	1	0	2.077355	-0.068376	-1.724660
15	1	0	1.638300	-2.572154	-0.000000

16	5	0	1.010895	-1.567689	-0.000000
17	6	0	-1.335639	-0.081436	0.000000
18	5	0	-0.354757	0.634905	-1.317075
19	1	0	-0.758009	0.996298	2.369171
20	1	0	-0.912722	-1.855599	1.687246

TSo/1

Standard orientation:

Center Number	Atomic Number	Atomic Type	Coordinates (Angstroms) X	Y	Z
1	6	0	1.146232	-0.666904	-1.317598
2	6	0	2.344204	-1.168415	-0.495311
3	5	0	0.458914	-0.823463	0.119669
4	5	0	2.426042	0.350834	-1.334817
5	5	0	0.783544	0.792582	-0.709362
6	5	0	2.161886	-0.905346	1.144224
7	5	0	3.397002	0.020653	0.155794
8	5	0	0.997669	0.565971	1.085758
9	5	0	2.253486	1.459366	0.058996
10	5	0	2.647665	0.663595	1.531032
11	1	0	0.831963	-1.146709	-2.233961
12	1	0	2.784495	-2.094324	-0.848217
13	1	0	-0.079355	-1.835183	0.412385
14	1	0	2.952983	0.533500	-2.379816
15	1	0	-0.019969	1.465862	-1.262653
16	1	0	2.264055	-1.840827	1.871220
17	1	0	4.564337	-0.148457	0.005001
18	1	0	0.205144	0.993821	1.864749
19	1	0	2.465341	2.622295	-0.080285
20	1	0	3.117548	1.115055	2.522149
21	6	0	-1.979349	-0.198780	-0.160122
22	7	0	-2.954634	-1.053158	0.254589
23	7	0	-2.683799	0.952084	-0.331950
24	1	0	-2.756006	-2.014165	0.492650
25	1	0	-2.233207	1.807527	-0.624597
26	6	0	-4.036505	0.828104	-0.034996
27	6	0	-4.212014	-0.463844	0.342372
28	1	0	-4.738271	1.643881	-0.115321
29	1	0	-5.096481	-0.996693	0.655694

1

Standard orientation:

Center Number	Atomic Number	Atomic Type	Coordinates (Angstroms) X	Y	Z
1	6	0	-0.782985	-0.453294	-1.241717
2	6	0	-2.114869	0.312907	-1.316732
3	5	0	0.174665	0.447516	-0.273292
4	5	0	-2.158296	-1.251562	-0.627474
5	5	0	-0.694017	-1.020824	0.341546

6	5	0	-2.237006	1.440824	-0.182534
7	5	0	-3.311240	-0.020957	-0.065185
8	5	0	-0.998860	0.455088	1.262818
9	5	0	-2.262895	-0.872844	1.120999
10	5	0	-2.676598	0.819846	1.285289
11	1	0	-0.435350	-0.999455	-2.115098
12	1	0	-2.511153	0.464783	-2.316136
13	1	0	0.094351	1.616526	-0.543772
14	1	0	-2.488941	-2.231896	-1.208581
15	1	0	0.057936	-1.914167	0.594596
16	1	0	-2.313790	2.595876	-0.471640
17	1	0	-4.446428	-0.164187	-0.394113
18	1	0	-0.402378	0.789636	2.243338
19	1	0	-2.572708	-1.714714	1.905180
20	1	0	-3.222238	1.341502	2.205016
21	6	0	1.732843	0.150244	-0.093407
22	7	0	2.681643	1.106537	-0.212777
23	7	0	2.433469	-0.969755	0.187994
24	1	0	2.440208	2.066328	-0.421726
25	1	0	1.966054	-1.847900	0.376327
26	6	0	3.794199	-0.723515	0.252145
27	6	0	3.953302	0.602795	-0.001982
28	1	0	4.515457	-1.495065	0.469297
29	1	0	4.839489	1.215009	-0.049878

TS1/o-2

Standard orientation:

Center Number	Atomic Number	Atomic Type	Coordinates (Angstroms) X	Y	Z
1	6	0	-1.076986	0.577219	-1.286687
2	6	0	0.455337	0.442943	-1.299058
3	5	0	-1.714353	-0.253923	-0.041365
4	5	0	-0.167034	1.997342	-1.003558
5	5	0	-1.503552	1.531815	0.075134
6	5	0	1.009596	-0.290106	0.012108
7	5	0	1.313982	1.509906	-0.164585
8	5	0	-0.796950	0.663725	1.410070
9	5	0	-0.025599	2.169941	0.771304
10	5	0	0.910114	0.767574	1.336699
11	1	0	-1.571381	0.678542	-2.250494
12	1	0	0.908492	0.274942	-2.270093
13	1	0	-1.164531	-1.306977	0.141363
14	1	0	-0.202686	2.834331	-1.845418
15	1	0	-2.541579	2.128335	0.035051
16	1	0	1.158412	-1.464243	0.109131
17	1	0	2.325637	2.013963	-0.528972
18	1	0	-1.340845	0.526720	2.467593
19	1	0	-0.028138	3.251622	1.273943
20	1	0	1.585581	0.761640	2.330630
21	6	0	-3.274614	-0.538047	0.015587
22	7	0	-3.807542	-1.762256	0.239291

23	7	0	-4.353836	0.264084	-0.126680
24	1	0	-3.220940	-2.572239	0.387981
25	1	0	-4.247086	1.263655	-0.248132
26	6	0	-5.539827	-0.437585	0.012857
27	6	0	-5.192202	-1.730487	0.247444
28	1	0	-6.506870	0.033192	-0.063396
29	1	0	-5.795543	-2.608089	0.415327
30	7	0	4.656004	-1.126430	-1.016506
31	7	0	4.069984	-0.194319	0.779840
32	6	0	3.567225	-0.540481	-0.435795
33	6	0	5.785634	-1.139591	-0.200024
34	1	0	6.733172	-1.558662	-0.502666
35	6	0	5.402706	-0.539314	0.955893
36	1	0	5.952756	-0.334697	1.861645
37	1	0	3.480263	0.263298	1.466556
38	1	0	4.628363	-1.502206	-1.951990

----

o-2

Standard orientation:

Center Number	Atomic Number	Atomic Type	Coordinates (Angstroms) X	Y	Z
1	5	0	0.172213	0.127094	1.485086
2	5	0	-1.633692	0.118691	1.394182
3	5	0	-0.902875	1.598049	-0.847444
4	5	0	-2.330010	0.878900	0.000000
5	5	0	0.172213	0.127094	-1.485086
6	5	0	-2.088010	-0.876821	0.000000
7	5	0	-1.633692	0.118691	-1.394182
8	6	0	-0.579405	-1.142917	-0.764345
9	1	0	-0.977534	2.566745	1.554178
10	1	0	1.196200	0.441961	0.934372
11	1	0	-2.189443	-0.123332	2.432362
12	1	0	-0.977534	2.566745	-1.554178
13	1	0	-3.443770	1.310892	0.000000
14	1	0	-0.571980	-2.128660	1.224789
15	1	0	1.196200	0.441961	-0.934372
16	1	0	-2.894207	-1.751584	0.000000
17	1	0	-2.189443	-0.123332	-2.432362
18	1	0	-0.571980	-2.128660	-1.224789
19	6	0	0.531164	-0.018785	-3.025803
20	7	0	-0.214299	-0.339542	-4.108811
21	1	0	-1.211367	-0.494354	-4.018308
22	6	0	0.528076	-0.302638	-5.277757
23	1	0	0.103639	-0.522339	-6.244383
24	7	0	1.763954	0.208458	-3.540470
25	1	0	2.533552	0.485112	-2.946831
26	6	0	1.790638	0.047124	-4.916637
27	1	0	2.682311	0.191734	-5.505288
28	6	0	0.531164	-0.018785	3.025803
29	7	0	-0.214299	-0.339542	4.108811
30	1	0	-1.211367	-0.494354	4.018308

31	6	0	0.528076	-0.302638	5.277757
32	1	0	0.103639	-0.522339	6.244383
33	7	0	1.763954	0.208458	3.540470
34	1	0	2.533552	0.485112	2.946831
35	6	0	1.790638	0.047124	4.916637
36	1	0	2.682311	0.191734	5.505288
37	6	0	-0.579405	-1.142917	0.764345
38	5	0	-0.902875	1.598049	0.847444

TS1/2

Standard orientation:

Center Number	Atomic Number	Atomic Type	Coordinates (Angstroms) X	Y	Z
1	7	0	-2.234570	0.702665	0.688418
2	1	0	4.121298	-0.434116	-0.843721
3	7	0	-2.599549	-0.818992	-0.757380
4	1	0	2.955647	-0.515751	2.369326
5	1	0	2.712914	-2.444675	0.167567
6	6	0	-1.599626	-0.181342	-0.108265
7	1	0	0.622107	-1.968322	1.542019
8	1	0	1.136585	-0.378289	-2.770839
9	1	0	-0.022684	-1.384925	-1.098440
10	6	0	-3.607120	0.634205	0.538856
11	1	0	-4.284941	1.264047	1.092511
12	1	0	2.938741	2.069421	-1.792124
13	1	0	-0.064216	2.092055	-0.607543
14	1	0	2.625911	2.200775	1.175186
15	6	0	-3.842723	-0.335112	-0.386275
16	1	0	-4.763839	-0.713592	-0.799364
17	6	0	1.018291	-1.212183	0.871431
18	6	0	2.263717	-1.457884	0.137783
19	5	0	-0.041837	-0.462436	-0.325911
20	1	0	0.140776	0.709807	2.069451
21	5	0	0.831250	0.384192	1.148144
22	5	0	0.739937	1.212508	-0.490424
23	5	0	1.395915	0.106498	-1.720464
24	5	0	2.349868	1.263857	-1.142277
25	5	0	2.229401	1.271006	0.539873
26	5	0	2.972617	-0.252259	-0.569262
27	5	0	2.414070	-0.356705	1.323925
28	1	0	-2.415895	-1.545301	-1.436787
29	1	0	-1.714915	1.343739	1.276300

2

Standard orientation:

Center Number	Atomic Number	Atomic Type	Coordinates (Angstroms) X	Y	Z
1	7	0	-2.254925	-0.953581	0.457234
2	1	0	4.174880	0.432856	-0.944041

3	7	0	-2.491022	0.879204	-0.615836
4	1	0	2.881594	-2.457310	0.400183
5	1	0	2.476164	-1.316429	-2.282149
6	6	0	-1.554248	0.031499	-0.142079
7	1	0	0.424915	-2.065635	-1.053329
8	1	0	-0.001655	2.778032	-0.475879
9	1	0	0.348662	0.950015	-1.222106
10	6	0	-3.619657	-0.720077	0.381531
11	1	0	-4.347829	-1.389847	0.809727
12	1	0	2.925679	2.543027	0.711907
13	1	0	0.324555	1.247830	2.194894
14	1	0	3.197685	0.075677	2.082423
15	6	0	-3.769394	0.444943	-0.301993
16	1	0	-4.654096	0.990525	-0.588069
17	6	0	0.953814	-1.182165	-0.703533
18	6	0	2.194754	-0.779214	-1.381139
19	5	0	0.004932	0.198711	-0.206074
20	1	0	0.388626	-1.602962	1.662739
21	5	0	0.973536	-0.847866	0.937463
22	5	0	0.861430	0.893136	1.188909
23	5	0	0.730965	1.841648	-0.305548
24	5	0	2.241521	1.656239	0.295972
25	5	0	2.488658	0.124284	1.123692
26	5	0	3.043551	0.222377	-0.625453
27	5	0	2.433296	-1.385841	0.150126
28	1	0	-2.242512	1.755987	-1.057721
29	1	0	-1.796089	-1.704485	0.959480

TS2/o-1

Standard orientation:

Center Number	Atomic Number	Atomic Type	Coordinates (Angstroms) X	Y	Z
1	7	0	-2.429639	0.985313	-0.257043
2	1	0	4.232424	0.166765	-1.000138
3	7	0	-2.342380	-1.110252	0.150226
4	1	0	2.886706	-2.350295	0.827784
5	1	0	2.403826	-1.681961	-2.032779
6	6	0	-1.560092	-0.030182	-0.081804
7	1	0	0.423497	-2.196452	-0.640358
8	1	0	-0.117757	2.650523	-0.802835
9	1	0	0.661879	0.921127	-1.544886
10	6	0	-3.738762	0.563969	-0.124145
11	1	0	-4.579088	1.231507	-0.226997
12	1	0	2.958041	2.625592	0.045958
13	1	0	0.422239	1.715320	1.893025
14	1	0	3.285453	0.512812	1.953076
15	6	0	-3.686439	-0.770290	0.137604
16	1	0	-4.469872	-1.490772	0.309894
17	6	0	0.936209	-1.254376	-0.451786
18	6	0	2.178878	-0.999483	-1.217514
19	5	0	-0.004510	0.130824	-0.151402

20	1	0	0.502173	-1.269767	2.021582
21	5	0	1.003651	-0.654694	1.130060
22	5	0	0.884904	1.095787	0.984222
23	5	0	0.624980	1.715838	-0.624378
24	5	0	2.219016	1.696112	-0.062066
25	5	0	2.539738	0.341848	1.036817
26	5	0	3.107580	0.024325	-0.633230
27	5	0	2.437726	-1.333066	0.411064
28	1	0	-1.966585	-2.023349	0.366617
29	1	0	-2.082993	1.920829	-0.451792

----

o-1

Standard orientation:

Center Number	Atomic Number	Atomic Type	Coordinates (Angstroms) X	Y	Z
1	7	0	-2.510665	0.942716	-0.464746
2	1	0	4.341842	0.083522	-0.757683
3	7	0	-2.285949	-1.024152	0.333591
4	1	0	2.703225	-2.501162	0.610415
5	1	0	2.483645	-1.515994	-2.165418
6	6	0	-1.571750	0.038029	-0.108459
7	1	0	0.338670	-1.960531	-1.029929
8	1	0	0.100672	2.809130	-0.684429
9	1	0	1.771416	1.671629	-1.084560
10	6	0	-3.791990	0.470091	-0.236295
11	1	0	-4.674521	1.050001	-0.453428
12	1	0	2.984838	2.564422	0.306107
13	1	0	0.476376	1.516006	2.118856
14	1	0	3.169864	0.220317	2.071991
15	6	0	-3.650140	-0.782941	0.271253
16	1	0	-4.384373	-1.507988	0.583805
17	6	0	0.908483	-1.095690	-0.692225
18	6	0	2.226342	-0.891197	-1.314772
19	5	0	-0.023345	0.244893	-0.211697
20	1	0	0.306654	-1.421494	1.760266
21	5	0	0.889479	-0.726626	0.978405
22	5	0	0.914671	1.027392	1.122690
23	5	0	0.625984	1.760211	-0.443532
24	5	0	2.257610	1.635597	0.151264
25	5	0	2.482258	0.133445	1.101971
26	5	0	3.179743	-0.026823	-0.522943
27	5	0	2.337510	-1.424699	0.266567
28	1	0	-1.839101	-1.830670	0.750208
29	1	0	-2.248130	1.846164	-0.838753

----

p

Standard orientation:

Center Number	Atomic Number	Atomic Type	Coordinates (Angstroms) X	Y	Z

1	6	0	0.000000	0.000000	1.672727
2	5	0	0.000000	1.310868	0.753749
3	5	0	-1.310868	0.000000	0.753749
4	5	0	0.926924	-0.926924	-0.753749
5	5	0	-0.926924	0.926924	-0.753749
6	5	0	-0.926924	-0.926924	-0.753749
7	1	0	0.000000	0.000000	2.753953
8	1	0	2.387654	-0.000000	1.249883
9	1	0	-0.000000	-2.387654	1.249883
10	1	0	-2.387654	-0.000000	1.249883
11	1	0	1.688326	-1.688326	-1.249883
12	1	0	-1.688326	1.688326	-1.249883
13	1	0	-1.688326	-1.688326	-1.249883
14	1	0	0.000000	0.000000	-2.753953
15	5	0	-0.000000	-1.310868	0.753749
16	1	0	0.000000	2.387654	1.249883
17	1	0	1.688326	1.688326	-1.249883
18	5	0	1.310868	-0.000000	0.753749
19	5	0	0.926924	0.926924	-0.753749
20	6	0	0.000000	0.000000	-1.672727

TSp/13

Standard orientation:

Center Number	Atomic Number	Atomic Type	Coordinates (Angstroms) X	Y	Z
1	6	0	-1.045177	-0.425277	1.454291
2	5	0	-0.287548	-0.794361	0.077736
3	5	0	-2.426397	0.449857	1.282334
4	5	0	-0.793806	0.956002	0.680520
5	5	0	-1.910288	-1.072529	-1.029021
6	5	0	-3.267990	-0.149347	-0.182665
7	5	0	-2.243651	1.401407	-0.244563
8	1	0	-0.665807	-0.649938	2.443793
9	1	0	-2.624477	-2.252382	1.235511
10	1	0	0.230528	-1.867510	-0.074361
11	1	0	-2.971728	0.850733	2.257714
12	1	0	-0.059480	1.782843	1.123794
13	1	0	-1.933830	-1.977866	-1.798320
14	1	0	-4.443281	-0.304041	-0.272321
15	1	0	-0.144252	0.918897	-1.921892
16	1	0	-2.565316	2.536708	-0.384435
17	1	0	-2.848623	0.631488	-2.421603
18	6	0	1.787120	-0.111916	0.091272
19	7	0	2.713460	-1.060496	-0.177708
20	7	0	2.540253	1.011790	0.190197
21	1	0	2.445870	-2.026224	-0.318771
22	1	0	2.125222	1.912426	0.385257
23	6	0	3.894869	0.776003	-0.011800
24	6	0	4.005674	-0.557412	-0.248640
25	1	0	4.643374	1.552237	0.030348
26	1	0	4.869174	-1.170593	-0.454624

27	5	0	-2.208148	-1.258268	0.731350
28	6	0	-2.432060	0.357970	-1.461713
29	5	0	-0.881356	0.500829	-1.087286

13

Standard orientation:

Center Number	Atomic Number	Atomic Type	Coordinates (Angstroms) X	Y	Z
1	6	0	0.807510	-0.315423	1.351522
2	5	0	-0.136824	0.511471	0.257143
3	5	0	2.195525	-1.140387	0.845509
4	5	0	0.693122	-1.097850	-0.094833
5	5	0	2.224530	1.494652	-0.081994
6	5	0	3.265810	0.016185	0.083365
7	5	0	2.209218	-1.019270	-0.968601
8	1	0	0.378513	-0.746672	2.253598
9	1	0	2.610309	0.921195	2.446695
10	1	0	-0.165364	1.700778	0.460286
11	1	0	2.536932	-2.100778	1.456772
12	1	0	-0.046640	-2.026231	-0.237808
13	1	0	2.304757	2.678444	-0.177335
14	1	0	4.454562	0.026536	0.131233
15	1	0	0.357863	0.351056	-2.327329
16	1	0	2.567084	-1.865645	-1.721698
17	1	0	2.989795	0.842431	-2.210973
18	6	0	-1.706283	0.164380	0.075552
19	7	0	-2.663168	1.117034	0.147481
20	7	0	-2.401255	-0.973978	-0.142468
21	1	0	-2.423514	2.085607	0.315286
22	1	0	-1.929490	-1.861627	-0.262518
23	6	0	-3.764512	-0.742552	-0.210718
24	6	0	-3.932196	0.594485	-0.027593
25	1	0	-4.481342	-1.530303	-0.379517
26	1	0	-4.823031	1.201471	-0.005180
27	5	0	2.165761	0.569146	1.395658
28	6	0	2.506722	0.563061	-1.281576
29	5	0	0.913477	0.213781	-1.280192

TS13/p-2

Standard orientation:

Center Number	Atomic Number	Atomic Type	Coordinates (Angstroms) X	Y	Z
1	6	0	-1.018247	0.715641	-1.220504
2	5	0	-1.663777	-0.220656	-0.001937
3	5	0	-0.139592	2.129012	-0.812073
4	5	0	-1.505533	1.565808	0.172746
5	5	0	1.151127	-0.275437	0.076520
6	5	0	1.275020	1.519412	-0.017746
7	5	0	-0.064498	2.101090	0.984801

8	1	0	-1.597423	0.845754	-2.134288
9	1	0	1.102982	0.552943	-2.447468
10	1	0	-1.117454	-1.289490	0.112189
11	1	0	-0.224321	3.118086	-1.469103
12	1	0	-2.545454	2.162267	0.188703
13	1	0	0.984569	-1.458030	0.078955
14	1	0	2.348632	2.044304	0.000004
15	1	0	-1.382722	0.504358	2.572148
16	1	0	0.046646	3.056306	1.685670
17	1	0	1.316661	0.636147	2.254231
18	6	0	-3.218165	-0.530150	-0.029184
19	7	0	-3.746697	-1.773630	0.083139
20	7	0	-4.302455	0.265942	-0.185310
21	1	0	-3.155240	-2.584360	0.201848
22	1	0	-4.202497	1.271419	-0.247293
23	6	0	-5.483763	-0.457927	-0.161880
24	6	0	-5.130595	-1.758535	0.010423
25	1	0	-6.452051	0.004680	-0.267382
26	1	0	-5.729345	-2.652199	0.083390
27	5	0	0.593915	0.608439	-1.366271
28	6	0	0.730783	0.632866	1.338627
29	5	0	-0.882782	0.605563	1.490779
30	6	0	3.434657	-0.545796	0.027539
31	7	0	4.090638	-1.730086	0.076605
32	1	0	3.600990	-2.606125	0.188014
33	6	0	5.468034	-1.588901	-0.056211
34	1	0	6.152061	-2.423070	-0.041064
35	7	0	4.437842	0.344553	-0.137204
36	1	0	4.242913	1.333931	-0.222033
37	6	0	5.689213	-0.255635	-0.193563
38	1	0	6.604847	0.300098	-0.322700

----

p-2

Standard orientation:

Center Number	Atomic Number	Atomic Type	Coordinates (Angstroms) X	Y	Z
1	6	0	-1.260386	-0.892096	0.743139
2	5	0	0.006634	-1.491014	-0.170949
3	5	0	0.130815	-1.381770	1.619439
4	5	0	-1.461731	0.704767	0.704026
5	5	0	-0.889288	-0.059528	2.205038
6	5	0	1.461731	-0.704767	0.704026
7	5	0	-0.006634	1.491014	-0.170949
8	5	0	0.889288	0.059528	2.205038
9	5	0	-0.130815	1.381770	1.619439
10	6	0	1.260386	0.892096	0.743139
11	1	0	-2.154861	-1.507711	0.833985
12	1	0	0.144099	-0.903349	-1.216654
13	1	0	0.143683	-2.435346	2.194481
14	1	0	-2.554051	1.192353	0.655826
15	1	0	-1.562524	-0.177358	3.180070

16	1	0	2.554051	-1.192353	0.655826
17	1	0	-0.144099	0.903349	-1.216654
18	1	0	1.562524	0.177358	3.180070
19	1	0	-0.143683	2.435346	2.194481
20	1	0	2.154861	1.507711	0.833985
21	6	0	-0.002723	3.033633	-0.525580
22	7	0	0.152515	4.140559	0.239894
23	1	0	0.235903	4.064217	1.246072
24	6	0	0.090351	5.303097	-0.511404
25	1	0	0.188529	6.284210	-0.074896
26	7	0	-0.152515	3.530269	-1.779197
27	1	0	-0.284255	2.917333	-2.571375
28	6	0	-0.105142	4.915580	-1.798785
29	1	0	-0.209759	5.491299	-2.704382
30	6	0	0.002723	-3.033633	-0.525580
31	7	0	-0.152515	-4.140559	0.239894
32	1	0	-0.235903	-4.064217	1.246072
33	6	0	-0.090351	-5.303097	-0.511404
34	1	0	-0.188529	-6.284210	-0.074896
35	7	0	0.152515	-3.530269	-1.779197
36	1	0	0.284255	-2.917333	-2.571375
37	6	0	0.105142	-4.915580	-1.798785
38	1	0	0.209759	-5.491299	-2.704382

TSo-2/3

Standard orientation:

Center Number	Atomic Number	Atomic Type	Coordinates (Angstroms) X	Y	Z
1	5	0	-1.680448	0.337585	-0.118186
2	5	0	-1.425025	2.014701	0.304857
3	5	0	1.032290	0.661642	1.319998
4	5	0	0.183593	2.166613	1.095642
5	5	0	1.361267	-0.263540	-0.227698
6	5	0	0.012422	2.291792	-0.800498
7	5	0	1.566447	1.954761	0.072681
8	6	0	1.249958	1.051124	-1.142813
9	1	0	-1.385157	0.461104	2.435825
10	1	0	-0.760088	-0.299487	0.594055
11	1	0	-2.262823	2.842092	0.490756
12	1	0	1.594042	0.489733	2.370244
13	1	0	0.204887	3.044884	1.905297
14	1	0	-1.703979	1.519163	-2.089656
15	1	0	0.682493	-1.277741	-0.455807
16	1	0	0.081878	3.312138	-1.419145
17	1	0	2.372047	2.855212	0.111477
18	1	0	1.552713	1.181942	-2.184099
19	6	0	2.871406	-0.729042	-0.170095
20	7	0	4.002845	0.015676	-0.078061
21	1	0	3.954641	1.026922	-0.039876
22	6	0	5.134466	-0.775716	0.048523
23	1	0	6.125824	-0.362185	0.144351

24	7	0	3.325777	-2.006713	-0.125263
25	1	0	2.687812	-2.787611	-0.174654
26	6	0	4.707403	-2.063909	0.018212
27	1	0	5.250895	-2.992998	0.081642
28	6	0	-3.031610	-0.419209	-0.095143
29	7	0	-3.858832	-0.756912	-1.114880
30	1	0	-3.652511	-0.531005	-2.077620
31	6	0	-4.975192	-1.441069	-0.655798
32	1	0	-5.751915	-1.793734	-1.315002
33	7	0	-3.643165	-0.911312	1.009156
34	1	0	-3.250504	-0.762192	1.931715
35	6	0	-4.836902	-1.536979	0.692763
36	1	0	-5.472200	-1.988515	1.437635
37	6	0	-1.161922	1.276639	-1.176483
38	5	0	-0.810559	0.737660	1.413446

3

Standard orientation:

Center Number	Atomic Number	Atomic Type	Coordinates (Angstroms) X	Y	Z
1	5	0	-1.717833	0.345713	-0.139525
2	5	0	-1.421041	1.999562	0.389281
3	5	0	1.047654	0.564930	1.284472
4	5	0	0.175038	2.055927	1.200119
5	5	0	1.416262	-0.228476	-0.315317
6	5	0	-0.011634	2.256194	-0.820564
7	5	0	1.559265	2.062887	0.153955
8	6	0	1.399884	1.190742	-1.078341
9	1	0	-1.373684	0.245920	2.388161
10	1	0	-0.748718	-0.331947	0.486102
11	1	0	-2.246770	2.807744	0.685043
12	1	0	1.621216	0.343285	2.320089
13	1	0	0.163933	2.854344	2.090318
14	1	0	-1.907573	1.700112	-1.968847
15	1	0	0.732829	-1.193781	-0.675953
16	1	0	0.039985	3.333987	-1.340558
17	1	0	2.240611	3.047747	0.300607
18	1	0	1.801920	1.403511	-2.075385
19	6	0	2.910997	-0.725469	-0.222256
20	7	0	4.045447	0.009236	-0.082035
21	1	0	4.005646	1.019912	-0.041682
22	6	0	5.168388	-0.791722	0.064748
23	1	0	6.159053	-0.386907	0.197774
24	7	0	3.358065	-2.007501	-0.188883
25	1	0	2.720368	-2.784916	-0.276040
26	6	0	4.734576	-2.075864	-0.003904
27	1	0	5.270803	-3.009387	0.056881
28	6	0	-3.072031	-0.423219	-0.103682
29	7	0	-3.931586	-0.687679	-1.113813
30	1	0	-3.753290	-0.393292	-2.064365
31	6	0	-5.034489	-1.397781	-0.666334

32	1	0	-5.835274	-1.701991	-1.320919
33	7	0	-3.644593	-0.985850	0.983427
34	1	0	-3.216722	-0.898086	1.898997
35	6	0	-4.850336	-1.586298	0.668215
36	1	0	-5.461880	-2.085734	1.402348
37	6	0	-1.275977	1.361958	-1.147813
38	5	0	-0.825681	0.626856	1.384132

TS3/4

Standard orientation:

Center Number	Atomic Number	Atomic Type	Coordinates (Angstroms) X	Y	Z
1	5	0	-1.733606	0.308290	-0.154283
2	5	0	-1.384769	1.941742	0.441080
3	5	0	1.061168	0.466847	1.279212
4	5	0	0.159258	1.924420	1.347751
5	5	0	1.465704	-0.215701	-0.353561
6	5	0	-0.006901	2.181774	-0.924158
7	5	0	1.491828	2.114907	0.266636
8	6	0	1.458279	1.269405	-0.994891
9	1	0	-1.379217	0.031947	2.332128
10	1	0	-0.764874	-0.409005	0.392291
11	1	0	-2.220157	2.726714	0.772970
12	1	0	1.681940	0.225760	2.283657
13	1	0	0.110851	2.621493	2.319188
14	1	0	-2.019633	1.767409	-1.864202
15	1	0	0.794179	-1.145413	-0.802159
16	1	0	0.029209	3.289975	-1.380402
17	1	0	2.090373	3.141149	0.466543
18	1	0	1.982246	1.555332	-1.916969
19	6	0	2.959695	-0.709495	-0.255415
20	7	0	4.081287	0.038932	-0.086096
21	1	0	4.026529	1.048093	-0.035011
22	6	0	5.215560	-0.747303	0.054171
23	1	0	6.198564	-0.330656	0.206078
24	7	0	3.426881	-1.985213	-0.245789
25	1	0	2.804480	-2.771644	-0.357973
26	6	0	4.802272	-2.036005	-0.048224
27	1	0	5.352799	-2.962128	-0.003905
28	6	0	-3.110397	-0.426270	-0.111220
29	7	0	-4.014631	-0.577707	-1.104226
30	1	0	-3.858232	-0.211963	-2.033903
31	6	0	-5.126830	-1.282395	-0.672517
32	1	0	-5.962268	-1.503399	-1.317091
33	7	0	-3.662850	-1.054960	0.948530
34	1	0	-3.202198	-1.048874	1.852404
35	6	0	-4.900891	-1.585804	0.634408
36	1	0	-5.503555	-2.121623	1.350020
37	6	0	-1.314502	1.361654	-1.136883
38	5	0	-0.827673	0.495070	1.363886

4

Standard orientation:

Center Number	Atomic Number	Atomic Type	Coordinates (Angstroms) X	Y	Z
1	5	0	-1.404060	0.165579	-0.437757
2	5	0	-0.945963	1.067483	1.096100
3	5	0	1.615663	1.903192	0.018154
4	5	0	0.685384	1.289553	1.416816
5	5	0	1.279953	-0.034787	-0.695649
6	5	0	-0.235011	2.126015	-1.501380
7	5	0	-0.119796	2.534491	0.680668
8	6	0	1.205407	1.612977	-1.411074
9	1	0	0.099872	-1.254851	1.292277
10	1	0	-1.166542	-0.253240	-1.552695
11	1	0	-1.796288	1.036717	1.943957
12	1	0	2.732322	2.225984	0.315744
13	1	0	1.243705	1.362654	2.477332
14	1	0	-2.190966	2.473386	-0.419325
15	1	0	1.046683	-0.624506	-1.740134
16	1	0	-0.580509	2.874001	-2.377007
17	1	0	-0.163915	3.705345	0.898451
18	1	0	1.901867	1.760831	-2.235729
19	6	0	2.717697	-0.577516	-0.239454
20	7	0	3.310409	-0.520809	0.975126
21	1	0	2.874828	-0.002451	1.731885
22	6	0	4.474651	-1.265378	1.019110
23	1	0	5.084726	-1.341099	1.905122
24	7	0	3.542528	-1.373401	-0.962843
25	1	0	3.330803	-1.616016	-1.920286
26	6	0	4.626841	-1.812702	-0.216125
27	1	0	5.391411	-2.458305	-0.617607
28	6	0	-2.836673	-0.465995	-0.119845
29	7	0	-3.451052	-1.340647	-0.953008
30	1	0	-3.023288	-1.603579	-1.830671
31	6	0	-4.655453	-1.791804	-0.439390
32	1	0	-5.285814	-2.496348	-0.957881
33	7	0	-3.691775	-0.366700	0.925450
34	1	0	-3.455657	0.197866	1.732203
35	6	0	-4.808260	-1.169010	0.759182
36	1	0	-5.599009	-1.225310	1.490328
37	6	0	-1.280519	1.880141	-0.351959
38	5	0	0.108401	-0.254414	0.616707

TS4/5

Standard orientation:

Center Number	Atomic Number	Atomic Type	Coordinates (Angstroms) X	Y	Z
1	5	0	1.422860	0.176729	0.457509
2	5	0	0.941383	1.010984	-1.145242

3	5	0	-1.612956	1.940664	-0.025397
4	5	0	-0.709083	1.081675	-1.390474
5	5	0	-1.290290	0.024185	0.606455
6	5	0	0.199601	2.261089	1.344207
7	5	0	0.049792	2.447417	-0.809508
8	6	0	-1.208278	1.658431	1.395777
9	1	0	-0.008415	-1.392504	-1.177499
10	1	0	1.224697	-0.136046	1.618053
11	1	0	1.774823	0.966149	-2.008207
12	1	0	-2.718648	2.262606	-0.359450
13	1	0	-1.358914	1.037133	-2.399585
14	1	0	2.152746	2.517779	0.256385
15	1	0	-1.049719	-0.439988	1.710282
16	1	0	0.481662	3.163039	2.087854
17	1	0	0.086860	3.569066	-1.209391
18	1	0	-1.884781	1.791403	2.237000
19	6	0	-2.720886	-0.567723	0.215485
20	7	0	-3.299381	-0.652147	-1.003115
21	1	0	-2.851608	-0.228757	-1.809974
22	6	0	-4.484356	-1.363672	-0.965435
23	1	0	-5.088331	-1.536320	-1.841916
24	7	0	-3.572462	-1.242450	1.025574
25	1	0	-3.379546	-1.366483	2.009340
26	6	0	-4.662632	-1.743092	0.327801
27	1	0	-5.448577	-2.310688	0.799581
28	6	0	2.857635	-0.450611	0.154183
29	7	0	3.515782	-1.241866	1.036609
30	1	0	3.121649	-1.435583	1.947186
31	6	0	4.711048	-1.716516	0.521491
32	1	0	5.371136	-2.364376	1.075698
33	7	0	3.677747	-0.425640	-0.923878
34	1	0	3.408059	0.069765	-1.764634
35	6	0	4.813865	-1.193998	-0.728840
36	1	0	5.582459	-1.297682	-1.478203
37	6	0	1.260298	1.894472	0.251113
38	5	0	-0.014811	-0.350250	-0.571208

5

Standard orientation:

Center Number	Atomic Number	Atomic Type	Coordinates (Angstroms) X	Y	Z
1	5	0	-1.529480	0.141889	-0.491532
2	5	0	-0.950867	0.750065	1.109407
3	5	0	1.455825	2.108359	0.223388
4	5	0	0.810561	0.812523	1.344229
5	5	0	1.561610	0.345204	-0.149225
6	5	0	0.013967	2.323626	-0.949266
7	5	0	-0.122646	2.285982	0.919919
8	6	0	1.217373	1.360368	-1.235530
9	1	0	-0.003551	-1.495380	1.490557
10	1	0	-1.167700	-0.207155	-1.582994

11	1	0	-1.709305	0.618770	2.033930
12	1	0	2.375604	2.801793	0.549134
13	1	0	1.275768	0.642836	2.432517
14	1	0	-2.187717	2.368794	-0.085488
15	1	0	0.333924	-1.220173	-0.417744
16	1	0	-0.111508	3.357277	-1.546397
17	1	0	-0.410972	3.157756	1.681582
18	1	0	1.878670	1.555381	-2.081641
19	6	0	2.909970	-0.442047	-0.106494
20	7	0	3.156348	-1.574830	0.585704
21	1	0	2.434137	-2.001354	1.155017
22	6	0	4.466758	-1.994835	0.424231
23	1	0	4.857800	-2.880384	0.898687
24	7	0	4.081701	-0.157572	-0.717796
25	1	0	4.195285	0.665923	-1.293361
26	6	0	5.057261	-1.091728	-0.402907
27	1	0	6.063418	-1.034203	-0.785935
28	6	0	-2.994430	-0.426717	-0.264351
29	7	0	-3.685189	-1.161146	-1.168035
30	1	0	-3.288030	-1.391781	-2.068483
31	6	0	-4.941319	-1.510205	-0.697880
32	1	0	-5.639896	-2.095019	-1.274473
33	7	0	-3.843857	-0.314821	0.781967
34	1	0	-3.550911	0.155318	1.631023
35	6	0	-5.039427	-0.971537	0.546749
36	1	0	-5.841029	-0.998261	1.267533
37	6	0	-1.303760	1.739099	-0.167684
38	5	0	0.020716	-0.670835	0.602448

TS5/6

Standard orientation:

Center Number	Atomic Number	Atomic Type	Coordinates (Angstroms) X	Y	Z
1	5	0	-1.446486	0.228854	-0.509299
2	5	0	-0.948899	0.935748	1.205044
3	5	0	1.496804	2.103394	0.183083
4	5	0	0.745320	0.881610	1.404557
5	5	0	1.442170	0.294091	-0.136854
6	5	0	0.011830	2.445277	-0.931339
7	5	0	-0.052732	2.405806	0.961272
8	6	0	1.227838	1.500821	-1.267532
9	1	0	-0.006206	-1.549498	0.940801
10	1	0	-1.234341	0.037071	-1.704236
11	1	0	-1.750288	0.832849	2.096029
12	1	0	2.514033	2.652405	0.497853
13	1	0	1.298600	0.687545	2.445535
14	1	0	-2.147736	2.509728	-0.118812
15	1	0	0.598533	0.093525	-1.040592
16	1	0	-0.073768	3.503938	-1.487617
17	1	0	-0.277823	3.331171	1.678256
18	1	0	1.942082	1.630627	-2.075246

19	6	0	2.764925	-0.537368	-0.108840
20	7	0	2.926155	-1.743277	0.471738
21	1	0	2.142321	-2.201435	0.924138
22	6	0	4.235846	-2.181368	0.376286
23	1	0	4.566831	-3.123830	0.781852
24	7	0	3.987575	-0.223377	-0.589246
25	1	0	4.171624	0.664924	-1.037128
26	6	0	4.913471	-1.213736	-0.298262
27	1	0	5.948371	-1.148354	-0.593384
28	6	0	-2.872770	-0.426851	-0.256451
29	7	0	-3.577766	-1.092663	-1.203703
30	1	0	-3.221935	-1.182176	-2.145437
31	6	0	-4.779722	-1.578725	-0.712461
32	1	0	-5.476741	-2.135773	-1.317782
33	7	0	-3.664014	-0.497937	0.840425
34	1	0	-3.363762	-0.087807	1.716896
35	6	0	-4.833678	-1.197216	0.590622
36	1	0	-5.588332	-1.357737	1.344068
37	6	0	-1.269403	1.867882	-0.141928
38	5	0	-0.060840	-0.426187	0.497875

6

Standard orientation:

Center Number	Atomic Number	Atomic Type	Coordinates (Angstroms) X	Y	Z
1	5	0	-1.407077	0.187811	-0.428118
2	5	0	-0.944409	0.894263	1.197819
3	5	0	1.392968	2.060790	0.145307
4	5	0	0.781271	0.955162	1.399394
5	5	0	1.413162	0.282687	-0.140439
6	5	0	-0.007960	2.518005	-0.857137
7	5	0	-0.113025	2.429307	0.921852
8	6	0	1.111083	1.341148	-1.401072
9	1	0	0.018747	-1.508760	1.113258
10	1	0	-1.088253	-0.101230	-1.570958
11	1	0	-1.762891	0.807705	2.075738
12	1	0	2.399308	2.699355	0.183949
13	1	0	1.350451	0.856592	2.449165
14	1	0	-2.212068	2.429958	-0.146733
15	1	0	0.385182	0.791025	-2.006774
16	1	0	-0.078726	3.560150	-1.437563
17	1	0	-0.330765	3.374196	1.613430
18	1	0	1.921650	1.711798	-2.034103
19	6	0	2.754156	-0.489378	-0.071742
20	7	0	3.154386	-1.311706	0.930044
21	1	0	2.569172	-1.459881	1.741804
22	6	0	4.390452	-1.879193	0.666537
23	1	0	4.874816	-2.560531	1.347499
24	7	0	3.766789	-0.557145	-0.974009
25	1	0	3.753500	-0.028494	-1.833981
26	6	0	4.783610	-1.400800	-0.541709

27	1	0	5.674699	-1.582821	-1.120554
28	6	0	-2.839375	-0.472075	-0.226743
29	7	0	-3.480605	-1.213522	-1.162592
30	1	0	-3.068005	-1.378743	-2.070087
31	6	0	-4.706065	-1.676729	-0.708546
32	1	0	-5.360575	-2.285986	-1.310969
33	7	0	-3.693618	-0.471915	0.823162
34	1	0	-3.442526	-0.009530	1.689008
35	6	0	-4.840563	-1.202184	0.557546
36	1	0	-5.636558	-1.317998	1.275733
37	6	0	-1.296170	1.842338	-0.169720
38	5	0	0.017352	-0.427135	0.581041

TS6/7

Standard orientation:

Center Number	Atomic Number	Atomic Type	Coordinates (Angstroms) X	Y	Z
1	5	0	-1.449567	0.195736	-0.461654
2	5	0	-0.894932	0.854411	1.176454
3	5	0	1.640205	1.986995	0.095936
4	5	0	0.798806	0.941562	1.258766
5	5	0	1.336080	0.033764	-0.526946
6	5	0	-0.365950	2.579979	-1.083895
7	5	0	-0.076717	2.377311	0.964334
8	6	0	1.097284	2.200262	-1.272501
9	1	0	-0.005681	-1.485814	1.086567
10	1	0	-1.260382	0.059870	-1.649587
11	1	0	-1.656700	0.711664	2.094206
12	1	0	2.732858	2.329045	0.459359
13	1	0	1.442594	0.771039	2.261429
14	1	0	-2.258565	2.460730	0.086374
15	1	0	1.192540	-0.025606	-1.735560
16	1	0	-0.788815	3.553565	-1.650657
17	1	0	-0.143350	3.375071	1.607749
18	1	0	1.698842	2.498955	-2.133486
19	6	0	2.742450	-0.608940	-0.210179
20	7	0	3.363398	-0.791841	0.978506
21	1	0	2.954769	-0.422170	1.830011
22	6	0	4.550802	-1.485865	0.837244
23	1	0	5.188566	-1.725006	1.673257
24	7	0	3.572017	-1.200455	-1.105213
25	1	0	3.351389	-1.222593	-2.090850
26	6	0	4.685983	-1.750832	-0.489713
27	1	0	5.460223	-2.266637	-1.034614
28	6	0	-2.859268	-0.481426	-0.190909
29	7	0	-3.566289	-1.152633	-1.133576
30	1	0	-3.228622	-1.221492	-2.083661
31	6	0	-4.729753	-1.701916	-0.618238
32	1	0	-5.420374	-2.275225	-1.215648
33	7	0	-3.616109	-0.606601	0.925993
34	1	0	-3.297926	-0.226344	1.808890

35	6	0	-4.761048	-1.351766	0.694319
36	1	0	-5.485779	-1.560526	1.465093
37	6	0	-1.332559	1.907788	-0.060054
38	5	0	-0.009936	-0.456089	0.461271

7

Standard orientation:

Center Number	Atomic Number	Atomic Type	Coordinates (Angstroms) X	Y	Z
1	6	0	0.604045	3.017993	-0.736870
2	5	0	-0.891745	2.929796	-0.870263
3	5	0	-0.035938	2.493544	0.799829
4	5	0	-1.469436	0.194487	-0.499658
5	5	0	1.488970	0.163687	-0.446721
6	5	0	-0.840003	0.821190	1.059320
7	5	0	0.902724	0.919386	1.042309
8	1	0	0.996798	4.025644	-0.892600
9	1	0	-2.543729	2.150154	0.386284
10	1	0	2.696892	2.468160	0.204549
11	1	0	-0.192914	3.209992	1.739688
12	1	0	1.312871	0.403667	-1.608273
13	1	0	-1.478636	0.610846	2.060319
14	1	0	1.542146	0.730449	2.045882
15	1	0	0.046094	-1.511585	0.952520
16	6	0	2.830622	-0.606537	-0.219660
17	7	0	3.414686	-0.963089	0.949693
18	1	0	3.000284	-0.692843	1.833003
19	6	0	4.593211	-1.663585	0.747187
20	1	0	5.201274	-2.027844	1.559689
21	7	0	3.670634	-1.102326	-1.164186
22	1	0	3.493814	-0.976454	-2.150183
23	6	0	4.757297	-1.755303	-0.597701
24	1	0	5.533699	-2.215480	-1.187494
25	6	0	-2.798473	-0.615995	-0.230128
26	7	0	-3.480888	-0.827553	0.919348
27	1	0	-3.140695	-0.454736	1.797999
28	6	0	-4.593270	-1.628907	0.718245
29	1	0	-5.260640	-1.909631	1.517257
30	7	0	-3.508542	-1.298265	-1.160017
31	1	0	-3.230535	-1.310079	-2.131330
32	6	0	-4.611762	-1.931251	-0.606163
33	1	0	-5.297440	-2.525388	-1.188557
34	5	0	0.068326	-0.470199	0.351632
35	6	0	-1.574624	1.831331	0.000323
36	5	0	1.609084	2.051109	-0.090907
37	1	0	-1.559100	3.847018	-1.267863
38	1	0	-1.193603	0.115262	-1.663864

TS7/8

Standard orientation:

Center Number	Atomic Number	Atomic Type	Coordinates (Angstroms) X	Y	Z
1	6	0	0.629510	3.204759	-0.801409
2	5	0	-0.923434	3.072157	-0.463306
3	5	0	0.487020	2.949785	0.804693
4	5	0	-1.507184	0.341427	-0.924271
5	5	0	1.313562	0.234956	-0.432161
6	5	0	-0.745346	1.577049	1.037879
7	5	0	0.971808	1.277149	0.972842
8	1	0	0.922992	4.192247	-1.159094
9	1	0	-2.768359	1.972506	-0.073888
10	1	0	2.864416	2.381477	-0.540744
11	1	0	0.615976	3.763338	1.669240
12	1	0	1.243666	0.533783	-1.592549
13	1	0	-1.412684	1.612323	2.039457
14	1	0	1.561501	1.029365	1.994679
15	1	0	-0.281145	-0.972827	1.239863
16	6	0	2.531925	-0.716455	-0.174040
17	7	0	2.972054	-1.204611	1.009872
18	1	0	2.537275	-0.918227	1.878283
19	6	0	4.056789	-2.048689	0.848484
20	1	0	4.549341	-2.530496	1.677886
21	7	0	3.363214	-1.278512	-1.086620
22	1	0	3.284295	-1.076362	-2.073222
23	6	0	4.307325	-2.098727	-0.486714
24	1	0	5.057758	-2.632704	-1.047257
25	6	0	-2.604918	-0.680178	-0.416780
26	7	0	-3.258165	-0.662726	0.767839
27	1	0	-3.014429	0.012388	1.485100
28	6	0	-4.147144	-1.716837	0.877280
29	1	0	-4.754526	-1.878131	1.753507
30	7	0	-3.104438	-1.764673	-1.053860
31	1	0	-2.802930	-2.022144	-1.983105
32	6	0	-4.056226	-2.418211	-0.284354
33	1	0	-4.573716	-3.301972	-0.621354
34	5	0	-0.079909	-0.013239	0.541014
35	6	0	-1.763717	1.795589	-0.482549
36	5	0	1.685630	2.198050	-0.421244
37	1	0	-1.523650	4.080650	-0.181778
38	1	0	-1.046427	0.014265	-1.982394

8

Standard orientation:

Center Number	Atomic Number	Atomic Type	Coordinates (Angstroms) X	Y	Z
1	6	0	1.404318	2.904380	-1.191852
2	5	0	0.362810	3.299392	-0.087798
3	5	0	2.106194	2.830895	0.325478
4	5	0	-0.934370	0.948106	-0.521505
5	5	0	1.025765	0.011104	-0.519499

6	5	0	0.851244	2.293080	1.379813
7	5	0	1.944209	1.061322	0.633885
8	1	0	1.712940	3.668558	-1.904794
9	1	0	-1.424994	2.394966	1.219714
10	1	0	3.183108	1.309171	-1.671263
11	1	0	3.021649	3.462497	0.763211
12	1	0	0.973991	0.103010	-1.711918
13	1	0	0.788974	2.554806	2.542832
14	1	0	2.736650	0.469566	1.313867
15	1	0	-0.190740	-0.029275	1.761173
16	6	0	1.446497	-1.478107	-0.206238
17	7	0	1.638003	-2.093610	0.983661
18	1	0	1.566900	-1.573930	1.849515
19	6	0	2.050965	-3.404603	0.824668
20	1	0	2.266036	-4.053177	1.658914
21	7	0	1.729033	-2.439389	-1.119111
22	1	0	1.694394	-2.251127	-2.111517
23	6	0	2.110055	-3.627942	-0.515212
24	1	0	2.384638	-4.506751	-1.076209
25	6	0	-2.239114	0.101163	-0.233442
26	7	0	-2.740670	-0.386492	0.925607
27	1	0	-2.224823	-0.292878	1.792215
28	6	0	-3.953622	-1.026163	0.732097
29	1	0	-4.508903	-1.479341	1.537798
30	7	0	-3.163824	-0.245937	-1.160578
31	1	0	-3.062610	0.024054	-2.129653
32	6	0	-4.226135	-0.934798	-0.596869
33	1	0	-5.064909	-1.289644	-1.174019
34	5	0	0.201439	0.726970	0.900411
35	6	0	-0.616496	2.116784	0.544971
36	5	0	2.233079	1.650134	-1.028388
37	1	0	-0.015233	4.428514	0.061626
38	1	0	-0.885524	1.209691	-1.689266

TS8/m-2

Standard orientation:

Center Number	Atomic Number	Atomic Type	Coordinates (Angstroms) X	Y	Z
1	6	0	-0.031821	2.308113	1.517644
2	5	0	0.989832	2.323826	0.362283
3	5	0	-0.705535	2.979140	0.164579
4	5	0	1.637749	-0.016632	0.321746
5	5	0	-1.500347	0.028996	0.333583
6	5	0	-0.020225	2.019376	-1.171277
7	5	0	-1.601179	1.615575	-0.475932
8	1	0	0.178784	2.918493	2.395369
9	1	0	1.788180	0.988737	-1.586834
10	1	0	-2.384502	1.977189	2.001161
11	1	0	-0.985281	4.116810	-0.070614
12	1	0	-0.965334	-0.405409	1.315874
13	1	0	0.193249	2.517989	-2.234532

14	1	0	-2.606577	1.798413	-1.113816
15	1	0	-0.599698	-0.288706	-2.117109
16	6	0	-2.837909	-0.742046	0.103534
17	7	0	-3.790392	-0.569560	-0.848899
18	1	0	-3.723760	0.199249	-1.503686
19	6	0	-4.818117	-1.491485	-0.730210
20	1	0	-5.665847	-1.507043	-1.396491
21	7	0	-3.298631	-1.801920	0.822817
22	1	0	-2.793394	-2.154571	1.621946
23	6	0	-4.507799	-2.277338	0.332464
24	1	0	-5.029877	-3.111131	0.773861
25	6	0	3.073447	-0.637838	0.087956
26	7	0	3.920121	-0.518470	-0.961422
27	1	0	3.682143	0.022382	-1.783164
28	6	0	5.104760	-1.192431	-0.740613
29	1	0	5.906522	-1.214067	-1.461417
30	7	0	3.751806	-1.406668	0.972111
31	1	0	3.358720	-1.654169	1.871016
32	6	0	4.999124	-1.758615	0.494164
33	1	0	5.691476	-2.365649	1.055184
34	5	0	-0.504036	0.285744	-1.067884
35	6	0	1.106371	0.892720	-0.735032
36	5	0	-1.457331	1.766486	1.270342
37	1	0	1.987759	2.985194	0.319918
38	1	0	1.164145	-0.366425	1.354444

TSm-2/9

Standard orientation:

Center Number	Atomic Number	Atomic Type	Coordinates (Angstroms) X	Y	Z
1	6	0	-0.798602	-0.450140	1.145088
2	5	0	-1.566445	-0.618751	-0.169319
3	5	0	-0.551173	1.332783	1.033576
4	5	0	0.672431	-0.093426	1.353162
5	5	0	1.191053	1.351006	-1.258311
6	1	0	-1.369489	-0.612670	2.069365
7	1	0	-2.503119	1.884629	-0.744523
8	1	0	-1.163735	-1.190577	-1.142270
9	1	0	-1.012106	1.904404	1.982397
10	1	0	1.152527	-0.190285	2.451613
11	1	0	-0.520557	1.904873	-2.663972
12	1	0	-0.022055	3.547124	-0.111149
13	1	0	1.097710	-1.008481	-0.850173
14	1	0	2.133337	1.921454	1.108979
15	1	0	2.067377	1.823553	-1.931029
16	6	0	-3.139794	-0.666664	-0.052755
17	7	0	-4.016787	-1.243239	-0.906090
18	7	0	-3.920963	-0.133507	0.914170
19	1	0	-3.710106	-1.718024	-1.743923
20	1	0	-3.525844	0.426879	1.659399
21	6	0	-5.263576	-0.355329	0.666781

22	6	0	-5.327237	-1.066733	-0.490918
23	1	0	-6.043529	0.003917	1.318871
24	1	0	-6.171131	-1.452995	-1.039516
25	5	0	-1.389122	1.458831	-0.632580
26	5	0	1.647339	-0.145436	-0.229046
27	5	0	1.235777	1.372692	0.520025
28	6	0	-0.293115	1.474148	-1.686468
29	5	0	0.032087	2.364026	-0.263010
30	6	0	3.176737	-0.455668	-0.092251
31	7	0	3.828803	-1.550000	-0.561616
32	1	0	3.344353	-2.278449	-1.065848
33	6	0	5.181562	-1.515300	-0.256032
34	1	0	5.865953	-2.296385	-0.545810
35	7	0	4.150809	0.265915	0.510851
36	1	0	3.929586	1.149309	0.955252
37	6	0	5.383598	-0.358613	0.427884
38	1	0	6.280626	0.064710	0.850679

9

Standard orientation:

Center Number	Atomic Number	Atomic Type	Coordinates (Angstroms) X	Y	Z
1	6	0	-0.758251	-1.014730	0.763431
2	5	0	-1.618634	-0.353177	-0.367084
3	5	0	-0.879307	0.710431	0.949756
4	5	0	0.731400	-0.892554	0.914355
5	5	0	1.339028	1.775361	-0.641822
6	1	0	-1.299113	-1.564028	1.539230
7	1	0	-2.396210	2.077487	-0.810430
8	1	0	-1.275825	-0.519256	-1.507951
9	1	0	-1.583316	0.935621	1.903278
10	1	0	1.300579	-1.449171	1.817853
11	1	0	-0.134364	3.078667	-1.783219
12	1	0	-0.167231	3.170037	1.190192
13	1	0	1.228928	-0.489364	-1.386213
14	1	0	1.732851	1.099395	1.887854
15	1	0	2.306141	2.467064	-0.815315
16	6	0	-3.183024	-0.506810	-0.186350
17	7	0	-4.081543	-0.880074	-1.123318
18	7	0	-3.927528	-0.289658	0.919057
19	1	0	-3.804229	-1.097035	-2.070467
20	1	0	-3.505274	0.074028	1.765486
21	6	0	-5.274966	-0.510065	0.681078
22	6	0	-5.375134	-0.888888	-0.619703
23	1	0	-6.029192	-0.380412	1.440524
24	1	0	-6.231433	-1.158514	-1.216535
25	5	0	-1.320213	1.571102	-0.637457
26	5	0	1.682277	-0.066202	-0.358862
27	5	0	1.067312	0.981514	0.891836
28	6	0	-0.051434	2.153261	-1.211011
29	5	0	-0.022299	2.217800	0.484550

30	6	0	3.206877	-0.382228	-0.204244
31	7	0	3.989433	-1.103698	-1.045043
32	1	0	3.622494	-1.514057	-1.891431
33	6	0	5.297533	-1.188248	-0.586813
34	1	0	6.069240	-1.718722	-1.120963
35	7	0	4.051656	-0.010891	0.786517
36	1	0	3.724341	0.552966	1.560869
37	6	0	5.334050	-0.494594	0.579867
38	1	0	6.145763	-0.302020	1.262848

TS9/p-2

Standard orientation:

Center Number	Atomic Number	Atomic Type	Coordinates (Angstroms) X	Y	Z
1	6	0	0.234267	1.991443	1.335153
2	5	0	1.624990	-0.507210	0.416262
3	5	0	0.501885	1.094911	-1.196797
4	5	0	1.339435	1.564934	0.354687
5	5	0	-0.051665	2.460163	-0.265050
6	5	0	0.730416	-0.850567	-0.916108
7	5	0	-1.608121	-0.074272	0.282119
8	5	0	-1.270050	1.319030	-0.762148
9	5	0	-1.224993	1.748809	0.948499
10	6	0	-0.692600	-0.227101	-1.107061
11	1	0	0.460421	2.716728	2.118683
12	1	0	1.305815	-0.644877	1.564689
13	1	0	0.971863	1.253181	-2.284067
14	1	0	2.439397	2.047559	0.345439
15	1	0	0.008068	3.560398	-0.724387
16	1	0	1.184044	-1.371852	-1.918086
17	1	0	-1.140373	-0.614663	1.241472
18	1	0	-2.166309	1.645553	-1.496728
19	1	0	-2.157440	2.310349	1.454424
20	1	0	-1.168879	-0.504613	-2.047880
21	6	0	-3.135162	-0.461428	0.158641
22	7	0	-4.087646	-0.033876	-0.700883
23	1	0	-3.879532	0.686502	-1.382695
24	6	0	-5.311738	-0.630885	-0.451257
25	1	0	-6.195067	-0.412251	-1.029672
26	7	0	-3.785077	-1.344490	0.952946
27	1	0	-3.309232	-1.829404	1.701195
28	6	0	-5.120903	-1.466842	0.603523
29	1	0	-5.803095	-2.120522	1.122840
30	6	0	3.178521	-0.627998	0.225027
31	7	0	4.150323	-0.705117	1.168389
32	1	0	3.936160	-0.654678	2.154203
33	6	0	5.412362	-0.802481	0.602766
34	1	0	6.313644	-0.868634	1.190860
35	7	0	3.859551	-0.684447	-0.944619
36	1	0	3.366823	-0.667837	-1.829089
37	6	0	5.224022	-0.789921	-0.744014

38	1	0	5.931524	-0.843137	-1.556010

m

Standard orientation:

Center Number	Atomic Number	Atomic Type	Coordinates (Angstroms) X	Y	Z
1	6	0	-0.874604	-1.421907	-0.000000
2	5	0	0.371909	-1.098558	0.937529
3	5	0	-1.191715	-0.141188	0.921205
4	5	0	0.371909	0.648610	-1.304886
5	5	0	0.371909	0.648610	1.304886
6	5	0	-0.711596	1.357949	-0.000000
7	1	0	-1.423675	-2.352577	-0.000000
8	1	0	0.863829	-1.890077	-1.666728
9	1	0	-2.095431	-0.161785	-1.687437
10	1	0	-2.095431	-0.161785	1.687437
11	1	0	0.753318	0.945673	-2.388306
12	1	0	0.753318	0.945673	2.388306
13	1	0	-1.431825	2.302250	-0.000000
14	1	0	1.733976	2.464607	0.000000
15	5	0	0.969350	1.562569	0.000000
16	5	0	-1.191715	-0.141188	-0.921205
17	1	0	0.863829	-1.890077	1.666728
18	1	0	2.412133	-0.245375	0.000000
19	6	0	1.350629	-0.019386	0.000000
20	5	0	0.371909	-1.098558	-0.937529

TSm/10

Standard orientation:

Center Number	Atomic Number	Atomic Type	Coordinates (Angstroms) X	Y	Z
1	6	0	0.982393	0.024771	-1.443713
2	5	0	0.377609	-0.748316	-0.202320
3	5	0	2.321015	0.909137	-1.119602
4	5	0	0.768852	1.037216	-0.194810
5	5	0	2.820711	-0.090230	1.540395
6	1	0	0.505542	0.089367	-2.412303
7	1	0	2.635301	-1.723233	-1.875431
8	1	0	-0.125274	-1.822073	-0.173247
9	1	0	2.717648	1.656220	-1.952211
10	1	0	-0.033470	1.911743	-0.216837
11	1	0	2.379651	-2.224338	0.820084
12	1	0	4.570798	-0.180180	-0.239437
13	1	0	0.495188	-0.142836	2.237748
14	1	0	2.565214	2.374304	1.069090
15	1	0	3.360701	-0.264010	2.579973
16	6	0	-1.960118	-0.100217	-0.116966
17	7	0	-2.898368	-1.081362	-0.040642
18	7	0	-2.719816	1.017334	0.031721

19	1	0	-2.651528	-2.058770	-0.106631
20	1	0	-2.306758	1.939319	0.033767
21	6	0	-4.074297	0.750171	0.196692
22	6	0	-4.189806	-0.601796	0.149752
23	1	0	-4.818563	1.520139	0.329679
24	1	0	-5.054272	-1.242209	0.232911
25	5	0	2.248046	-0.906091	-1.105884
26	5	0	1.124153	-0.013797	1.235702
27	5	0	2.326137	1.280562	0.666394
28	6	0	2.261809	-1.181555	0.540295
29	5	0	3.406922	-0.015179	-0.064087

10

Standard orientation:

Center Number	Atomic Number	Atomic Type	Coordinates (Angstroms) X	Y	Z
1	6	0	-0.713011	0.556780	1.233918
2	5	0	0.197183	-0.464216	0.327774
3	5	0	-2.092865	1.310552	0.630356
4	5	0	-0.669043	0.966397	-0.364995
5	5	0	-2.857797	-0.730769	-1.238623
6	1	0	-0.264589	1.151976	2.024731
7	1	0	-2.425400	-0.653721	2.500020
8	1	0	0.163750	-1.627487	0.633383
9	1	0	-2.347960	2.385068	1.071498
10	1	0	0.077978	1.788653	-0.806649
11	1	0	-2.539541	-2.319357	0.337141
12	1	0	-4.472268	0.193425	0.446128
13	1	0	-0.631559	-1.211265	-1.998725
14	1	0	-2.496123	1.671549	-1.966715
15	1	0	-3.465085	-1.310188	-2.077057
16	6	0	1.761270	-0.163083	0.083412
17	7	0	2.718671	-1.104942	0.232260
18	7	0	2.448324	0.950476	-0.248360
19	1	0	2.485937	-2.054752	0.491950
20	1	0	1.968623	1.816932	-0.461053
21	6	0	3.809929	0.714977	-0.313926
22	6	0	3.983781	-0.599199	-0.007462
23	1	0	4.522433	1.483273	-0.568640
24	1	0	4.876392	-1.200342	0.058692
25	5	0	-2.051316	-0.333454	1.413226
26	5	0	-1.110238	-0.620344	-1.077302
27	5	0	-2.262037	0.855097	-1.130886
28	6	0	-2.315016	-1.278239	0.112253
29	5	0	-3.310540	0.132754	0.196216

TS10/11

Standard orientation:

Center Number	Atomic Number	Atomic Type	Coordinates (Angstroms) X	Y	Z

1	6	0	-2.107161	0.950345	-0.944610
2	5	0	0.462405	0.440131	0.255935
3	5	0	-2.926187	-0.529395	-1.057773
4	5	0	-1.115014	-0.305905	-1.110606
5	5	0	-2.367601	1.139698	0.811905
6	5	0	-3.275357	-0.432393	0.732949
7	5	0	-1.910908	-1.437703	0.128104
8	5	0	-1.597879	-0.247478	1.469418
9	1	0	-2.017349	1.719801	-1.709471
10	1	0	-4.536933	1.494591	-0.650164
11	1	0	0.217575	1.599638	0.318081
12	1	0	-3.401089	-1.069354	-2.006531
13	1	0	-0.564924	-0.615832	-2.126109
14	1	0	-2.454086	2.181663	1.393427
15	1	0	-4.114181	-0.959039	1.399111
16	1	0	0.160717	-1.700975	0.445781
17	1	0	-1.893907	-2.628018	0.149435
18	1	0	-1.400123	-0.536144	2.610915
19	6	0	2.025730	0.186124	0.114017
20	7	0	2.970483	1.152209	0.133581
21	7	0	2.721197	-0.954573	-0.093102
22	1	0	2.734246	2.127189	0.271330
23	1	0	2.271548	-1.857868	-0.174270
24	6	0	4.075987	-0.711407	-0.202537
25	6	0	4.235788	0.634172	-0.054903
26	1	0	4.797165	-1.494723	-0.373458
27	1	0	5.122947	1.246878	-0.068867
28	5	0	-3.592425	0.858892	-0.311769
29	6	0	-0.421765	-0.779317	0.362470

11

Standard orientation:

Center Number	Atomic Number	Atomic Type	Coordinates (Angstroms) X	Y	Z
1	6	0	1.729392	-0.451912	-1.254851
2	5	0	-0.370389	-0.654577	0.268142
3	5	0	2.518074	0.984911	-0.923039
4	5	0	0.775924	0.686004	-0.582557
5	5	0	2.462771	-1.351108	0.172595
6	5	0	3.332297	0.211328	0.534140
7	5	0	1.897444	1.227790	0.776336
8	5	0	1.910971	-0.431444	1.499187
9	1	0	1.406577	-0.835260	-2.219886
10	1	0	4.131804	-0.877332	-1.810570
11	1	0	-0.190174	-1.819125	0.100191
12	1	0	2.728863	1.906747	-1.644955
13	1	0	0.045947	1.409258	-1.204072
14	1	0	2.682976	-2.525823	0.199804
15	1	0	4.363032	0.484441	1.069933
16	1	0	-0.072749	1.046227	1.650819

17	1	0	1.892303	2.300343	1.292465
18	1	0	2.045190	-0.659028	2.664776
19	6	0	-1.907303	-0.267229	0.066060
20	7	0	-2.941334	-1.135261	0.051862
21	7	0	-2.482118	0.942044	-0.102104
22	1	0	-2.806533	-2.132485	0.162133
23	1	0	-1.925582	1.783707	-0.198908
24	6	0	-3.854526	0.840413	-0.221821
25	6	0	-4.149885	-0.485586	-0.117479
26	1	0	-4.491775	1.696931	-0.373840
27	1	0	-5.093523	-1.006062	-0.151817
28	5	0	3.330224	-0.534630	-1.003650
29	6	0	0.509191	0.345749	1.046399

TS11/12

Standard orientation:

Center Number	Atomic Number	Atomic Type	Coordinates (Angstroms) X	Y	Z
1	6	0	-1.575308	0.415146	-1.258376
2	5	0	0.319544	0.620788	0.307007
3	5	0	-2.461715	-0.970463	-0.956294
4	5	0	-0.747141	-0.770163	-0.500820
5	5	0	-2.401156	1.377334	0.165938
6	5	0	-3.334941	-0.158961	0.438230
7	5	0	-1.963032	-1.228842	0.779356
8	5	0	-1.966206	0.418576	1.506128
9	1	0	-1.177446	0.763786	-2.208094
10	1	0	-3.895961	0.980990	-1.959791
11	1	0	0.178124	1.801810	0.222303
12	1	0	-2.676502	-1.876801	-1.696250
13	1	0	-0.006697	-1.526988	-1.064320
14	1	0	-2.548698	2.562742	0.168918
15	1	0	-4.418395	-0.378485	0.886758
16	1	0	-0.006576	-1.051243	1.773661
17	1	0	-2.023639	-2.297213	1.299818
18	1	0	-2.178597	0.632314	2.662672
19	6	0	1.857905	0.243319	0.063086
20	7	0	2.873076	1.133789	0.073858
21	7	0	2.461270	-0.948916	-0.124263
22	1	0	2.712621	2.124542	0.206928
23	1	0	1.922363	-1.800164	-0.235916
24	6	0	3.831923	-0.814944	-0.230078
25	6	0	4.096921	0.515200	-0.098800
26	1	0	4.489269	-1.654146	-0.392960
27	1	0	5.028875	1.057144	-0.117204
28	5	0	-3.175099	0.600776	-1.094938
29	6	0	-0.559845	-0.371658	1.121379

12

Standard orientation:

Center Number	Atomic Number	Atomic Type	Coordinates (Angstroms) X	Y	Z
1	6	0	0.848917	-0.097197	1.288460
2	5	0	-0.090689	0.514089	0.000000
3	5	0	2.258964	-0.989353	0.906584
4	5	0	0.786329	-1.155555	0.000000
5	5	0	2.230499	1.662623	0.000000
6	5	0	3.197481	0.208622	0.000000
7	5	0	2.258964	-0.989353	-0.906584
8	5	0	2.258964	0.704459	-1.433490
9	1	0	0.338292	-0.382012	2.205918
10	1	0	2.703400	0.982158	2.504372
11	1	0	-0.132137	1.725461	0.000000
12	1	0	2.618213	-1.887503	1.597158
13	1	0	0.073063	-2.112978	0.000000
14	1	0	1.930231	2.809592	0.000000
15	1	0	4.379212	0.382413	0.000000
16	1	0	0.338292	-0.382012	-2.205918
17	1	0	2.618213	-1.887503	-1.597158
18	1	0	2.703400	0.982158	-2.504372
19	6	0	-1.653399	0.114074	0.000000
20	7	0	-2.632554	1.045135	0.000000
21	7	0	-2.321839	-1.060437	0.000000
22	1	0	-2.413554	2.033198	0.000000
23	1	0	-1.831453	-1.946359	0.000000
24	6	0	-3.693505	-0.872021	0.000000
25	6	0	-3.892419	0.473358	0.000000
26	1	0	-4.393656	-1.692170	0.000000
27	1	0	-4.799211	1.056619	0.000000
28	5	0	2.258964	0.704459	1.433490
29	6	0	0.848917	-0.097197	-1.288460

TS12/m-2

Standard orientation:

Center Number	Atomic Number	Atomic Type	Coordinates (Angstroms) X	Y	Z
1	6	0	1.219963	0.080535	1.284018
2	5	0	1.235027	-1.029945	0.000000
3	5	0	1.219963	1.745313	0.904522
4	5	0	2.157307	0.609490	0.000000
5	5	0	-1.162864	0.395509	0.000000
6	5	0	-0.287503	1.911757	0.000000
7	5	0	1.219963	1.745313	-0.904522
8	5	0	-0.221320	0.835030	-1.426151
9	1	0	1.743004	-0.179806	2.202388
10	1	0	-0.656282	1.088256	2.511836
11	1	0	0.254737	-1.745746	0.000000
12	1	0	1.781107	2.537320	1.594380
13	1	0	3.352718	0.552583	0.000000
14	1	0	-1.483020	-0.748869	0.000000

15	1	0	-0.938151	2.919708	0.000000
16	1	0	1.743004	-0.179806	-2.202388
17	1	0	1.781107	2.537320	-1.594380
18	1	0	-0.656282	1.088256	-2.511836
19	6	0	2.444054	-2.103497	0.000000
20	7	0	2.215737	-3.436258	0.000000
21	7	0	3.792959	-2.008425	0.000000
22	1	0	1.270362	-3.796744	0.000000
23	1	0	4.251052	-1.105057	0.000000
24	6	0	4.399027	-3.253397	0.000000
25	6	0	3.391289	-4.166612	0.000000
26	1	0	5.469971	-3.380210	0.000000
27	1	0	3.411101	-5.244647	0.000000
28	5	0	-0.221320	0.835030	1.426151
29	6	0	1.219963	0.080535	-1.284018
30	6	0	-3.286988	1.277610	0.000000
31	7	0	-3.609062	2.587616	0.000000
32	6	0	-4.980569	2.803978	0.000000
33	1	0	-5.421937	3.788296	0.000000
34	7	0	-4.495110	0.665923	0.000000
35	1	0	-4.588265	-0.338999	0.000000
36	6	0	-5.551925	1.571628	0.000000
37	1	0	-6.587635	1.269771	0.000000
38	1	0	-2.883424	3.294201	0.000000

----

m-2

Standard orientation:

Center Number	Atomic Number	Atomic Type	Coordinates (Angstroms) X	Y	Z
1	6	0	0.724849	0.874314	1.280872
2	5	0	-0.245383	1.403745	0.000000
3	5	0	2.204564	0.107302	0.898750
4	5	0	0.724849	-0.751606	1.413950
5	5	0	0.724849	-0.751606	-1.413950
6	1	0	0.740309	1.455227	2.201240
7	1	0	2.146635	2.547438	0.000000
8	1	0	-1.339848	0.869420	0.000000
9	1	0	3.162782	0.232769	1.595061
10	1	0	0.748807	-1.249042	2.504332
11	1	0	0.740309	1.455227	-2.201240
12	1	0	3.162782	0.232769	-1.595061
13	1	0	-1.112151	-0.925550	0.000000
14	1	0	2.302343	-2.326168	0.000000
15	1	0	0.748807	-1.249042	-2.504332
16	6	0	-0.631667	2.975412	0.000000
17	7	0	-1.916646	3.397144	0.000000
18	7	0	0.082751	4.123600	0.000000
19	1	0	-2.676890	2.729801	0.000000
20	1	0	1.095631	4.104385	0.000000
21	6	0	-0.734150	5.241485	0.000000
22	6	0	-2.012671	4.777993	0.000000

23	1	0	-0.345471	6.247452	0.000000
24	1	0	-2.956232	5.299791	0.000000
25	5	0	1.634596	1.464817	0.000000
26	5	0	-0.086774	-1.549754	0.000000
27	5	0	1.661189	-1.307327	0.000000
28	6	0	0.724849	0.874314	-1.280872
29	5	0	2.204564	0.107302	-0.898750
30	6	0	-0.415792	-3.077144	0.000000
31	7	0	-1.659499	-3.627680	0.000000
32	1	0	-2.489542	-3.053598	0.000000
33	6	0	-1.613854	-5.014927	0.000000
34	1	0	-2.500618	-5.628263	0.000000
35	7	0	0.407303	-4.155052	0.000000
36	1	0	1.412057	-4.031356	0.000000
37	6	0	-0.297747	-5.348230	0.000000
38	1	0	0.187761	-6.311009	0.000000

TS5/14

Standard orientation:

Center Number	Atomic Number	Atomic Type	Coordinates (Angstroms) X	Y	Z
1	5	0	-1.180602	0.316316	-0.834447
2	5	0	-0.885612	0.564360	0.927745
3	5	0	1.667745	2.007228	0.629647
4	5	0	0.844234	0.533866	1.283865
5	5	0	1.823528	0.400801	-0.156401
6	5	0	0.450083	2.514331	-0.698731
7	5	0	-0.033330	2.095305	1.059078
8	6	0	1.717837	1.681234	-0.988359
9	1	0	0.141027	-1.776028	0.251641
10	1	0	-1.611066	-0.003619	-1.898139
11	1	0	-1.710490	0.304279	1.747068
12	1	0	2.498567	2.567437	1.281515
13	1	0	1.171895	0.042289	2.323786
14	1	0	-1.878700	2.414498	-0.306029
15	1	0	0.202420	-0.344897	-1.140217
16	1	0	0.325018	3.648151	-1.062393
17	1	0	-0.421969	2.848200	1.897918
18	1	0	2.520402	2.068882	-1.615027
19	6	0	3.104310	-0.490161	-0.108508
20	7	0	3.175510	-1.790061	0.249418
21	1	0	2.343756	-2.299190	0.524928
22	6	0	4.474513	-2.266864	0.180798
23	1	0	4.735640	-3.283398	0.427040
24	7	0	4.378307	-0.153996	-0.413925
25	1	0	4.630615	0.782758	-0.698701
26	6	0	5.241125	-1.225165	-0.237545
27	1	0	6.301071	-1.154696	-0.421707
28	6	0	-3.485130	-0.323796	-0.248869
29	7	0	-4.383517	-0.747737	-1.178682
30	1	0	-4.171757	-0.729744	-2.166112

31	6	0	-5.581463	-1.195015	-0.630035
32	1	0	-6.405237	-1.564609	-1.221394
33	7	0	-4.170808	-0.525928	0.906281
34	1	0	-3.752376	-0.308256	1.800554
35	6	0	-5.443002	-1.051963	0.713564
36	1	0	-6.123132	-1.273530	1.521799
37	6	0	-1.006689	1.767519	-0.266212
38	5	0	0.129730	-0.590048	0.053807

14

Standard orientation:

Center Number	Atomic Number	Atomic Type	Coordinates (Angstroms) X	Y	Z
1	5	0	2.493645	1.192770	-1.145912
2	5	0	2.421535	1.215242	0.596808
3	5	0	0.887474	-1.295547	0.870249
4	5	0	0.901138	0.467858	1.145620
5	5	0	-0.057530	-0.181378	-0.158449
6	5	0	2.156725	-1.465834	-0.492020
7	5	0	2.442972	-0.500094	1.069588
8	6	0	0.655670	-1.378839	-0.787654
9	1	0	0.340078	2.497728	-0.472595
10	1	0	3.047301	1.906741	-1.918139
11	1	0	3.056144	2.007221	1.216044
12	1	0	0.445122	-2.050334	1.681744
13	1	0	0.375019	0.959907	2.099150
14	1	0	4.164228	-0.158189	-0.425875
15	1	0	1.219829	1.025726	-1.544609
16	1	0	2.814543	-2.444552	-0.681420
17	1	0	3.169787	-0.817665	1.957348
18	1	0	0.129774	-2.214553	-1.249284
19	6	0	-1.613787	-0.038351	-0.092457
20	7	0	-2.321139	1.100287	0.065020
21	1	0	-1.861504	1.998883	0.148778
22	6	0	-3.684371	0.854616	0.086962
23	1	0	-4.415149	1.638585	0.203184
24	7	0	-2.551227	-1.008353	-0.177680
25	1	0	-2.307930	-1.984844	-0.276818
26	6	0	-3.831420	-0.488126	-0.064305
27	1	0	-4.714961	-1.104829	-0.100886
28	6	0	3.087199	-0.019349	-0.387483
29	5	0	0.946949	1.470368	-0.361559

TS14/15

Standard orientation:

Center Number	Atomic Number	Atomic Type	Coordinates (Angstroms) X	Y	Z
1	5	0	-2.341268	-1.262887	-1.093986
2	5	0	-2.385674	-1.203028	0.708568

3	5	0	-0.925743	1.345401	0.719913
4	5	0	-0.924285	-0.427222	1.216470
5	5	0	0.040577	0.116482	-0.145769
6	5	0	-2.257038	1.344661	-0.621258
7	5	0	-2.465815	0.517281	1.051717
8	6	0	-0.733756	1.362128	-0.881193
9	1	0	-0.189415	-2.450521	-0.287241
10	1	0	-2.866885	-1.935926	-1.927025
11	1	0	-3.015523	-1.996034	1.333960
12	1	0	-0.442268	2.163214	1.443772
13	1	0	-0.391045	-0.716128	2.244225
14	1	0	-4.169668	0.007280	-0.459749
15	1	0	-0.584681	-0.055537	-1.266512
16	1	0	-2.935440	2.288381	-0.888751
17	1	0	-3.192878	0.913392	1.906528
18	1	0	-0.180034	2.126345	-1.416268
19	6	0	1.605868	0.036441	-0.102981
20	7	0	2.327339	-1.099053	-0.107411
21	1	0	1.865599	-2.002513	-0.159354
22	6	0	3.681402	-0.837329	-0.003645
23	1	0	4.425898	-1.616911	0.010610
24	7	0	2.517421	1.026168	-0.005128
25	1	0	2.254929	2.001193	0.060066
26	6	0	3.804244	0.516345	0.063271
27	1	0	4.675021	1.146065	0.150127
28	6	0	-3.092439	-0.104647	-0.372050
29	5	0	-0.892526	-1.470635	-0.271061

15

Standard orientation:

Center Number	Atomic Number	Atomic Type	Coordinates (Angstroms) X	Y	Z
1	5	0	-2.085711	-0.779042	-1.410248
2	5	0	-2.335881	-1.419550	0.247022
3	5	0	-1.002533	0.981641	1.022876
4	5	0	-0.913611	-0.793200	1.079871
5	5	0	0.001053	0.106625	-0.185180
6	5	0	-2.347282	1.418163	-0.041452
7	5	0	-2.500516	0.099986	1.157392
8	6	0	-0.731867	1.543885	-0.592427
9	1	0	-0.128326	-2.375895	-0.843319
10	1	0	-2.349760	-0.904601	-2.566742
11	1	0	-3.012541	-2.370255	0.483160
12	1	0	-0.532684	1.743097	1.806192
13	1	0	-0.403039	-1.294866	2.036327
14	1	0	-4.117861	0.024896	-0.593481
15	1	0	-0.972502	1.446872	-1.646056
16	1	0	-3.009204	2.406276	0.009948
17	1	0	-3.237224	0.176303	2.087322
18	1	0	-0.240020	2.506771	-0.435366
19	6	0	1.564056	0.051422	-0.092419

20	7	0	2.299137	-1.067925	0.075444
21	1	0	1.849979	-1.975527	0.128550
22	6	0	3.652890	-0.789509	0.114660
23	1	0	4.401455	-1.554903	0.241933
24	7	0	2.479447	1.044876	-0.170008
25	1	0	2.219087	2.014343	-0.285726
26	6	0	3.771177	0.556479	-0.038840
27	1	0	4.640294	1.193633	-0.069588
28	6	0	-3.045782	-0.018006	-0.425209
29	5	0	-0.779635	-1.402708	-0.567442

TS6/15

Standard orientation:

Center Number	Atomic Number	Atomic Type	Coordinates (Angstroms) X	Y	Z
1	5	0	-0.853177	0.955565	-0.742677
2	5	0	-0.823372	1.160421	1.056076
3	5	0	1.866853	1.987705	0.578350
4	5	0	0.860858	0.787939	1.424031
5	5	0	1.598553	0.372324	-0.164111
6	5	0	0.794665	2.931805	-0.462161
7	5	0	0.328680	2.508309	1.208613
8	6	0	1.794571	1.705102	-1.135283
9	1	0	-0.242440	-1.329474	0.378228
10	1	0	-1.329161	0.779198	-1.816675
11	1	0	-1.781019	1.079212	1.752954
12	1	0	2.949896	2.362071	0.897534
13	1	0	1.255791	0.356644	2.468836
14	1	0	-1.501366	3.130408	-0.200074
15	1	0	1.166823	1.535034	-2.006480
16	1	0	0.964956	4.056781	-0.814915
17	1	0	0.196662	3.326268	2.061617
18	1	0	2.784118	2.025868	-1.470949
19	6	0	2.713921	-0.721072	-0.141052
20	7	0	2.762868	-1.796089	0.676645
21	1	0	2.042321	-1.954472	1.370128
22	6	0	3.879564	-2.575543	0.430895
23	1	0	4.094399	-3.475758	0.983970
24	7	0	3.817406	-0.841918	-0.915705
25	1	0	4.049332	-0.167242	-1.631128
26	6	0	4.553575	-1.970398	-0.582376
27	1	0	5.468120	-2.238922	-1.086045
28	6	0	-3.677051	0.011240	-0.350162
29	7	0	-5.025812	-0.156116	-0.515154
30	1	0	-5.620427	0.594612	-0.831360
31	6	0	-5.481722	-1.434932	-0.201328
32	1	0	-6.516694	-1.733211	-0.273465
33	7	0	-3.319237	-1.233730	0.079732
34	1	0	-2.353250	-1.443625	0.298841
35	6	0	-4.378865	-2.127214	0.181794
36	1	0	-4.265862	-3.149651	0.509215

37	6	0	-0.706908	2.396087	-0.111932
38	5	0	-0.011317	-0.155743	0.219440

TS15/16

Standard orientation:

Center Number	Atomic Number	Atomic Type	Coordinates (Angstroms) X	Y	Z
1	5	0	-0.885819	-1.331196	-0.190146
2	5	0	-2.395704	-1.271883	0.737349
3	5	0	-1.897051	1.627107	-0.422952
4	5	0	-2.131583	0.549006	1.266264
5	5	0	-0.069224	0.337615	0.095098
6	5	0	-1.507291	-0.005710	-1.393880
7	5	0	-2.963368	0.262161	-0.324890
8	6	0	-0.419299	1.902227	0.122018
9	1	0	-0.359462	-1.044481	2.348152
10	1	0	-0.219725	-2.266007	-0.534365
11	1	0	-3.112320	-2.046076	1.286602
12	1	0	-2.551922	2.515363	-0.890966
13	1	0	-2.497161	1.281716	2.130729
14	1	0	-2.913030	-1.879810	-1.468961
15	1	0	0.054844	2.543232	-0.645833
16	1	0	-1.318669	0.118794	-2.563812
17	1	0	-4.079468	0.515542	-0.649245
18	1	0	-0.332052	2.419816	1.085561
19	6	0	1.497680	0.028465	-0.003489
20	7	0	2.195906	-1.128680	-0.046020
21	1	0	1.724880	-2.025291	-0.048586
22	6	0	3.558144	-0.902415	-0.083246
23	1	0	4.281729	-1.701325	-0.118407
24	7	0	2.449686	0.987798	-0.014375
25	1	0	2.198418	1.967846	0.019782
26	6	0	3.721688	0.449785	-0.064388
27	1	0	4.613723	1.055288	-0.079533
28	6	0	-2.367602	-1.181452	-0.847228
29	5	0	-0.940478	-0.616718	1.395085

16

Standard orientation:

Center Number	Atomic Number	Atomic Type	Coordinates (Angstroms) X	Y	Z
1	5	0	-0.986511	-1.264371	0.607743
2	5	0	-2.728816	-0.890150	0.697162
3	5	0	-1.363780	1.266227	-0.902330
4	5	0	-2.610241	0.901663	0.602973
5	5	0	-0.107594	0.396490	0.165178
6	5	0	-0.937609	-0.508558	-1.207434
7	5	0	-2.786271	0.038766	-0.858307
8	6	0	-0.401069	2.002075	0.126544

9	1	0	-1.178706	0.175102	2.687687
10	1	0	-0.317218	-2.212752	0.905232
11	1	0	-3.596740	-1.495363	1.238895
12	1	0	-1.311436	1.698935	-2.022550
13	1	0	-3.355573	1.776053	0.918611
14	1	0	-2.318156	-2.351315	-1.141166
15	1	0	0.333220	2.639182	-0.402707
16	1	0	-0.356533	-0.841333	-2.196289
17	1	0	-3.585396	0.121705	-1.734841
18	1	0	-0.733747	2.482222	1.036213
19	6	0	1.453031	0.066879	0.076896
20	7	0	2.084215	-1.128576	0.089030
21	1	0	1.567018	-1.995022	0.180322
22	6	0	3.456597	-0.985052	0.004288
23	1	0	4.131598	-1.825888	0.007600
24	7	0	2.456891	0.966570	-0.014145
25	1	0	2.271224	1.961231	-0.028507
26	6	0	3.695000	0.352597	-0.068651
27	1	0	4.617356	0.905632	-0.145216
28	6	0	-2.047977	-1.430439	-0.640655
29	5	0	-1.456808	0.071791	1.531413

TS16/17

Standard orientation:

Center Number	Atomic Number	Atomic Type	Coordinates (Angstroms) X	Y	Z
1	5	0	1.214422	-0.375551	-1.314825
2	5	0	2.892611	-0.025445	-0.755915
3	5	0	1.054136	-0.180664	1.545325
4	5	0	2.243658	1.050994	0.782404
5	5	0	0.032428	0.218504	0.093468
6	5	0	0.880963	-1.362581	0.314750
7	5	0	2.434956	-0.923739	1.005966
8	6	0	0.649497	1.685170	0.113721
9	1	0	2.446169	2.268439	-1.528373
10	1	0	0.854072	-0.660907	-2.414503
11	1	0	3.931857	0.058086	-1.329590
12	1	0	0.679050	-0.107796	2.672874
13	1	0	2.872160	1.806053	1.453868
14	1	0	2.678278	-2.319591	-0.942440
15	1	0	0.542904	2.366546	0.967439
16	1	0	0.204862	-2.347462	0.408234
17	1	0	3.300016	-1.373691	1.686795
18	1	0	0.456390	2.329732	-0.777414
19	6	0	-1.516357	0.032626	0.020368
20	7	0	-2.259233	-1.097908	-0.081622
21	1	0	-1.827769	-2.012586	-0.094447
22	6	0	-3.615795	-0.819712	-0.126834
23	1	0	-4.365043	-1.590970	-0.206735
24	7	0	-2.440111	1.027400	0.030012
25	1	0	-2.176032	1.999504	0.104305

26	6	0	-3.732961	0.531468	-0.055551
27	1	0	-4.602580	1.168667	-0.060102
28	6	0	2.232320	-1.420524	-0.537541
29	5	0	2.006997	1.369556	-0.883898

17

Standard orientation:

Center Number	Atomic Number	Atomic Type	Coordinates (Angstroms) X	Y	Z
1	5	0	0.922601	-0.887367	-1.203506
2	5	0	2.866897	0.012848	-0.731816
3	5	0	1.038773	0.145340	1.400162
4	5	0	2.404165	1.037647	0.617520
5	5	0	0.019176	0.025550	-0.056527
6	5	0	0.928036	-1.400726	0.553919
7	5	0	2.543608	-0.727555	0.916999
8	6	0	0.809053	1.400054	0.294840
9	1	0	2.517337	2.668507	-1.007602
10	1	0	0.545685	-1.334121	-2.241924
11	1	0	3.903384	-0.016018	-1.315882
12	1	0	0.684352	0.296927	2.525339
13	1	0	3.098647	1.792311	1.215658
14	1	0	2.714722	-2.342594	-0.819724
15	1	0	0.345562	2.237300	0.819713
16	1	0	0.445811	-2.403349	0.990946
17	1	0	3.331753	-1.206397	1.665075
18	1	0	1.381887	1.388324	-2.036058
19	6	0	-1.537275	0.004534	-0.022766
20	7	0	-2.362750	-1.068480	-0.006145
21	1	0	-2.009134	-2.014491	0.047896
22	6	0	-3.695412	-0.687723	-0.023116
23	1	0	-4.503949	-1.400765	-0.011061
24	7	0	-2.375050	1.066629	-0.054564
25	1	0	-2.032808	2.016846	-0.100975
26	6	0	-3.704089	0.670327	-0.055228
27	1	0	-4.520908	1.373430	-0.079953
28	6	0	2.186459	-1.450083	-0.503730
29	5	0	1.858712	1.661142	-0.970048

TS17/13

Standard orientation:

Center Number	Atomic Number	Atomic Type	Coordinates (Angstroms) X	Y	Z
1	5	0	-2.509823	-0.204343	1.616961
2	5	0	-3.322056	0.063871	0.017049
3	5	0	-0.731829	0.046281	-1.340416
4	5	0	-2.224522	1.009088	-0.918677
5	5	0	0.251045	-0.092644	0.025335
6	5	0	-0.802752	-1.350182	-0.048541

7	5	0	-2.275671	-0.795549	-1.089531
8	6	0	-0.749139	1.283799	-0.141976
9	1	0	-2.598138	2.399860	1.219866
10	1	0	-2.904411	-0.481828	2.708490
11	1	0	-4.503946	0.049540	-0.113989
12	1	0	-0.279907	0.263933	-2.420238
13	1	0	-2.557270	1.814910	-1.725546
14	1	0	-2.864872	-2.263242	0.619497
15	1	0	-0.316907	2.264588	-0.323575
16	1	0	-0.421976	-2.470130	-0.255960
17	1	0	-2.734845	-1.449513	-1.970333
18	1	0	-0.587705	0.907912	0.936001
19	6	0	1.768988	-0.015306	0.052407
20	7	0	2.603826	-1.093135	0.045774
21	1	0	2.247952	-2.038451	0.013420
22	6	0	3.935104	-0.710043	0.100817
23	1	0	4.743249	-1.423629	0.100614
24	7	0	2.616732	1.051629	0.128282
25	1	0	2.300370	2.009713	0.129884
26	6	0	3.946831	0.644467	0.156997
27	1	0	4.764367	1.344318	0.219248
28	6	0	-2.412740	-1.282920	0.493755
29	5	0	-2.337217	1.291994	0.872266

----

p-2a

Standard orientation:

Center Number	Atomic Number	Atomic Type	Coordinates (Angstroms) X	Y	Z
1	6	0	0.933608	0.813293	1.163632
2	5	0	1.566475	-0.119522	-0.018531
3	5	0	1.421269	1.616725	-0.292067
4	5	0	-0.699825	0.661547	1.330304
5	5	0	0.044426	2.167760	0.714062
6	5	0	0.732537	0.662000	-1.576541
7	5	0	-1.509794	-0.326102	-0.208025
8	5	0	-0.058533	2.204052	-1.051341
9	5	0	-1.384033	1.622860	-0.104779
10	6	0	-0.837911	0.750027	-1.410104
11	1	0	1.516620	1.002874	2.061111
12	1	0	0.933185	-1.096640	-0.321712
13	1	0	2.447475	2.222483	-0.297035
14	1	0	-1.245309	0.708355	2.387915
15	1	0	0.015215	3.098276	1.447829
16	1	0	1.237610	0.480840	-2.637816
17	1	0	-1.069848	-1.391377	-0.588313
18	1	0	-0.189478	3.174696	-1.717463
19	1	0	-2.437174	2.174490	-0.113713
20	1	0	-1.455935	0.729008	-2.305577
21	6	0	-3.093081	-0.565102	-0.017572
22	7	0	-4.064647	0.197297	0.519852
23	1	0	-3.887190	1.135282	0.860469

24	6	0	-5.283599	-0.450494	0.512408
25	1	0	-6.184098	0.000079	0.898945
26	7	0	-3.719005	-1.709125	-0.365333
27	1	0	-3.233158	-2.485914	-0.796376
28	6	0	-5.064493	-1.669405	-0.055306
29	1	0	-5.735812	-2.487960	-0.261165
30	6	0	3.106435	-0.516536	0.055031
31	7	0	4.201782	0.242570	0.266531
32	1	0	4.151015	1.250282	0.352916
33	6	0	5.358174	-0.517323	0.272358
34	1	0	6.335792	-0.087187	0.421317
35	7	0	3.591657	-1.770832	-0.068010
36	1	0	2.994506	-2.570348	-0.233069
37	6	0	4.969926	-1.802990	0.058367
38	1	0	5.541314	-2.714563	-0.014051
39	1	0	-0.900206	-0.557462	0.946213

----

p-2a/19a

Standard orientation:

Center Number	Atomic Number	Atomic Type	Coordinates (Angstroms) X	Y	Z
1	6	0	0.864105	0.734761	1.134797
2	5	0	1.537726	-0.138259	-0.061452
3	5	0	1.389232	1.602593	-0.274579
4	5	0	-0.783058	0.700729	1.280253
5	5	0	0.046588	2.184089	0.751625
6	5	0	0.694527	0.646220	-1.553384
7	5	0	-1.505661	-0.088094	-0.104281
8	5	0	-0.068990	2.184021	-1.048774
9	5	0	-1.411700	1.758001	-0.048082
10	6	0	-0.928572	0.736092	-1.359878
11	1	0	1.428158	0.864062	2.056751
12	1	0	0.939213	-1.117524	-0.453712
13	1	0	2.426610	2.188364	-0.289448
14	1	0	-1.274385	0.674098	2.373356
15	1	0	0.158385	3.134822	1.451109
16	1	0	1.147003	0.455302	-2.635871
17	1	0	-0.840003	-1.536043	-0.302546
18	1	0	-0.155491	3.102726	-1.792103
19	1	0	-2.470635	2.296068	-0.064106
20	1	0	-1.511250	0.766981	-2.278784
21	6	0	-3.022863	-0.558934	0.000671
22	7	0	-3.851046	-1.064070	-0.937596
23	1	0	-3.570424	-1.236495	-1.894886
24	6	0	-5.119478	-1.277790	-0.427819
25	1	0	-5.926887	-1.674588	-1.022635
26	7	0	-3.781916	-0.463848	1.108505
27	1	0	-3.412511	-0.082163	1.973640
28	6	0	-5.073516	-0.892071	0.878125
29	1	0	-5.835246	-0.886350	1.641639
30	6	0	3.076396	-0.529952	0.021469

31	7	0	4.149372	0.226780	0.333666
32	1	0	4.076758	1.224035	0.493884
33	6	0	5.319283	-0.512785	0.321834
34	1	0	6.282459	-0.080066	0.540776
35	7	0	3.589406	-1.763552	-0.180751
36	1	0	3.016193	-2.558574	-0.429468
37	6	0	4.963551	-1.782955	-0.006919
38	1	0	5.554036	-2.676773	-0.129662
39	1	0	-0.739452	-1.149746	0.414980

19a

Standard orientation:

Center Number	Atomic Number	Atomic Type	Coordinates (Angstroms) X	Y	Z
1	6	0	-0.803104	1.152395	-1.304414
2	5	0	-1.128557	-0.051663	-0.217590
3	5	0	-1.288416	1.709700	0.177186
4	5	0	0.822151	1.215811	-1.386255
5	5	0	-0.148148	2.631772	-0.839083
6	5	0	-0.272274	0.803956	1.312999
7	5	0	1.493756	0.592662	0.062633
8	5	0	0.020891	2.578745	0.981142
9	5	0	1.434295	2.391978	-0.136917
10	6	0	1.209487	1.510133	1.263363
11	1	0	-1.430672	1.278693	-2.183456
12	1	0	-0.437043	-1.026142	-0.330263
13	1	0	-2.408985	2.049467	0.387631
14	1	0	1.361923	1.148729	-2.453919
15	1	0	-0.464238	3.601840	-1.441705
16	1	0	-0.553423	0.314035	2.360288
17	1	0	-0.169977	3.479343	1.725758
18	1	0	2.378868	3.104555	-0.219756
19	1	0	1.808883	1.600765	2.163841
20	6	0	2.626188	-0.478561	0.059752
21	7	0	3.250907	-0.929292	-1.048707
22	1	0	3.015915	-0.579070	-1.971544
23	6	0	4.213214	-1.868254	-0.741938
24	1	0	4.830895	-2.337494	-1.491278
25	7	0	3.199475	-1.163637	1.075051
26	1	0	2.942065	-1.038400	2.045938
27	6	0	4.175636	-2.025532	0.611777
28	1	0	4.748514	-2.662656	1.266918
29	6	0	-2.622595	-0.644678	-0.053253
30	7	0	-3.836138	-0.075657	0.106583
31	1	0	-3.954435	0.925592	0.200112
32	6	0	-4.845580	-1.019182	0.150309
33	1	0	-5.883934	-0.754915	0.273721
34	7	0	-2.888767	-1.968391	-0.110274
35	1	0	-2.160354	-2.657550	-0.243913
36	6	0	-4.240507	-2.230313	0.012875
37	1	0	-4.647261	-3.228815	-0.008341

TS19a/20a

Standard orientation:

Center Number	Atomic Number	Atomic Type	Coordinates (Angstroms) X	Y	Z
1	6	0	0.567951	1.632063	1.408446
2	5	0	0.462282	0.273302	0.552945
3	5	0	1.121593	1.931700	-0.064644
4	5	0	-1.021448	1.445158	1.373486
5	5	0	-0.194217	2.920096	0.743469
6	5	0	0.145299	0.838500	-1.165195
7	5	0	-1.362522	0.535563	-0.117026
8	5	0	-0.188463	2.651065	-1.050740
9	5	0	-1.709320	2.346408	-0.020217
10	6	0	-1.298971	1.524483	-1.349912
11	1	0	1.148102	1.737452	2.316274
12	1	0	0.408725	-0.801522	1.064872
13	1	0	2.250547	2.232871	-0.256459
14	1	0	-1.630890	1.294630	2.386982
15	1	0	-0.078483	3.983967	1.248669
16	1	0	0.583843	0.217048	-2.075824
17	1	0	0.026260	3.483512	-1.864580
18	1	0	-2.756943	2.900136	0.022166
19	1	0	-1.882190	1.483251	-2.260955
20	6	0	-2.363885	-0.677997	-0.103668
21	7	0	-3.094453	-1.083843	0.952239
22	1	0	-3.058226	-0.614201	1.850363
23	6	0	-3.874372	-2.179662	0.635754
24	1	0	-4.537046	-2.647340	1.346647
25	7	0	-2.685155	-1.535016	-1.094367
26	1	0	-2.282627	-1.491627	-2.022648
27	6	0	-3.610008	-2.471479	-0.667867
28	1	0	-3.993047	-3.245902	-1.313501
29	6	0	2.631644	-0.519080	0.097929
30	7	0	3.749324	-0.204751	-0.605310
31	1	0	3.829004	0.648393	-1.141087
32	6	0	4.754662	-1.157645	-0.489942
33	1	0	5.712126	-1.067577	-0.979157
34	7	0	2.971253	-1.707939	0.655643
35	1	0	2.344161	-2.216963	1.263183
36	6	0	4.255124	-2.124200	0.321862
37	1	0	4.691023	-3.043154	0.681629

20a

Standard orientation:

Center Number	Atomic Number	Atomic Type	Coordinates (Angstroms) X	Y	Z
1	6	0	-2.307662	-1.341420	0.577434
2	5	0	-1.074543	-0.604241	1.277883

3	5	0	-2.728395	0.159305	0.948733
4	5	0	-1.073080	-1.327450	-0.438936
5	5	0	-2.727176	-0.570202	-0.763768
6	5	0	-1.224182	1.175113	0.936232
7	5	0	-0.073081	0.117970	-0.049869
8	5	0	-2.399277	1.200437	-0.501940
9	5	0	-1.230403	0.146677	-1.491975
10	6	0	-0.817357	1.419980	-0.601385
11	1	0	-2.788490	-2.233831	0.955042
12	1	0	-0.590964	-1.023215	2.274167
13	1	0	-3.625415	0.368041	1.690210
14	1	0	-0.596196	-2.343511	-0.827942
15	1	0	-3.623735	-0.961041	-1.428082
16	1	0	-0.976861	1.995728	1.753671
17	1	0	-3.133013	2.054105	-0.862738
18	1	0	-0.986400	0.126674	-2.649656
19	1	0	-0.344843	2.312115	-0.992371
20	6	0	1.500752	0.030768	-0.017311
21	7	0	2.247905	-1.065174	-0.251605
22	1	0	1.849031	-1.964469	-0.497580
23	6	0	3.595735	-0.791970	-0.123671
24	1	0	4.357676	-1.539871	-0.277631
25	7	0	2.391611	1.002842	0.264437
26	1	0	2.132112	1.951337	0.508656
27	6	0	3.687877	0.525490	0.210352
28	1	0	4.545092	1.149133	0.410131

----

o-2a

Standard orientation:

Center Number	Atomic Number	Atomic Type	Coordinates (Angstroms) X	Y	Z
1	6	0	0.500743	1.130375	0.767708
2	5	0	-0.149967	-0.162335	1.559138
3	5	0	1.612765	-0.016243	1.389171
4	5	0	2.023619	0.967203	0.000000
5	5	0	1.089635	-1.601412	0.921129
6	5	0	-0.149967	-0.162335	-1.559138
7	5	0	2.377887	-0.790976	0.000000
8	5	0	1.612765	-0.016243	-1.389171
9	1	0	0.438959	2.112934	1.227193
10	1	0	-1.090824	-0.609300	0.970623
11	1	0	2.181043	0.191044	2.414934
12	1	0	0.438959	2.112934	-1.227193
13	1	0	2.804761	1.855345	0.000000
14	1	0	1.255096	-2.578273	1.575365
15	1	0	-1.090824	-0.609300	-0.970623
16	1	0	3.513723	-1.132858	0.000000
17	1	0	2.181043	0.191044	-2.414934
18	1	0	1.255096	-2.578273	-1.575365
19	6	0	-0.539870	-0.002958	-3.094121
20	7	0	0.181647	0.392944	-4.163776

21	1	0	1.164752	0.626423	-4.096900
22	6	0	-0.569536	0.368622	-5.325477
23	1	0	-0.164540	0.647164	-6.285367
24	7	0	-1.762437	-0.272683	-3.601560
25	1	0	-2.528146	-0.600179	-3.027296
26	6	0	-1.810976	-0.056208	-4.967445
27	1	0	-2.701613	-0.220084	-5.552862
28	6	0	-0.539870	-0.002958	3.094121
29	7	0	-1.762437	-0.272683	3.601560
30	1	0	-2.528146	-0.600179	3.027296
31	6	0	-1.810976	-0.056208	4.967445
32	1	0	-2.701613	-0.220084	5.552862
33	7	0	0.181647	0.392944	4.163776
34	1	0	1.164752	0.626423	4.096900
35	6	0	-0.569536	0.368622	5.325477
36	1	0	-0.164540	0.647164	6.285367
37	6	0	0.500743	1.130375	-0.767708
38	5	0	1.089635	-1.601412	-0.921129
39	1	0	0.206911	-1.904815	0.000000

TSo-2a/21a

Standard orientation:

Center Number	Atomic Number	Atomic Type	Coordinates (Angstroms) X	Y	Z
1	6	0	-0.694745	0.536680	-1.190636
2	5	0	-1.600292	-0.152653	0.224103
3	5	0	-1.357282	1.573844	0.073082
4	5	0	0.076240	2.144951	-0.888861
5	5	0	-0.716894	0.906656	1.620817
6	5	0	1.537337	-0.130187	0.084955
7	5	0	0.041020	2.337936	0.827827
8	5	0	1.462773	1.678331	0.083747
9	1	0	-1.204297	0.558225	-2.150738
10	1	0	-0.897509	-0.652055	-0.828928
11	1	0	-2.372475	2.149997	-0.180487
12	1	0	1.277062	0.618696	-2.192703
13	1	0	-0.074336	2.918737	-1.771031
14	1	0	-1.444022	0.888547	2.564806
15	1	0	0.922959	-1.147218	0.294696
16	1	0	-0.082690	3.436753	1.264501
17	1	0	2.480355	2.235375	-0.193680
18	1	0	1.665624	1.070472	2.511929
19	6	0	3.078179	-0.541357	-0.029806
20	7	0	4.197237	0.200726	-0.165945
21	1	0	4.167979	1.211797	-0.213603
22	6	0	5.337983	-0.582340	-0.155900
23	1	0	6.328931	-0.167145	-0.248463
24	7	0	3.535203	-1.809943	0.060085
25	1	0	2.915618	-2.598149	0.191647
26	6	0	4.916534	-1.866938	-0.010559
27	1	0	5.466696	-2.792398	0.048801

28	6	0	-3.127345	-0.540174	0.034439
29	7	0	-4.177086	0.230553	-0.308132
30	1	0	-4.073853	1.223781	-0.488636
31	6	0	-5.357377	-0.483386	-0.292233
32	1	0	-6.310149	-0.038633	-0.532731
33	7	0	-3.662069	-1.758873	0.263987
34	1	0	-3.112226	-2.554846	0.563311
35	6	0	-5.029946	-1.754548	0.075224
36	1	0	-5.641074	-2.631790	0.217638
37	6	0	0.826547	0.659366	-1.205439
38	5	0	0.961229	0.952477	1.554867
39	1	0	-0.985237	-0.887968	0.940467

21a

Standard orientation:

Center Number	Atomic Number	Atomic Type	Coordinates (Angstroms) X	Y	Z
1	6	0	-0.687842	0.503443	-1.208730
2	5	0	-1.609651	-0.176549	0.285969
3	5	0	-1.357813	1.551403	0.094024
4	5	0	0.086580	2.119616	-0.874702
5	5	0	-0.742400	0.927403	1.638875
6	5	0	1.551966	-0.130141	0.082403
7	5	0	0.017971	2.338202	0.836445
8	5	0	1.462268	1.664888	0.125306
9	1	0	-1.189597	0.607904	-2.168581
10	1	0	-0.871652	-0.616412	-0.925877
11	1	0	-2.371586	2.120543	-0.182101
12	1	0	1.317240	0.642394	-2.177639
13	1	0	-0.073397	2.881087	-1.766181
14	1	0	-1.479823	0.932287	2.577809
15	1	0	0.942979	-1.129290	0.359570
16	1	0	-0.090390	3.455933	1.228276
17	1	0	2.479114	2.231488	-0.136788
18	1	0	1.636923	1.039191	2.542872
19	6	0	3.092961	-0.536541	-0.031292
20	7	0	4.201527	0.201453	-0.250715
21	1	0	4.163925	1.207366	-0.360826
22	6	0	5.348472	-0.572056	-0.218586
23	1	0	6.333147	-0.158173	-0.367076
24	7	0	3.562395	-1.792802	0.133310
25	1	0	2.953299	-2.574205	0.335895
26	6	0	4.941560	-1.845964	0.027606
27	1	0	5.500660	-2.761573	0.136014
28	6	0	-3.139062	-0.539452	0.046215
29	7	0	-4.178120	0.240776	-0.309374
30	1	0	-4.064895	1.234657	-0.480465
31	6	0	-5.361560	-0.466072	-0.324569
32	1	0	-6.306927	-0.013713	-0.579812
33	7	0	-3.686126	-1.757496	0.252260
34	1	0	-3.146134	-2.558051	0.557587

35	6	0	-5.048892	-1.743464	0.036769
36	1	0	-5.668280	-2.618097	0.158210
37	6	0	0.851206	0.655511	-1.196491
38	5	0	0.927014	0.940689	1.587698
39	1	0	-1.023065	-1.076841	0.805136

TS21a/22a

Standard orientation:

Center Number	Atomic Number	Atomic Type	Coordinates (Angstroms) X	Y	Z
1	6	0	-0.653409	0.529947	-1.272906
2	5	0	-1.615287	-0.208694	0.298263
3	5	0	-1.355563	1.536415	0.054640
4	5	0	0.109002	2.121980	-0.860965
5	5	0	-0.801948	0.900628	1.602259
6	5	0	1.565896	-0.128107	0.064853
7	5	0	-0.016846	2.313013	0.855044
8	5	0	1.459580	1.651839	0.174428
9	1	0	-1.125325	0.706436	-2.238298
10	1	0	-0.840565	-0.571029	-1.058418
11	1	0	-2.365188	2.104780	-0.239893
12	1	0	1.399930	0.713448	-2.165347
13	1	0	-0.025603	2.906867	-1.736258
14	1	0	-1.567417	0.933174	2.521606
15	1	0	0.947098	-1.111088	0.365344
16	1	0	-0.117721	3.428618	1.254998
17	1	0	2.480279	2.233827	-0.034626
18	1	0	1.559309	0.949465	2.575771
19	6	0	3.106699	-0.532743	-0.026874
20	7	0	4.211536	0.206647	-0.260080
21	1	0	4.169599	1.210549	-0.386080
22	6	0	5.362382	-0.560284	-0.210777
23	1	0	6.345234	-0.144494	-0.365852
24	7	0	3.581907	-1.782947	0.163733
25	1	0	2.976870	-2.563994	0.379679
26	6	0	4.961525	-1.830986	0.060698
27	1	0	5.525123	-2.741318	0.188530
28	6	0	-3.149631	-0.545858	0.048440
29	7	0	-4.196232	0.261343	-0.213531
30	1	0	-4.082073	1.264663	-0.316163
31	6	0	-5.379894	-0.441346	-0.260334
32	1	0	-6.330372	0.030278	-0.454600
33	7	0	-3.694404	-1.777651	0.162937
34	1	0	-3.147853	-2.600338	0.387196
35	6	0	-5.060894	-1.745741	-0.016990
36	1	0	-5.679216	-2.627481	0.043374
37	6	0	0.899049	0.682490	-1.201601
38	5	0	0.868920	0.890319	1.603582
39	1	0	-1.025498	-1.193944	0.629168

22a

Standard orientation:

Center Number	Atomic Number	Atomic Type	Coordinates (Angstroms) X	Y	Z
1	6	0	-0.018201	1.649668	-1.504570
2	5	0	-1.772885	-0.363661	-0.209669
3	5	0	-1.210230	1.730862	-0.099211
4	5	0	0.355428	2.563311	-0.127313
5	5	0	-1.137085	0.371116	1.095056
6	5	0	1.778131	0.181118	-0.116722
7	5	0	-0.137189	1.760518	1.335976
8	5	0	1.546851	1.621766	0.791524
9	1	0	-0.126532	2.398199	-2.286815
10	1	0	-0.386705	0.695082	-1.848870
11	1	0	-2.133727	2.454808	-0.294658
12	1	0	2.210698	1.991252	-1.511242
13	1	0	0.444746	3.723654	-0.339472
14	1	0	-1.916209	0.417499	2.013722
15	1	0	0.806924	-0.604383	0.065443
16	1	0	-0.325168	2.383729	2.328793
17	1	0	2.466625	2.175420	1.296189
18	1	0	1.017722	-0.549094	2.213607
19	6	0	3.164668	-0.540484	-0.086982
20	7	0	4.174557	-0.469608	-0.981418
21	1	0	4.128015	0.080792	-1.828589
22	6	0	5.253214	-1.247218	-0.589317
23	1	0	6.159285	-1.317780	-1.169589
24	7	0	3.615180	-1.383166	0.867066
25	1	0	3.078106	-1.595700	1.699536
26	6	0	4.894944	-1.829112	0.585874
27	1	0	5.428815	-2.507089	1.232551
28	6	0	-3.317844	-0.667886	-0.157748
29	7	0	-4.212891	-0.270984	0.772476
30	1	0	-3.927346	0.280320	1.574706
31	6	0	-5.479193	-0.736380	0.494218
32	1	0	-6.330422	-0.524191	1.121628
33	7	0	-4.050398	-1.395963	-1.034055
34	1	0	-3.648105	-1.830139	-1.854601
35	6	0	-5.378068	-1.457063	-0.659543
36	1	0	-6.122290	-1.992029	-1.227848
37	6	0	1.437645	1.468048	-0.954280
38	5	0	0.657341	0.132867	1.300263
39	1	0	-1.254379	-0.869224	-1.168401

TS22a/23a

Standard orientation:

Center Number	Atomic Number	Atomic Type	Coordinates (Angstroms) X	Y	Z
1	6	0	-0.455807	1.476188	-1.558471
2	5	0	-1.714930	-0.452323	-0.155421

3	5	0	-1.308754	1.572759	-0.187301
4	5	0	0.313219	2.477358	-0.365184
5	5	0	-1.083381	0.339270	1.141587
6	5	0	1.856874	0.306107	-0.076250
7	5	0	-0.191033	1.809943	1.228647
8	5	0	1.526832	1.794771	0.762039
9	1	0	-0.682723	2.168323	-2.369745
10	1	0	-0.142506	0.518351	-1.936627
11	1	0	-2.224366	2.341523	-0.204276
12	1	0	2.208285	2.039736	-1.593960
13	1	0	0.273950	3.634235	-0.622272
14	1	0	-1.816955	0.392397	2.094382
15	1	0	0.847525	-0.464747	0.203314
16	1	0	-0.422324	2.501415	2.165588
17	1	0	2.305611	2.509304	1.299866
18	1	0	1.152360	-0.338231	2.291287
19	6	0	3.210746	-0.480495	-0.071193
20	7	0	4.184952	-0.504480	-1.005834
21	1	0	4.132359	0.005511	-1.877998
22	6	0	5.241491	-1.308666	-0.612068
23	1	0	6.119222	-1.453755	-1.221504
24	7	0	3.661004	-1.287925	0.911664
25	1	0	3.143197	-1.434631	1.770955
26	6	0	4.906339	-1.807059	0.608741
27	1	0	5.436420	-2.471189	1.272753
28	6	0	-3.268502	-0.719204	-0.120233
29	7	0	-4.174696	-0.190328	0.730257
30	1	0	-3.896015	0.446664	1.468668
31	6	0	-5.445768	-0.658077	0.477548
32	1	0	-6.306050	-0.351222	1.051090
33	7	0	-3.997825	-1.532329	-0.919826
34	1	0	-3.586593	-2.071261	-1.670794
35	6	0	-5.335362	-1.517547	-0.575828
36	1	0	-6.079133	-2.102164	-1.093511
37	6	0	1.531641	1.554069	-0.896622
38	5	0	0.738017	0.266736	1.346241
39	1	0	-1.169188	-1.073181	-1.024079

23a

Standard orientation:

Center Number	Atomic Number	Atomic Type	Coordinates (Angstroms) X	Y	Z
1	6	0	-1.308070	0.623829	-1.518461
2	5	0	-1.884333	-0.203320	-0.355354
3	5	0	-1.325536	1.469556	-0.079820
4	5	0	0.369037	2.362857	-0.283630
5	5	0	-1.046433	0.091744	1.145146
6	5	0	2.025772	0.369305	-0.052378
7	5	0	-0.110751	1.587821	1.231697
8	5	0	1.596093	1.736481	0.960198
9	1	0	-1.985161	1.000524	-2.282670

10	1	0	-0.333951	0.371104	-1.920293
11	1	0	-2.254970	2.208550	0.101178
12	1	0	2.390994	2.219112	-1.321322
13	1	0	0.137584	3.505009	-0.524118
14	1	0	-1.611042	-0.305029	2.113200
15	1	0	1.028453	-0.507263	0.181343
16	1	0	-0.544901	2.067257	2.233662
17	1	0	2.329471	2.322461	1.687588
18	1	0	1.252101	-0.555148	2.243614
19	6	0	3.404242	-0.382772	-0.111687
20	7	0	4.343918	-0.291677	-1.074361
21	1	0	4.227670	0.280371	-1.901801
22	6	0	5.452599	-1.060209	-0.772203
23	1	0	6.315889	-1.115556	-1.416168
24	7	0	3.928532	-1.228306	0.798795
25	1	0	3.453432	-1.455809	1.664746
26	6	0	5.186560	-1.658545	0.422283
27	1	0	5.774431	-2.335367	1.021786
28	6	0	-3.411524	-0.570807	-0.192446
29	7	0	-4.407258	0.316556	-0.008757
30	1	0	-4.223909	1.308512	0.100648
31	6	0	-5.639411	-0.310910	0.057372
32	1	0	-6.560272	0.231442	0.201235
33	7	0	-4.025545	-1.770543	-0.243245
34	1	0	-3.536138	-2.643144	-0.391331
35	6	0	-5.398564	-1.640507	-0.088571
36	1	0	-6.065813	-2.487470	-0.096277
37	6	0	1.656973	1.655082	-0.747248
38	5	0	0.919985	0.140372	1.332456
39	1	0	-1.047401	-0.996792	0.212149

TSm-2a/24a

Standard orientation:

Center Number	Atomic Number	Atomic Type	Coordinates (Angstroms) X	Y	Z
1	6	0	-0.787669	0.739706	1.290148
2	5	0	-1.430562	-0.162957	0.005426
3	5	0	-0.048701	2.229595	0.960351
4	5	0	0.859642	0.753786	1.371500
5	5	0	0.831440	0.862563	-1.413878
6	1	0	-1.327160	0.716723	2.233806
7	1	0	-2.500901	2.132263	0.056417
8	1	0	-0.908191	-1.252045	-0.003858
9	1	0	-0.182705	3.147501	1.698413
10	1	0	1.373304	0.758940	2.454829
11	1	0	-1.377184	0.793711	-2.194224
12	1	0	-0.259536	3.240908	-1.570353
13	1	0	0.614219	-1.786944	-0.080566
14	1	0	2.404601	2.417927	-0.007559
15	1	0	1.316120	0.900494	-2.507136
16	6	0	-3.005873	-0.550216	0.002138

17	7	0	-3.431287	-1.832394	-0.015975
18	7	0	-4.147161	0.170396	0.015906
19	1	0	-2.787648	-2.613087	-0.028452
20	1	0	-4.148826	1.182790	0.031410
21	6	0	-5.267819	-0.639555	0.007070
22	6	0	-4.810699	-1.920869	-0.013454
23	1	0	-6.271086	-0.243763	0.015934
24	1	0	-5.336148	-2.862515	-0.026053
25	5	0	-1.418774	1.644427	0.036201
26	5	0	1.588360	0.099158	-0.033667
27	5	0	1.345046	1.871313	-0.007087
28	6	0	-0.806477	0.788510	-1.268352
29	5	0	-0.082964	2.286308	-0.890153
30	6	0	3.039395	-0.489290	-0.022458
31	7	0	3.854806	-0.742951	-1.068787
32	1	0	3.569541	-0.579005	-2.027795
33	6	0	5.095120	-1.172767	-0.648608
34	1	0	5.891594	-1.419880	-1.332748
35	7	0	3.784968	-0.773708	1.065592
36	1	0	3.427882	-0.638552	2.005416
37	6	0	5.050503	-1.191262	0.716602
38	1	0	5.801115	-1.457156	1.444133
39	1	0	1.015399	-1.494220	-0.660744

24a

Standard orientation:

Center Number	Atomic Number	Atomic Type	Coordinates (Angstroms) X	Y	Z
1	6	0	1.107020	0.610589	1.288840
2	5	0	0.021125	0.964248	0.000000
3	5	0	2.715006	0.160316	0.914205
4	5	0	1.463308	-0.974211	1.421028
5	5	0	1.463308	-0.974211	-1.421028
6	1	0	0.980795	1.166607	2.214839
7	1	0	2.073992	2.528915	0.000000
8	1	0	-0.918596	0.203852	0.000000
9	1	0	3.616257	0.494171	1.606912
10	1	0	1.519960	-1.504058	2.490113
11	1	0	0.980795	1.166607	-2.214839
12	1	0	3.616257	0.494171	-1.606912
13	1	0	3.184664	-2.312828	0.000000
14	1	0	1.519960	-1.504058	-2.490113
15	6	0	-0.691573	2.419898	0.000000
16	7	0	-2.034980	2.562695	0.000000
17	7	0	-0.230465	3.688944	0.000000
18	1	0	-2.658545	1.766265	0.000000
19	1	0	0.758400	3.906485	0.000000
20	6	0	-1.260942	4.611486	0.000000
21	6	0	-2.415502	3.892213	0.000000
22	1	0	-1.087826	5.676078	0.000000
23	1	0	-3.447401	4.205402	0.000000

24	5	0	1.835671	1.365374	0.000000
25	5	0	0.770828	-1.606217	0.000000
26	5	0	2.509857	-1.331033	0.000000
27	6	0	1.107020	0.610589	-1.288840
28	5	0	2.715006	0.160316	-0.914205
29	6	0	-0.433559	-2.571138	0.000000
30	7	0	-1.058140	-3.115437	-1.069224
31	1	0	-0.773519	-2.899847	-2.019322
32	6	0	-2.046213	-3.986169	-0.685296
33	1	0	-2.655184	-4.531979	-1.389115
34	7	0	-1.058140	-3.115437	1.069224
35	1	0	-0.773519	-2.899847	2.019322
36	6	0	-2.046213	-3.986169	0.685296
37	1	0	-2.655184	-4.531979	1.389115

TS24a/25a

Standard orientation:

Center Number	Atomic Number	Atomic Type	Coordinates (Angstroms) X	Y	Z
1	6	0	-0.606385	1.583102	1.422000
2	5	0	-0.450685	0.273211	0.524579
3	5	0	0.148864	2.910466	0.810971
4	5	0	1.004121	1.450566	1.418415
5	5	0	1.408611	1.580219	-1.425764
6	1	0	-1.211918	1.641389	2.316605
7	1	0	-2.231168	2.166587	-0.351266
8	1	0	-0.349228	-0.849985	0.903334
9	1	0	-0.020980	3.960639	1.328770
10	1	0	1.582550	1.296295	2.447172
11	1	0	-0.491876	0.353878	-1.820264
12	1	0	-0.171095	3.435719	-1.816323
13	1	0	2.704610	3.024556	0.184479
14	1	0	1.932793	1.448131	-2.481912
15	6	0	-2.600807	-0.533870	-0.001396
16	7	0	-2.861302	-1.799937	0.412100
17	7	0	-3.795434	-0.158412	-0.528515
18	1	0	-2.166330	-2.370926	0.873008
19	1	0	-3.950859	0.759463	-0.921844
20	6	0	-4.767151	-1.148331	-0.445874
21	6	0	-4.166625	-2.204471	0.159703
22	1	0	-5.773491	-1.019416	-0.813116
23	1	0	-4.545706	-3.178803	0.425926
24	5	0	-1.112630	1.944473	-0.046678
25	5	0	1.436352	0.616149	-0.065970
26	5	0	1.700771	2.407639	0.041357
27	6	0	-0.042145	0.922296	-1.012110
28	5	0	0.196806	2.683599	-0.977732
29	6	0	2.361587	-0.653188	-0.066162
30	7	0	2.993119	-1.228302	-1.110274
31	1	0	2.917860	-0.872200	-2.056160
32	6	0	3.779295	-2.287892	-0.704230

33	1	0	4.366543	-2.875436	-1.392218
34	7	0	2.758977	-1.367609	1.006556
35	1	0	2.459023	-1.145556	1.948802
36	6	0	3.633594	-2.373918	0.648627
37	1	0	4.072518	-3.048510	1.366695

25a

Standard orientation:

Center Number	Atomic Number	Atomic Type	Coordinates (Angstroms) X	Y	Z
1	6	0	2.314485	-1.443035	-0.113968
2	5	0	1.032866	-1.126237	0.768560
3	5	0	2.758868	-0.117801	-0.910325
4	5	0	1.121648	-0.939745	-1.066876
5	5	0	0.740959	1.679797	0.139846
6	1	0	2.802186	-2.406424	-0.169050
7	1	0	3.485244	-0.413096	1.787252
8	1	0	0.517711	-1.903246	1.498608
9	1	0	3.689130	-0.130637	-1.639273
10	1	0	0.673080	-1.641522	-1.908561
11	1	0	0.983046	0.761914	2.256309
12	1	0	3.133280	2.166721	0.429621
13	1	0	1.095120	1.355647	-2.326435
14	1	0	0.177722	2.690189	0.394807
15	5	0	2.691643	-0.288028	0.922547
16	5	0	0.074050	0.129420	-0.072477
17	5	0	1.263865	0.871505	-1.257979
18	6	0	1.194736	0.542095	1.214101
19	5	0	2.386952	1.295971	0.137671
20	6	0	-1.495757	0.009338	-0.014265
21	7	0	-2.388926	1.017499	-0.075173
22	1	0	-2.113403	1.991218	-0.142864
23	6	0	-3.684117	0.546725	-0.042660
24	1	0	-4.542548	1.198835	-0.083172
25	7	0	-2.247321	-1.108969	0.057745
26	1	0	-1.853203	-2.041128	0.110072
27	6	0	-3.594183	-0.811996	0.043048
28	1	0	-4.358865	-1.571357	0.092305

TS25a/26a

Standard orientation:

Center Number	Atomic Number	Atomic Type	Coordinates (Angstroms) X	Y	Z
1	6	0	-2.414105	-1.229288	0.209813
2	5	0	-0.913707	-1.313833	0.930484
3	5	0	-0.066207	-0.025877	0.114562
4	5	0	-2.475084	1.404946	-0.400656
5	5	0	-2.606045	-0.181313	-1.075375
6	5	0	-0.932594	1.471385	0.447684

7	5	0	-0.953670	0.252766	1.609169
8	5	0	-3.062394	0.180869	0.600004
9	1	0	-0.477749	-2.337415	1.343796
10	1	0	-3.302729	-0.402328	-2.003904
11	1	0	-0.441070	2.554776	0.471583
12	1	0	-1.001255	0.484040	2.768834
13	1	0	-3.000063	-2.142582	0.252126
14	6	0	-1.116212	0.648550	-1.054655
15	5	0	-1.100413	-1.113554	-0.808255
16	1	0	-0.769076	-1.868229	-1.656688
17	1	0	-2.922153	2.405275	-0.843195
18	1	0	-0.691803	1.026526	-1.978440
19	1	0	-3.960776	0.290192	1.360260
20	6	0	1.506163	-0.003266	0.011433
21	7	0	2.325160	-1.059454	-0.161334
22	1	0	1.987320	-2.011418	-0.251125
23	6	0	3.648182	-0.669584	-0.179894
24	1	0	4.459716	-1.368723	-0.308252
25	7	0	2.327821	1.061073	0.104244
26	1	0	1.991564	2.006273	0.255335
27	6	0	3.650034	0.684268	-0.009754
28	1	0	4.463403	1.391244	0.039559

26a

Standard orientation:

Center Number	Atomic Number	Atomic Type	Coordinates (Angstroms) X	Y	Z
1	6	0	2.324228	1.097699	0.355091
2	5	0	0.805555	1.624866	0.550558
3	5	0	0.067701	0.178150	0.105926
4	5	0	2.323980	-1.542516	-0.535114
5	5	0	2.707952	0.051856	-0.935217
6	5	0	1.055845	-1.261353	0.568531
7	5	0	1.246795	0.258562	1.471933
8	5	0	2.716085	-0.567390	0.789095
9	1	0	0.435877	2.695036	0.889082
10	1	0	3.492053	0.335161	-1.771850
11	1	0	0.443752	-2.206606	0.946929
12	1	0	1.185209	0.342215	2.650200
13	1	0	3.096890	1.825049	0.582511
14	6	0	1.096259	-0.620636	-1.086353
15	5	0	1.211945	1.073826	-1.001830
16	1	0	1.125166	1.758819	-1.961121
17	1	0	2.705469	-2.555520	-1.003488
18	1	0	0.596973	-0.990808	-1.976155
19	1	0	3.620200	-0.753505	1.526965
20	6	0	-1.496959	0.029848	0.024213
21	7	0	-2.390555	1.004240	-0.240053
22	1	0	-2.125373	1.965171	-0.424857
23	6	0	-3.684024	0.523796	-0.198352
24	1	0	-4.543253	1.149411	-0.382917

25	7	0	-2.241523	-1.074212	0.231216
26	1	0	-1.840416	-1.972666	0.476645
27	6	0	-3.589095	-0.801883	0.105490
28	1	0	-4.349744	-1.554904	0.239965

TS26a/20a

Standard orientation:

Center Number	Atomic Number	Atomic Type	Coordinates (Angstroms) X	Y	Z
1	6	0	-2.410392	1.126567	-0.469586
2	5	0	-0.872829	1.612662	-0.093625
3	5	0	-0.052261	0.101010	-0.009805
4	5	0	-2.451772	-1.409856	0.478655
5	5	0	-3.089351	0.168357	0.606315
6	5	0	-1.070111	-1.239641	-0.578694
7	5	0	-1.145824	0.423675	-1.358723
8	5	0	-2.629343	-0.501000	-0.981728
9	1	0	-0.460209	2.702021	-0.315331
10	1	0	-3.883187	0.522988	1.407500
11	1	0	-0.528860	-2.198075	-1.023947
12	1	0	-0.881878	0.669158	-2.485301
13	1	0	-3.012177	1.930907	-0.886718
14	6	0	-1.015358	-0.808656	1.085624
15	5	0	-0.998050	0.746687	1.368975
16	1	0	-1.011761	1.218034	2.454409
17	1	0	-2.880967	-2.456596	0.819682
18	1	0	-0.579107	-1.470175	1.831169
19	1	0	-3.470610	-0.741576	-1.779251
20	6	0	1.520314	0.013805	-0.000123
21	7	0	2.393684	1.027965	0.157386
22	1	0	2.106986	1.990494	0.298209
23	6	0	3.697985	0.583657	0.069259
24	1	0	4.545220	1.244055	0.168043
25	7	0	2.286555	-1.078859	-0.185336
26	1	0	1.903082	-2.000867	-0.361759
27	6	0	3.629723	-0.759837	-0.152017
28	1	0	4.405873	-1.496807	-0.286843

m-2a

Standard orientation:

Center Number	Atomic Number	Atomic Type	Coordinates (Angstroms) X	Y	Z
1	6	0	0.728234	0.972717	1.286961
2	5	0	-0.289423	1.442177	0.000000
3	5	0	2.213608	0.237948	0.873803
4	5	0	0.728234	-0.629440	1.428180
5	5	0	0.728234	-0.629440	-1.428180
6	1	0	0.728027	1.548969	2.207720
7	1	0	2.062016	2.680876	0.000000

8	1	0	-1.312848	0.788085	0.000000
9	1	0	3.150854	0.296853	1.595628
10	1	0	0.721276	-1.177995	2.483825
11	1	0	0.728027	1.548969	-2.207720
12	1	0	3.150854	0.296853	-1.595628
13	1	0	-0.620462	-1.026009	0.920147
14	1	0	2.432776	-2.175632	0.000000
15	1	0	0.721276	-1.177995	-2.483825
16	6	0	-0.796641	2.978132	0.000000
17	7	0	-2.104314	3.311517	0.000000
18	7	0	-0.155504	4.165726	0.000000
19	1	0	-2.834990	2.611504	0.000000
20	1	0	0.854739	4.235537	0.000000
21	6	0	-1.042233	5.227693	0.000000
22	6	0	-2.288556	4.682274	0.000000
23	1	0	-0.717708	6.256225	0.000000
24	1	0	-3.264413	5.141099	0.000000
25	5	0	1.592709	1.588698	0.000000
26	5	0	0.000510	-1.594880	0.000000
27	5	0	1.707855	-1.224638	0.000000
28	6	0	0.728234	0.972717	-1.286961
29	5	0	2.213608	0.237948	-0.873803
30	6	0	-0.278301	-3.160214	0.000000
31	7	0	-1.474804	-3.785679	0.000000
32	1	0	-2.358886	-3.293236	0.000000
33	6	0	-1.325766	-5.160478	0.000000
34	1	0	-2.165280	-5.837433	0.000000
35	7	0	0.624268	-4.156527	0.000000
36	1	0	1.621314	-3.965580	0.000000
37	6	0	0.015976	-5.395199	0.000000
38	1	0	0.574018	-6.318060	0.000000
39	1	0	-0.620462	-1.026009	-0.920147

TSm-2a/27a

Standard orientation:

Center Number	Atomic Number	Atomic Type	Coordinates (Angstroms) X	Y	Z
1	6	0	0.921891	-0.869027	-0.805440
2	5	0	1.785089	-0.499033	0.350705
3	5	0	0.559049	0.771833	-1.299215
4	5	0	-0.632329	-0.724512	-0.938702
5	5	0	-1.306750	1.886258	0.562401
6	1	0	1.368076	-1.370807	-1.665861
7	1	0	2.418420	2.084976	0.147121
8	1	0	1.371352	-0.210016	1.432866
9	1	0	0.953863	0.870453	-2.420517
10	1	0	-1.200251	-1.384705	-1.754622
11	1	0	0.218082	3.297649	1.477759
12	1	0	-0.162502	3.170123	-1.597959
13	1	0	-1.057464	-1.111062	0.298754
14	1	0	-2.130308	1.135703	-1.809306

15	1	0	-2.302166	2.415745	0.948157
16	6	0	3.359026	-0.606580	0.263247
17	7	0	4.235984	-0.437341	1.274643
18	7	0	4.130750	-0.870695	-0.811932
19	1	0	3.945589	-0.209054	2.217857
20	1	0	3.750044	-1.023580	-1.737957
21	6	0	5.471148	-0.864520	-0.486648
22	6	0	5.538630	-0.589819	0.847849
23	1	0	6.248397	-1.049707	-1.211173
24	1	0	6.385487	-0.493052	1.508656
25	5	0	1.256683	1.830567	0.120798
26	5	0	-1.766317	-0.023639	0.392520
27	5	0	-1.284242	0.985534	-0.976697
28	6	0	0.112719	2.383221	0.896927
29	5	0	-0.149049	2.297941	-0.789883
30	6	0	-3.302891	-0.428019	0.288065
31	7	0	-4.112608	-0.815656	1.294906
32	1	0	-3.803211	-0.883372	2.255983
33	6	0	-5.392419	-1.083375	0.840912
34	1	0	-6.188241	-1.400778	1.495652
35	7	0	-4.084730	-0.457150	-0.805953
36	1	0	-3.737277	-0.167665	-1.713768
37	6	0	-5.371953	-0.855720	-0.501026
38	1	0	-6.148002	-0.936458	-1.245408
39	1	0	-1.443228	0.189791	1.539169

27a

Standard orientation:

Center Number	Atomic Number	Atomic Type	Coordinates (Angstroms) X	Y	Z
1	6	0	-0.881382	-1.180428	0.104351
2	5	0	-1.821167	-0.098077	-0.333246
3	5	0	-0.743788	0.221020	1.131176
4	5	0	0.636303	-1.096507	0.308676
5	5	0	1.339379	1.829811	-0.243095
6	1	0	-1.331570	-2.032866	0.615815
7	1	0	-2.363918	2.266898	-0.179877
8	1	0	-1.452880	0.666964	-1.270095
9	1	0	-1.218662	0.085831	2.212666
10	1	0	1.206894	-2.016902	0.819963
11	1	0	-0.138487	3.508449	-0.670433
12	1	0	-0.086165	2.497630	2.040519
13	1	0	1.199243	-0.940836	-1.013496
14	1	0	1.791199	0.400016	2.006406
15	1	0	2.339344	2.486859	-0.252206
16	6	0	-3.387567	-0.316684	-0.226172
17	7	0	-4.376036	0.585333	-0.076083
18	7	0	-4.021804	-1.506541	-0.291031
19	1	0	-4.187629	1.577046	0.018387
20	1	0	-3.532864	-2.382736	-0.423033
21	6	0	-5.391820	-1.357376	-0.178561

22	6	0	-5.616403	-0.021470	-0.041113
23	1	0	-6.072988	-2.192926	-0.206618
24	1	0	-6.532595	0.534842	0.077880
25	5	0	-1.335553	1.656367	-0.243644
26	5	0	1.835262	0.019449	-0.492211
27	5	0	1.116004	0.529849	1.034153
28	6	0	-0.044337	2.441337	-0.494425
29	5	0	0.030353	1.881162	1.030852
30	6	0	3.369646	-0.329629	-0.316104
31	7	0	4.412391	0.509010	-0.466064
32	1	0	4.295625	1.489574	-0.692367
33	6	0	5.613585	-0.128049	-0.200218
34	1	0	6.563918	0.377521	-0.262403
35	7	0	3.921003	-1.507352	0.033889
36	1	0	3.367320	-2.326607	0.252086
37	6	0	5.301183	-1.411874	0.118190
38	1	0	5.924832	-2.248842	0.388516
39	1	0	1.619110	0.796610	-1.425669

TS27a/p-2a

Standard orientation:

Center Number	Atomic Number	Atomic Type	Coordinates (Angstroms) X	Y	Z
1	6	0	-0.045920	2.299118	-1.086930
2	5	0	-1.776019	-0.224344	-0.546499
3	5	0	-0.496951	0.929970	1.242507
4	5	0	-1.206935	1.747038	-0.292007
5	5	0	0.113412	2.435084	0.610900
6	5	0	-0.739409	-0.844565	0.729857
7	5	0	1.683696	-0.001233	-0.285974
8	5	0	1.328013	1.215792	0.942063
9	5	0	1.374520	1.939706	-0.649221
10	6	0	0.698421	-0.356926	1.006157
11	1	0	-0.196909	3.161548	-1.734929
12	1	0	-1.568833	0.046879	-1.685859
13	1	0	-1.062231	0.942339	2.289534
14	1	0	-2.351649	2.086313	-0.273795
15	1	0	-0.011807	3.398932	1.295344
16	1	0	-1.292463	-1.567506	1.503293
17	1	0	1.209260	-0.302811	-1.341317
18	1	0	2.170979	1.380076	1.772728
19	1	0	2.346491	2.543898	-0.986817
20	1	0	1.097486	-0.805642	1.913460
21	6	0	3.197721	-0.472936	-0.199673
22	7	0	4.146544	-0.201476	0.720565
23	1	0	3.971096	0.413564	1.506247
24	6	0	5.353253	-0.798186	0.401895
25	1	0	6.234364	-0.692099	1.014510
26	7	0	3.826065	-1.253995	-1.104559
27	1	0	3.361858	-1.610824	-1.929509
28	6	0	5.150098	-1.469929	-0.763436

29	1	0	5.817911	-2.065358	-1.365270
30	6	0	-3.300722	-0.551841	-0.281144
31	7	0	-4.309606	-0.338474	-1.152189
32	1	0	-4.161912	0.031926	-2.083227
33	6	0	-5.532080	-0.664866	-0.601045
34	1	0	-6.461165	-0.559720	-1.138581
35	7	0	-3.905494	-1.019758	0.828791
36	1	0	-3.388527	-1.257754	1.667309
37	6	0	-5.272705	-1.104834	0.662946
38	1	0	-5.932577	-1.459917	1.438627
39	1	0	-0.973970	-1.252751	-0.500743

----

nhcx-iPR2 (Idip)

Standard orientation:

Center Number	Atomic Number	Atomic Type	Coordinates (Angstroms) X	Y	Z
1	6	0	0.337847	-0.041602	0.000000
2	7	0	-0.520182	1.033518	-0.000000
3	6	0	-1.866857	0.661824	-0.000000
4	1	0	-2.669875	1.383322	-0.000000
5	7	0	-0.545294	-1.096231	0.000000
6	6	0	-1.883151	-0.692693	0.000000
7	1	0	-2.702735	-1.395435	0.000000
8	6	0	-0.085488	2.406978	-0.000000
9	6	0	0.111871	3.060458	1.232716
10	6	0	0.111871	3.060458	-1.232716
11	6	0	0.506759	4.403706	1.204867
12	6	0	0.506759	4.403706	-1.204867
13	6	0	0.699964	5.072879	-0.000000
14	1	0	0.668218	4.930868	2.141268
15	1	0	0.668218	4.930868	-2.141268
16	1	0	1.006441	6.115775	-0.000000
17	6	0	-0.140428	-2.480091	0.000000
18	6	0	0.046367	-3.133478	-1.234446
19	6	0	0.046367	-3.133478	1.234446
20	6	0	0.427905	-4.480455	-1.206507
21	6	0	0.427905	-4.480455	1.206507
22	6	0	0.613941	-5.149262	0.000000
23	1	0	0.583919	-5.012819	-2.139606
24	1	0	0.583919	-5.012819	2.139606
25	1	0	0.908985	-6.195534	0.000000
26	6	0	-0.089758	-2.387298	-2.558094
27	6	0	-0.709683	-3.235142	-3.681473
28	6	0	1.279927	-1.816178	-2.981361
29	1	0	-0.758557	-1.535863	-2.392186
30	1	0	-1.664605	-3.677248	-3.375263
31	1	0	-0.892488	-2.608699	-4.562121
32	1	0	-0.047709	-4.049242	-3.998660
33	1	0	1.675771	-1.155813	-2.203354
34	1	0	2.002522	-2.624084	-3.150377
35	1	0	1.187806	-1.243793	-3.912870

36	6	0	-0.089758	-2.387298	2.558094
37	6	0	1.279927	-1.816178	2.981361
38	6	0	-0.709683	-3.235142	3.681473
39	1	0	-0.758557	-1.535863	2.392186
40	1	0	1.675771	-1.155813	2.203354
41	1	0	1.187806	-1.243793	3.912870
42	1	0	2.002522	-2.624084	3.150377
43	1	0	-1.664605	-3.677248	3.375263
44	1	0	-0.047709	-4.049242	3.998660
45	1	0	-0.892488	-2.608699	4.562121
46	6	0	-0.070621	2.353048	2.571362
47	6	0	-1.165050	3.019268	3.426719
48	6	0	1.262758	2.265616	3.338459
49	1	0	-0.391346	1.327779	2.368654
50	1	0	-2.123561	3.047956	2.896191
51	1	0	-1.310669	2.463289	4.360550
52	1	0	-0.900741	4.049620	3.692563
53	1	0	2.022029	1.751280	2.740809
54	1	0	1.647529	3.260492	3.592896
55	1	0	1.129308	1.710344	4.274717
56	6	0	-0.070621	2.353048	-2.571362
57	6	0	1.262758	2.265616	-3.338459
58	6	0	-1.165050	3.019268	-3.426719
59	1	0	-0.391346	1.327779	-2.368654
60	1	0	2.022029	1.751280	-2.740809
61	1	0	1.129308	1.710344	-4.274717
62	1	0	1.647529	3.260492	-3.592896
63	1	0	-2.123561	3.047956	-2.896191
64	1	0	-0.900741	4.049620	-3.692563
65	1	0	-1.310669	2.463289	-4.360550

----

full(o-1)

Standard orientation:

Center Number	Atomic Number	Atomic Type	Coordinates (Angstroms) X	Y	Z
1	7	0	-1.177796	-0.138116	-1.022429
2	1	0	1.295070	1.325296	5.448399
3	7	0	0.985430	-0.072331	-1.158956
4	1	0	1.659521	-1.762633	4.210119
5	6	0	-2.570637	-0.183117	-0.606756
6	1	0	3.078113	0.663395	3.347749
7	6	0	-0.047247	0.012516	-0.260089
8	1	0	2.162965	-0.731400	1.553254
9	6	0	2.409376	0.019075	-0.906429
10	1	0	-1.127347	2.696947	1.267929
11	6	0	-3.112014	-1.424144	-0.213379
12	1	0	0.138958	2.306199	2.820005
13	6	0	-0.843677	-0.309479	-2.361027
14	1	0	-1.602843	-0.451228	-3.112167
15	1	0	-1.293197	2.365912	4.279337
16	6	0	-3.349009	0.986565	-0.726559

17	1	0	-2.409497	0.035336	2.339728
18	6	0	3.155527	-1.177962	-0.870527
19	1	0	-1.109691	-0.501606	4.936924
20	6	0	3.000590	1.291491	-0.783472
21	6	0	-2.291522	-2.711019	-0.173387
22	1	0	-1.234168	-2.438046	-0.107830
23	6	0	-4.475921	-1.459134	0.097670
24	1	0	-4.925918	-2.394911	0.412379
25	6	0	0.503519	-0.266444	-2.447940
26	1	0	1.171105	-0.353418	-3.289631
27	6	0	4.540072	-1.067677	-0.694963
28	1	0	5.145186	-1.968774	-0.659560
29	6	0	2.204375	2.589573	-0.874464
30	1	0	1.140382	2.342478	-0.836559
31	6	0	-4.707157	0.887720	-0.400802
32	1	0	-5.336341	1.768797	-0.472184
33	6	0	5.152283	0.175094	-0.561269
34	1	0	6.228252	0.237465	-0.422956
35	6	0	-2.789517	2.310963	-1.242624
36	1	0	-1.702028	2.288958	-1.121349
37	6	0	1.443023	-0.171385	2.139651
38	6	0	2.521750	-2.560103	-1.021193
39	1	0	1.434427	-2.438431	-1.045157
40	6	0	1.996499	0.586350	3.278054
41	6	0	-5.265255	-0.317114	0.011955
42	1	0	-6.321233	-0.367649	0.263399
43	6	0	4.389153	1.337674	-0.605813
44	1	0	4.878497	2.301593	-0.505197
45	6	0	2.472485	3.298588	-2.217841
46	1	0	1.870572	4.211595	-2.293103
47	1	0	2.221491	2.655997	-3.069743
48	1	0	3.527038	3.583039	-2.314130
49	6	0	2.481812	3.527671	0.314299
50	1	0	2.289037	3.023469	1.265918
51	1	0	1.827693	4.404677	0.256405
52	1	0	3.516884	3.888968	0.318698
53	6	0	-2.490273	-3.520664	-1.472813
54	1	0	-1.883821	-4.433958	-1.451115
55	1	0	-2.202412	-2.946211	-2.360418
56	1	0	-3.539464	-3.816074	-1.592167
57	6	0	-2.601199	-3.583367	1.056024
58	1	0	-2.512077	-3.010111	1.982527
59	1	0	-1.893180	-4.418372	1.103927
60	1	0	-3.607801	-4.014886	1.009960
61	5	0	0.072735	0.374868	1.279144
62	6	0	-3.097443	2.488342	-2.745406
63	1	0	-4.179505	2.507234	-2.921440
64	1	0	-2.675973	1.680149	-3.352478
65	1	0	-2.680322	3.434137	-3.110942
66	1	0	-0.267044	-2.061793	2.158038
67	6	0	-3.306942	3.529317	-0.456039
68	1	0	-3.158595	3.403354	0.619321
69	1	0	-4.371873	3.712279	-0.640296

70	1	0	-2.765923	4.428628	-0.771744
71	6	0	2.940302	-3.218439	-2.351635
72	1	0	4.022686	-3.387939	-2.387960
73	1	0	2.673533	-2.595629	-3.213085
74	1	0	2.445349	-4.189251	-2.469420
75	5	0	-0.049706	-0.938463	2.488787
76	5	0	-1.267571	0.328747	2.504550
77	5	0	-0.599522	1.802732	1.849289
78	5	0	-0.694614	1.613929	3.576341
79	5	0	-0.431834	-0.084265	4.048804
80	6	0	2.844096	-3.483611	0.169841
81	1	0	2.510924	-3.049972	1.117969
82	1	0	3.918237	-3.685977	0.248524
83	1	0	2.335909	-4.446440	0.043758
84	5	0	1.004820	0.913420	4.368898
85	5	0	1.168230	-0.781317	3.753718

----

full(o-2)

Standard orientation:

Center Number	Atomic Number	Atomic Type	Coordinates (Angstroms) X	Y	Z
1	5	0	0.102958	-0.418962	1.533453
2	5	0	1.225127	-1.817601	1.404628
3	5	0	-0.372808	-2.197589	-0.844417
4	5	0	1.087746	-2.830658	0.000000
5	5	0	0.102958	-0.418962	-1.533453
6	5	0	2.280627	-1.536426	0.000000
7	5	0	1.225127	-1.817601	-1.404628
8	6	0	1.533685	-0.203234	-0.766362
9	1	0	-1.054009	-2.889524	1.545531
10	1	0	-0.826117	0.056558	0.949718
11	1	0	1.756749	-2.098358	2.427131
12	1	0	-1.054009	-2.889524	-1.545531
13	1	0	1.461198	-3.967195	0.000000
14	1	0	2.313862	0.405436	1.211248
15	1	0	-0.826117	0.056558	-0.949718
16	1	0	3.468742	-1.608214	0.000000
17	1	0	1.756749	-2.098358	-2.427131
18	1	0	2.313862	0.405436	-1.211248
19	6	0	-0.049007	0.039176	-3.065798
20	7	0	0.854373	0.260619	-4.082126
21	6	0	0.212609	0.763016	-5.210826
22	1	0	0.760985	0.998115	-6.107989
23	7	0	-1.257268	0.411637	-3.610408
24	6	0	-1.102406	0.850712	-4.917389
25	1	0	-1.943701	1.177638	-5.505612
26	6	0	-0.049007	0.039176	3.065798
27	7	0	0.854373	0.260619	4.082126
28	6	0	0.212609	0.763016	5.210826
29	1	0	0.760985	0.998115	6.107989
30	7	0	-1.257268	0.411637	3.610408

31	6	0	-1.102406	0.850712	4.917389
32	1	0	-1.943701	1.177638	5.505612
33	6	0	1.533685	-0.203234	0.766362
34	5	0	-0.372808	-2.197589	0.844417
35	6	0	2.284545	0.004520	4.146601
36	6	0	3.180323	1.021521	3.756491
37	6	0	2.719064	-1.203623	4.733274
38	6	0	4.548633	0.780064	3.931428
39	6	0	4.098156	-1.385271	4.887585
40	6	0	5.005409	-0.410528	4.486412
41	1	0	5.265839	1.538198	3.633969
42	1	0	4.465658	-2.307483	5.326287
43	1	0	6.072071	-0.577129	4.611241
44	6	0	2.284545	0.004520	-4.146601
45	6	0	2.719064	-1.203623	-4.733274
46	6	0	3.180323	1.021521	-3.756491
47	6	0	4.098156	-1.385271	-4.887585
48	6	0	4.548633	0.780064	-3.931428
49	6	0	5.005409	-0.410528	-4.486412
50	1	0	4.465658	-2.307483	-5.326287
51	1	0	5.265839	1.538198	-3.633969
52	1	0	6.072071	-0.577129	-4.611241
53	6	0	-2.574980	0.343329	3.004767
54	6	0	-3.137661	1.501435	2.428494
55	6	0	-3.293267	-0.870359	3.119225
56	6	0	-4.440313	1.391653	1.919625
57	6	0	-4.590367	-0.908788	2.596521
58	6	0	-5.160031	0.207118	1.994715
59	1	0	-4.893655	2.267063	1.461026
60	1	0	-5.161204	-1.828361	2.660155
61	1	0	-6.166551	0.153184	1.588286
62	6	0	-2.574980	0.343329	-3.004767
63	6	0	-3.293267	-0.870359	-3.119225
64	6	0	-3.137661	1.501435	-2.428494
65	6	0	-4.590367	-0.908788	-2.596521
66	6	0	-4.440313	1.391653	-1.919625
67	6	0	-5.160031	0.207118	-1.994715
68	1	0	-5.161204	-1.828361	-2.660155
69	1	0	-4.893655	2.267063	-1.461026
70	1	0	-6.166551	0.153184	-1.588286
71	6	0	-2.739517	-2.093179	3.850987
72	6	0	-3.228652	-3.429502	3.265844
73	6	0	-3.086114	-2.031640	5.355508
74	1	0	-1.651173	-2.084624	3.745131
75	1	0	-3.065170	-3.473984	2.186771
76	1	0	-2.668538	-4.252431	3.723317
77	1	0	-4.290804	-3.605931	3.476133
78	1	0	-2.679642	-1.139450	5.842031
79	1	0	-4.173097	-2.029238	5.502878
80	1	0	-2.679181	-2.908458	5.873287
81	6	0	-2.739517	-2.093179	-3.850987
82	6	0	-3.086114	-2.031640	-5.355508
83	6	0	-3.228652	-3.429502	-3.265844

84	1	0	-1.651173	-2.084624	-3.745131
85	1	0	-2.679642	-1.139450	-5.842031
86	1	0	-2.679181	-2.908458	-5.873287
87	1	0	-4.173097	-2.029238	-5.502878
88	1	0	-3.065170	-3.473984	-2.186771
89	1	0	-4.290804	-3.605931	-3.476133
90	1	0	-2.668538	-4.252431	-3.723317
91	6	0	-2.530182	2.910149	2.367074
92	6	0	-1.021739	3.044599	2.111310
93	6	0	-2.944073	3.736127	3.605239
94	1	0	-3.024697	3.379889	1.506332
95	1	0	-0.680519	2.371766	1.320562
96	1	0	-0.803128	4.076068	1.808837
97	1	0	-0.435995	2.844795	3.012953
98	1	0	-4.028516	3.715950	3.757783
99	1	0	-2.466886	3.353981	4.514819
100	1	0	-2.634794	4.781565	3.485083
101	6	0	1.755549	-2.275152	-5.240725
102	6	0	2.161438	-3.700072	-4.820613
103	6	0	1.611987	-2.194443	-6.775846
104	1	0	0.773102	-2.081569	-4.800310
105	1	0	2.284984	-3.774378	-3.737406
106	1	0	1.381346	-4.407764	-5.123682
107	1	0	3.093490	-4.019319	-5.301691
108	1	0	1.256442	-1.212049	-7.105136
109	1	0	2.572694	-2.383942	-7.269534
110	1	0	0.897671	-2.946462	-7.131142
111	6	0	2.706465	2.373354	-3.227097
112	6	0	2.611170	3.403881	-4.372772
113	6	0	3.586384	2.930205	-2.093775
114	1	0	1.702970	2.238832	-2.815197
115	1	0	1.926017	3.074687	-5.160849
116	1	0	2.248460	4.366650	-3.993225
117	1	0	3.594048	3.569038	-4.830166
118	1	0	3.722631	2.199979	-1.290152
119	1	0	4.578514	3.229113	-2.451221
120	1	0	3.115176	3.821921	-1.665153
121	6	0	1.755549	-2.275152	5.240725
122	6	0	1.611987	-2.194443	6.775846
123	6	0	2.161438	-3.700072	4.820613
124	1	0	0.773102	-2.081569	4.800310
125	1	0	1.256442	-1.212049	7.105136
126	1	0	0.897671	-2.946462	7.131142
127	1	0	2.572694	-2.383942	7.269534
128	1	0	2.284984	-3.774378	3.737406
129	1	0	3.093490	-4.019319	5.301691
130	1	0	1.381346	-4.407764	5.123682
131	6	0	2.706465	2.373354	3.227097
132	6	0	3.586384	2.930205	2.093775
133	6	0	2.611170	3.403881	4.372772
134	1	0	1.702970	2.238832	2.815197
135	1	0	3.722631	2.199979	1.290152
136	1	0	3.115176	3.821921	1.665153

137	1	0	4.578514	3.229113	2.451221
138	1	0	1.926017	3.074687	5.160849
139	1	0	3.594048	3.569038	4.830166
140	1	0	2.248460	4.366650	3.993225
141	6	0	-2.530182	2.910149	-2.367074
142	6	0	-2.944073	3.736127	-3.605239
143	6	0	-1.021739	3.044599	-2.111310
144	1	0	-3.024697	3.379889	-1.506332
145	1	0	-4.028516	3.715950	-3.757783
146	1	0	-2.634794	4.781565	-3.485083
147	1	0	-2.466886	3.353981	-4.514819
148	1	0	-0.680519	2.371766	-1.320562
149	1	0	-0.435995	2.844795	-3.012953
150	1	0	-0.803128	4.076068	-1.808837

----

full(o-2)

Standard orientation:

Center Number	Atomic Number	Atomic Type	Coordinates (Angstroms) X	Y	Z
1	5	0	1.552962	0.039730	-0.348874
2	5	0	1.530594	1.069554	-1.840221
3	5	0	-0.745234	-0.499562	-2.137712
4	5	0	0.157344	0.866187	-2.884584
5	5	0	-1.500333	0.198799	-0.442950
6	5	0	0.154547	2.179447	-1.709607
7	5	0	-1.282623	1.142881	-1.944164
8	6	0	-0.662688	1.588025	-0.323189
9	1	0	1.636385	-1.313509	-2.697436
10	1	0	0.874303	-0.723789	0.272627
11	1	0	2.570712	1.548365	-2.142324
12	1	0	-1.425772	-1.268148	-2.748408
13	1	0	0.203525	1.129064	-4.050691
14	1	0	1.331352	2.350961	0.262399
15	1	0	-0.991741	-0.758654	0.064440
16	1	0	0.199609	3.354825	-1.897083
17	1	0	-2.282021	1.675108	-2.319668
18	1	0	-1.078368	2.446443	0.192426
19	6	0	-3.059408	0.182686	-0.045744
20	7	0	-3.860980	1.154281	0.506146
21	6	0	-5.118523	0.641813	0.814590
22	1	0	-5.878952	1.257493	1.266398
23	7	0	-3.862620	-0.931155	-0.080083
24	6	0	-5.118418	-0.656931	0.443747
25	1	0	-5.884331	-1.413187	0.493621
26	6	0	3.049571	-0.239979	0.175451
27	7	0	4.280621	0.351413	0.002562
28	6	0	5.265957	-0.344957	0.695369
29	1	0	6.296619	-0.031188	0.665665
30	7	0	3.314636	-1.313891	0.998030
31	6	0	4.664121	-1.388148	1.306902
32	1	0	5.059018	-2.177478	1.924929

33	6	0	0.869942	1.542188	-0.297291
34	5	0	0.944327	-0.543723	-2.084661
35	6	0	4.693557	1.468895	-0.831334
36	6	0	4.675418	2.771124	-0.295922
37	6	0	5.220751	1.178369	-2.105900
38	6	0	5.192521	3.801749	-1.090061
39	6	0	5.727637	2.247918	-2.852031
40	6	0	5.713250	3.546689	-2.354478
41	1	0	5.189267	4.818772	-0.710859
42	1	0	6.132095	2.058573	-3.841877
43	1	0	6.107937	4.362898	-2.953741
44	6	0	-3.565541	2.546158	0.791898
45	6	0	-3.962103	3.527456	-0.141019
46	6	0	-2.999667	2.874595	2.041257
47	6	0	-3.745294	4.867769	0.203921
48	6	0	-2.809135	4.230630	2.330586
49	6	0	-3.172960	5.219167	1.421594
50	1	0	-4.033001	5.647351	-0.494205
51	1	0	-2.372817	4.514887	3.283446
52	1	0	-3.014316	6.266431	1.664948
53	6	0	2.397276	-2.320370	1.503103
54	6	0	1.922245	-2.223481	2.828194
55	6	0	2.116090	-3.434034	0.679640
56	6	0	1.131425	-3.280799	3.302629
57	6	0	1.329314	-4.458784	1.216818
58	6	0	0.839511	-4.388576	2.516457
59	1	0	0.747996	-3.227163	4.318859
60	1	0	1.100171	-5.324980	0.605255
61	1	0	0.233552	-5.197344	2.917167
62	6	0	-3.563740	-2.216848	-0.683062
63	6	0	-3.833337	-2.364158	-2.063987
64	6	0	-3.103743	-3.281822	0.116695
65	6	0	-3.572778	-3.607027	-2.647797
66	6	0	-2.839235	-4.495667	-0.537014
67	6	0	-3.060654	-4.660327	-1.897933
68	1	0	-3.759141	-3.746659	-3.707178
69	1	0	-2.462784	-5.329690	0.050337
70	1	0	-2.843016	-5.612810	-2.373962
71	6	0	2.680801	-3.581357	-0.730869
72	6	0	1.660538	-4.154904	-1.729229
73	6	0	3.965634	-4.437015	-0.709904
74	1	0	2.939247	-2.586458	-1.100143
75	1	0	0.742284	-3.562138	-1.736611
76	1	0	2.083300	-4.120892	-2.739051
77	1	0	1.413367	-5.201873	-1.513385
78	1	0	4.736234	-4.004437	-0.061450
79	1	0	3.756576	-5.451784	-0.349223
80	1	0	4.383665	-4.518851	-1.720176
81	6	0	-4.469487	-1.253372	-2.900325
82	6	0	-6.004683	-1.425056	-2.932073
83	6	0	-3.923450	-1.162537	-4.336035
84	1	0	-4.244929	-0.296440	-2.420634
85	1	0	-6.443926	-1.419705	-1.928787

86	1	0	-6.467230	-0.612818	-3.505550
87	1	0	-6.278657	-2.372948	-3.410938
88	1	0	-2.835288	-1.068103	-4.336320
89	1	0	-4.205554	-2.033342	-4.940127
90	1	0	-4.345340	-0.279307	-4.829355
91	6	0	2.215486	-1.114363	3.847422
92	6	0	2.314883	0.327365	3.326402
93	6	0	3.442806	-1.464773	4.717025
94	1	0	1.348637	-1.132916	4.521291
95	1	0	1.533262	0.550164	2.595457
96	1	0	2.206233	1.020396	4.169360
97	1	0	3.286498	0.530457	2.867923
98	1	0	3.352230	-2.464583	5.155164
99	1	0	4.368027	-1.433366	4.131005
100	1	0	3.546109	-0.741618	5.535085
101	6	0	-4.655004	3.192551	-1.459530
102	6	0	-4.070990	3.968642	-2.654133
103	6	0	-6.175622	3.435215	-1.351906
104	1	0	-4.498394	2.129587	-1.662610
105	1	0	-2.989905	3.826025	-2.729513
106	1	0	-4.525389	3.609058	-3.584306
107	1	0	-4.277897	5.043163	-2.585766
108	1	0	-6.630085	2.839073	-0.552826
109	1	0	-6.391878	4.490232	-1.145045
110	1	0	-6.670733	3.168770	-2.293052
111	6	0	-2.630866	1.820652	3.079525
112	6	0	-3.664737	1.795439	4.223935
113	6	0	-1.206575	2.009179	3.630618
114	1	0	-2.652647	0.843722	2.592138
115	1	0	-4.675402	1.598153	3.849033
116	1	0	-3.414742	1.011824	4.948976
117	1	0	-3.688020	2.753353	4.757270
118	1	0	-0.471815	2.031464	2.820559
119	1	0	-1.107592	2.934689	4.209983
120	1	0	-0.952699	1.177224	4.297689
121	6	0	5.255941	-0.229778	-2.693946
122	6	0	6.705189	-0.745218	-2.807007
123	6	0	4.533034	-0.306547	-4.052211
124	1	0	4.720632	-0.899084	-2.014678
125	1	0	7.213895	-0.745512	-1.835734
126	1	0	6.715019	-1.770859	-3.194050
127	1	0	7.296311	-0.124784	-3.491118
128	1	0	3.490812	0.009187	-3.960053
129	1	0	5.023874	0.316852	-4.809013
130	1	0	4.542755	-1.339797	-4.418502
131	6	0	4.161649	3.076218	1.107750
132	6	0	3.300875	4.351466	1.168614
133	6	0	5.329886	3.166524	2.111928
134	1	0	3.523522	2.244668	1.418472
135	1	0	2.500947	4.332368	0.421750
136	1	0	2.843072	4.443818	2.160344
137	1	0	3.896082	5.256722	1.002116
138	1	0	5.910814	2.238070	2.141244

139	1	0	6.013841	3.980532	1.843285
140	1	0	4.953985	3.360925	3.123644
141	6	0	-2.968088	-3.321508	1.645460
142	6	0	-4.271330	-3.867817	2.271760
143	6	0	-2.513381	-2.057672	2.388959
144	1	0	-2.190630	-4.073469	1.831767
145	1	0	-4.569388	-4.818868	1.817842
146	1	0	-4.138807	-4.028274	3.348743
147	1	0	-5.098928	-3.159828	2.142981
148	1	0	-1.641902	-1.597628	1.917709
149	1	0	-3.311784	-1.312674	2.458598
150	1	0	-2.240688	-2.333748	3.414864

----

full(m-2) nhcx-x40b

Standard orientation:

Center Number	Atomic Number	Atomic Type	Coordinates (Angstroms) X	Y	Z
1	6	0	-1.157061	1.018766	1.278811
2	5	0	-0.170109	1.545751	0.000000
3	5	0	-2.673140	0.336866	0.898530
4	5	0	-1.244451	-0.605635	1.404162
5	5	0	-1.244451	-0.605635	-1.404162
6	1	0	-1.149512	1.585723	2.207560
7	1	0	-2.491250	2.743913	0.000000
8	1	0	0.850578	0.910563	0.000000
9	1	0	-3.629497	0.525048	1.587769
10	1	0	-1.318604	-1.047636	2.512379
11	1	0	-1.149512	1.585723	-2.207560
12	1	0	-3.629497	0.525048	-1.587769
13	1	0	0.567973	-0.988669	-0.000000
14	1	0	-2.934608	-2.085992	-0.000000
15	1	0	-1.318604	-1.047636	-2.512379
16	6	0	0.391437	3.106102	0.000000
17	7	0	1.748110	3.324916	0.000000
18	7	0	-0.135369	4.381259	0.000000
19	6	0	0.874119	5.336046	0.000000
20	6	0	2.051696	4.675198	0.000000
21	1	0	0.644747	6.389146	0.000000
22	1	0	3.071403	5.024668	0.000000
23	5	0	-2.029853	1.660583	0.000000
24	5	0	-0.516391	-1.511081	-0.000000
25	5	0	-2.231690	-1.113969	-0.000000
26	6	0	-1.157061	1.018766	-1.278811
27	5	0	-2.673140	0.336866	-0.898530
28	6	0	-0.427632	-3.130089	-0.000000
29	7	0	-0.399742	-3.979239	1.078870
30	6	0	-0.361220	-5.307902	0.675241
31	1	0	-0.331141	-6.112253	1.392123
32	7	0	-0.399742	-3.979239	-1.078870
33	6	0	-0.361220	-5.307902	-0.675241
34	1	0	-0.331141	-6.112253	-1.392123

35	6	0	2.823910	2.355064	0.000000
36	6	0	3.364155	1.957978	-1.237461
37	6	0	3.364155	1.957978	1.237461
38	6	0	4.465981	1.095327	-1.206965
39	6	0	4.465981	1.095327	1.206965
40	6	0	5.009464	0.663349	0.000000
41	1	0	4.904872	0.760073	-2.142246
42	1	0	4.904872	0.760073	2.142246
43	1	0	5.866285	-0.005331	0.000000
44	6	0	-1.501935	4.881978	0.000000
45	6	0	-2.103937	5.184555	-1.236101
46	6	0	-2.103937	5.184555	1.236101
47	6	0	-3.358458	5.804084	-1.205068
48	6	0	-3.358458	5.804084	1.205068
49	6	0	-3.981501	6.112856	0.000000
50	1	0	-3.854757	6.045423	-2.140241
51	1	0	-3.854757	6.045423	2.140241
52	1	0	-4.956634	6.592397	0.000000
53	6	0	-0.305816	-3.660888	-2.489677
54	6	0	-1.477121	-3.674906	-3.271052
55	6	0	0.974903	-3.470433	-3.040734
56	6	0	-1.333923	-3.450022	-4.644694
57	6	0	1.056314	-3.230155	-4.417692
58	6	0	-0.085086	-3.216000	-5.212746
59	1	0	-2.216759	-3.448463	-5.276669
60	1	0	2.029241	-3.066311	-4.872788
61	1	0	-0.000678	-3.032265	-6.280811
62	6	0	-0.305816	-3.660888	2.489677
63	6	0	-1.477121	-3.674906	3.271052
64	6	0	0.974903	-3.470433	3.040734
65	6	0	-1.333923	-3.450022	4.644694
66	6	0	1.056314	-3.230155	4.417692
67	6	0	-0.085086	-3.216000	5.212746
68	1	0	-2.216759	-3.448463	5.276669
69	1	0	2.029241	-3.066311	4.872788
70	1	0	-0.000678	-3.032265	6.280811
71	6	0	-1.449200	4.867582	2.577303
72	6	0	-2.385938	4.069241	3.504076
73	6	0	-0.953112	6.152440	3.271158
74	1	0	-0.575162	4.236703	2.387590
75	1	0	-2.752266	3.162410	3.014068
76	1	0	-1.849413	3.774881	4.413645
77	1	0	-3.253601	4.663121	3.813967
78	1	0	-0.234091	6.696877	2.648318
79	1	0	-1.786580	6.830955	3.488485
80	1	0	-0.462016	5.909863	4.220946
81	6	0	-1.449200	4.867582	-2.577303
82	6	0	-0.953112	6.152440	-3.271158
83	6	0	-2.385938	4.069241	-3.504076
84	1	0	-0.575162	4.236703	-2.387590
85	1	0	-0.234091	6.696877	-2.648318
86	1	0	-0.462016	5.909863	-4.220946
87	1	0	-1.786580	6.830955	-3.488485

88	1	0	-2.752266	3.162410	-3.014068
89	1	0	-3.253601	4.663121	-3.813967
90	1	0	-1.849413	3.774881	-4.413645
91	6	0	2.254234	-3.566451	-2.214896
92	6	0	3.051399	-2.251813	-2.227888
93	6	0	3.124719	-4.752510	-2.676350
94	1	0	1.976165	-3.757486	-1.174840
95	1	0	2.445564	-1.426436	-1.845372
96	1	0	3.943407	-2.342564	-1.596309
97	1	0	3.385128	-1.994927	-3.240589
98	1	0	2.572790	-5.698173	-2.627458
99	1	0	3.467704	-4.621338	-3.709317
100	1	0	4.012808	-4.844482	-2.039562
101	6	0	-2.856387	-3.958552	-2.683456
102	6	0	-3.310308	-5.392018	-3.030211
103	6	0	-3.913366	-2.929809	-3.123508
104	1	0	-2.781013	-3.883602	-1.595285
105	1	0	-2.601704	-6.145133	-2.665858
106	1	0	-4.286967	-5.602583	-2.578573
107	1	0	-3.406373	-5.524370	-4.114806
108	1	0	-3.597681	-1.914542	-2.871460
109	1	0	-4.110299	-2.980215	-4.201429
110	1	0	-4.859861	-3.131538	-2.608684
111	6	0	2.254234	-3.566451	2.214896
112	6	0	3.124719	-4.752510	2.676350
113	6	0	3.051399	-2.251813	2.227888
114	1	0	1.976165	-3.757486	1.174840
115	1	0	2.572790	-5.698173	2.627458
116	1	0	4.012808	-4.844482	2.039562
117	1	0	3.467704	-4.621338	3.709317
118	1	0	2.445564	-1.426436	1.845372
119	1	0	3.385128	-1.994927	3.240589
120	1	0	3.943407	-2.342564	1.596309
121	6	0	-2.856387	-3.958552	2.683456
122	6	0	-3.913366	-2.929809	3.123508
123	6	0	-3.310308	-5.392018	3.030211
124	1	0	-2.781013	-3.883602	1.595285
125	1	0	-3.597681	-1.914542	2.871460
126	1	0	-4.859861	-3.131538	2.608684
127	1	0	-4.110299	-2.980215	4.201429
128	1	0	-2.601704	-6.145133	2.665858
129	1	0	-3.406373	-5.524370	4.114806
130	1	0	-4.286967	-5.602583	2.578573
131	6	0	2.805156	2.441000	2.573009
132	6	0	3.862484	3.228811	3.371715
133	6	0	2.224859	1.284456	3.408133
134	1	0	1.980137	3.129358	2.366343
135	1	0	4.251886	4.077833	2.797878
136	1	0	3.425558	3.617412	4.298988
137	1	0	4.713808	2.595523	3.647178
138	1	0	1.439456	0.753116	2.862432
139	1	0	3.001559	0.561313	3.681761
140	1	0	1.792003	1.673835	4.337510

141	6	0	2.805156	2.441000	-2.573009
142	6	0	2.224859	1.284456	-3.408133
143	6	0	3.862484	3.228811	-3.371715
144	1	0	1.980137	3.129358	-2.366343
145	1	0	1.439456	0.753116	-2.862432
146	1	0	1.792003	1.673835	-4.337510
147	1	0	3.001559	0.561313	-3.681761
148	1	0	4.251886	4.077833	-2.797878
149	1	0	4.713808	2.595523	-3.647178
150	1	0	3.425558	3.617412	-4.298988

----

full(m-2)

Standard orientation:

Center Number	Atomic Number	Atomic Type	Coordinates (Angstroms) X	Y	Z
1	6	0	-1.302474	-0.735291	1.273184
2	5	0	-0.439062	-1.447617	-0.000000
3	5	0	-2.682845	0.198906	0.895100
4	5	0	-1.115768	0.881132	1.402399
5	5	0	-1.115768	0.881132	-1.402399
6	1	0	-1.395200	-1.274754	2.208911
7	1	0	-2.914387	-2.239356	-0.000000
8	1	0	0.725911	-1.098807	-0.000000
9	1	0	-3.649123	0.189683	1.595455
10	1	0	-1.081703	1.357655	2.503667
11	1	0	-1.395200	-1.274754	-2.208911
12	1	0	-3.649123	0.189683	-1.595455
13	1	0	0.672501	0.856932	0.000000
14	1	0	-2.545353	2.598200	0.000000
15	1	0	-1.081703	1.357655	-2.503667
16	6	0	-0.405332	-3.112094	-0.000000
17	7	0	-0.350388	-3.952919	-1.079160
18	7	0	-0.350388	-3.952919	1.079160
19	6	0	-0.269758	-5.281018	0.675200
20	6	0	-0.269758	-5.281018	-0.675200
21	1	0	-0.214321	-6.083570	1.392582
22	1	0	-0.214321	-6.083570	-1.392582
23	5	0	-2.291678	-1.222107	-0.000000
24	5	0	-0.235901	1.638178	0.000000
25	5	0	-1.995924	1.548661	0.000000
26	6	0	-1.302474	-0.735291	-1.273184
27	5	0	-2.682845	0.198906	-0.895100
28	6	0	0.356686	3.108075	0.000000
29	7	0	1.713266	3.360048	0.000000
30	6	0	1.983525	4.721744	0.000000
31	1	0	2.994180	5.096720	0.000000
32	7	0	-0.198937	4.373788	0.000000
33	6	0	0.791731	5.353016	0.000000
34	1	0	0.537575	6.400309	0.000000
35	6	0	-0.262966	-3.617106	-2.487976
36	6	0	-1.433293	-3.655570	-3.273069

37	6	0	1.012524	-3.369257	-3.030508
38	6	0	-1.297047	-3.377246	-4.638012
39	6	0	1.085257	-3.086569	-4.400173
40	6	0	-0.055968	-3.081679	-5.195181
41	1	0	-2.177978	-3.388216	-5.272233
42	1	0	2.052072	-2.878083	-4.848988
43	1	0	0.022349	-2.858173	-6.255778
44	6	0	-0.262966	-3.617106	2.487976
45	6	0	-1.433293	-3.655570	3.273069
46	6	0	1.012524	-3.369257	3.030508
47	6	0	-1.297047	-3.377246	4.638012
48	6	0	1.085257	-3.086569	4.400173
49	6	0	-0.055968	-3.081679	5.195181
50	1	0	-2.177978	-3.388216	5.272233
51	1	0	2.052072	-2.878083	4.848988
52	1	0	0.022349	-2.858173	6.255778
53	6	0	-1.579165	4.822666	0.000000
54	6	0	-2.194882	5.100711	1.235537
55	6	0	-2.194882	5.100711	-1.235537
56	6	0	-3.472158	5.672011	1.205034
57	6	0	-3.472158	5.672011	-1.205034
58	6	0	-4.106537	5.957001	0.000000
59	1	0	-3.977115	5.894743	2.140265
60	1	0	-3.977115	5.894743	-2.140265
61	1	0	-5.099168	6.399911	0.000000
62	6	0	2.790907	2.402723	0.000000
63	6	0	3.333654	2.003342	1.236679
64	6	0	3.333654	2.003342	-1.236679
65	6	0	4.445020	1.152347	1.206977
66	6	0	4.445020	1.152347	-1.206977
67	6	0	4.994172	0.727323	0.000000
68	1	0	4.891061	0.826835	2.142555
69	1	0	4.891061	0.826835	-2.142555
70	1	0	5.861463	0.071748	0.000000
71	6	0	2.787255	2.505383	2.570544
72	6	0	2.341480	1.358542	3.494559
73	6	0	3.811518	3.419208	3.273625
74	1	0	1.898021	3.107532	2.365930
75	1	0	1.561905	0.755368	3.021036
76	1	0	1.931423	1.768288	4.425264
77	1	0	3.179468	0.703664	3.761621
78	1	0	4.095954	4.267493	2.640296
79	1	0	4.727596	2.872732	3.528195
80	1	0	3.390917	3.817594	4.204437
81	6	0	2.787255	2.505383	-2.570544
82	6	0	3.811518	3.419208	-3.273625
83	6	0	2.341480	1.358542	-3.494559
84	1	0	1.898021	3.107532	-2.365930
85	1	0	4.095954	4.267493	-2.640296
86	1	0	3.390917	3.817594	-4.204437
87	1	0	4.727596	2.872732	-3.528195
88	1	0	1.561905	0.755368	-3.021036
89	1	0	3.179468	0.703664	-3.761621

90	1	0	1.931423	1.768288	-4.425264
91	6	0	-1.523368	4.814799	2.575459
92	6	0	-1.045096	6.120484	3.242965
93	6	0	-2.433656	4.012983	3.523654
94	1	0	-0.642638	4.194107	2.386581
95	1	0	-0.342405	6.668077	2.604325
96	1	0	-0.540137	5.903407	4.191970
97	1	0	-1.889400	6.786959	3.457283
98	1	0	-2.758015	3.079257	3.056897
99	1	0	-3.321361	4.584816	3.819390
100	1	0	-1.885486	3.761107	4.439182
101	6	0	-1.523368	4.814799	-2.575459
102	6	0	-2.433656	4.012983	-3.523654
103	6	0	-1.045096	6.120484	-3.242965
104	1	0	-0.642638	4.194107	-2.386581
105	1	0	-2.758015	3.079257	-3.056897
106	1	0	-1.885486	3.761107	-4.439182
107	1	0	-3.321361	4.584816	-3.819390
108	1	0	-0.342405	6.668077	-2.604325
109	1	0	-1.889400	6.786959	-3.457283
110	1	0	-0.540137	5.903407	-4.191970
111	6	0	-2.799568	-4.027770	-2.702586
112	6	0	-3.175759	-5.469619	-3.104916
113	6	0	-3.910524	-3.043548	-3.111628
114	1	0	-2.731760	-3.992881	-1.611658
115	1	0	-2.426727	-6.196467	-2.769604
116	1	0	-4.138918	-5.749445	-2.662515
117	1	0	-3.265589	-5.564630	-4.193653
118	1	0	-3.669805	-2.019767	-2.812944
119	1	0	-4.091393	-3.056775	-4.192941
120	1	0	-4.849195	-3.324276	-2.620570
121	6	0	2.295333	-3.462318	-2.209636
122	6	0	3.107544	-2.157665	-2.238499
123	6	0	3.149644	-4.661601	-2.669378
124	1	0	2.022330	-3.641934	-1.166202
125	1	0	2.519079	-1.319991	-1.856137
126	1	0	4.001381	-2.257229	-1.611550
127	1	0	3.440351	-1.910825	-3.253562
128	1	0	2.590341	-5.602222	-2.607229
129	1	0	3.482321	-4.540902	-3.706918
130	1	0	4.043161	-4.755672	-2.040950
131	6	0	2.295333	-3.462318	2.209636
132	6	0	3.149644	-4.661601	2.669378
133	6	0	3.107544	-2.157665	2.238499
134	1	0	2.022330	-3.641934	1.166202
135	1	0	2.590341	-5.602222	2.607229
136	1	0	4.043161	-4.755672	2.040950
137	1	0	3.482321	-4.540902	3.706918
138	1	0	2.519079	-1.319991	1.856137
139	1	0	3.440351	-1.910825	3.253562
140	1	0	4.001381	-2.257229	1.611550
141	6	0	-2.799568	-4.027770	2.702586
142	6	0	-3.910524	-3.043548	3.111628

143	6	0	-3.175759	-5.469619	3.104916
144	1	0	-2.731760	-3.992881	1.611658
145	1	0	-3.669805	-2.019767	2.812944
146	1	0	-4.849195	-3.324276	2.620570
147	1	0	-4.091393	-3.056775	4.192941
148	1	0	-2.426727	-6.196467	2.769604
149	1	0	-3.265589	-5.564630	4.193653
150	1	0	-4.138918	-5.749445	2.662515

----

full(5)

Standard orientation:

Center Number	Atomic Number	Atomic Type	Coordinates (Angstroms) X	Y	Z
1	5	0	1.463651	0.299169	-0.581540
2	5	0	1.030362	-1.316348	-1.227275
3	5	0	-1.365162	-0.463411	-2.424441
4	5	0	-0.667299	-1.658107	-1.320139
5	5	0	-1.486694	-0.198416	-0.642133
6	5	0	-0.044585	0.637469	-2.885815
7	5	0	0.194340	-1.121710	-2.782021
8	6	0	-1.188370	1.094443	-1.718286
9	1	0	0.080897	-1.294105	1.151044
10	1	0	0.967311	1.345603	-0.244685
11	1	0	1.865733	-2.150000	-1.131520
12	1	0	-2.350998	-0.588108	-3.085937
13	1	0	-1.081748	-2.769331	-1.251153
14	1	0	2.194935	0.075282	-2.834731
15	1	0	-0.523362	1.716419	-1.114882
16	1	0	-0.032362	1.203102	-3.941076
17	1	0	0.456966	-1.816945	-3.715355
18	1	0	-2.009545	1.694604	-2.097364
19	6	0	-2.922498	-0.327997	0.013892
20	7	0	-3.467853	-1.397617	0.698310
21	6	0	-4.755528	-1.104959	1.133466
22	1	0	-5.337778	-1.829142	1.678340
23	7	0	-3.926829	0.620993	0.045945
24	6	0	-5.041374	0.149104	0.731579
25	1	0	-5.918931	0.760798	0.861546
26	6	0	2.982828	0.367141	-0.038245
27	7	0	3.470466	1.489268	0.594086
28	6	0	4.822561	1.365879	0.879508
29	1	0	5.368138	2.152480	1.374831
30	7	0	4.089224	-0.451992	-0.136658
31	6	0	5.207564	0.155316	0.424772
32	1	0	6.162052	-0.344661	0.443280
33	6	0	1.297462	0.057999	-2.222442
34	5	0	-0.005824	-0.779832	0.071821
35	6	0	-3.964404	1.968019	-0.486605
36	6	0	-4.586569	2.186482	-1.733724
37	6	0	-3.502032	3.021943	0.323778
38	6	0	-4.712784	3.511698	-2.166911

39	6	0	-3.650276	4.326696	-0.162633
40	6	0	-4.247096	4.571963	-1.394863
41	1	0	-5.181715	3.715026	-3.124755
42	1	0	-3.299838	5.161312	0.437078
43	1	0	-4.353895	5.592350	-1.753615
44	6	0	-2.943762	-2.729543	0.945252
45	6	0	-3.224538	-3.743496	0.005801
46	6	0	-2.316745	-2.988974	2.179875
47	6	0	-2.819405	-5.044504	0.323403
48	6	0	-1.930277	-4.309609	2.440714
49	6	0	-2.172941	-5.326022	1.522687
50	1	0	-3.010583	-5.847879	-0.380841
51	1	0	-1.438157	-4.546198	3.378560
52	1	0	-1.863431	-6.343816	1.746124
53	6	0	2.781565	2.709519	0.958099
54	6	0	2.671804	3.740963	0.003833
55	6	0	2.390950	2.871881	2.301017
56	6	0	2.142678	4.962365	0.435919
57	6	0	1.877300	4.118827	2.679204
58	6	0	1.754647	5.155195	1.759306
59	1	0	2.044855	5.778383	-0.273945
60	1	0	1.571816	4.277880	3.709543
61	1	0	1.356860	6.116570	2.073737
62	6	0	4.261160	-1.790762	-0.677874
63	6	0	4.743613	-1.930223	-1.993609
64	6	0	4.086048	-2.888489	0.187256
65	6	0	5.029966	-3.226302	-2.440160
66	6	0	4.393317	-4.158495	-0.311871
67	6	0	4.857440	-4.330087	-1.612271
68	1	0	5.396400	-3.370554	-3.451839
69	1	0	4.263503	-5.026316	0.327766
70	1	0	5.086305	-5.326545	-1.980779
71	6	0	-3.990617	-3.477985	-1.288932
72	6	0	-5.480924	-3.845427	-1.120683
73	6	0	-3.399527	-4.207505	-2.508568
74	1	0	-3.928026	-2.407158	-1.503301
75	1	0	-5.953368	-3.284791	-0.306838
76	1	0	-6.033752	-3.629118	-2.042723
77	1	0	-5.597031	-4.913938	-0.902086
78	1	0	-2.339242	-3.973896	-2.631262
79	1	0	-3.513628	-5.295537	-2.432428
80	1	0	-3.926254	-3.889739	-3.415717
81	6	0	-2.123479	-1.910487	3.244110
82	6	0	-0.774321	-2.012707	3.974864
83	6	0	-3.275193	-1.943012	4.272532
84	1	0	-2.141675	-0.938460	2.741236
85	1	0	0.056562	-2.008940	3.265137
86	1	0	-0.655774	-1.156629	4.648736
87	1	0	-0.707187	-2.918083	4.589780
88	1	0	-4.252123	-1.788632	3.803687
89	1	0	-3.303079	-2.907428	4.794034
90	1	0	-3.137988	-1.157103	5.025178
91	6	0	-2.904715	2.787516	1.706712

92	6	0	-3.919825	3.155857	2.808501
93	6	0	-1.579945	3.539395	1.907373
94	1	0	-2.681446	1.721224	1.804155
95	1	0	-4.845830	2.577721	2.713747
96	1	0	-3.497458	2.956276	3.800575
97	1	0	-4.183738	4.219372	2.762533
98	1	0	-0.850516	3.273696	1.137109
99	1	0	-1.715983	4.627123	1.887254
100	1	0	-1.149817	3.282943	2.881082
101	6	0	-5.136776	1.051305	-2.593757
102	6	0	-4.689699	1.151305	-4.063893
103	6	0	-6.675679	0.988440	-2.499459
104	1	0	-4.739143	0.109694	-2.204039
105	1	0	-3.600322	1.193038	-4.147491
106	1	0	-5.034622	0.269378	-4.615554
107	1	0	-5.110641	2.033027	-4.561255
108	1	0	-7.015148	0.847314	-1.467297
109	1	0	-7.131463	1.911552	-2.877470
110	1	0	-7.060802	0.154121	-3.097401
111	6	0	4.994538	-0.742579	-2.919232
112	6	0	4.397491	-0.953011	-4.323800
113	6	0	6.501909	-0.426952	-3.016255
114	1	0	4.502054	0.133795	-2.486496
115	1	0	3.338550	-1.222718	-4.274281
116	1	0	4.489182	-0.031054	-4.909528
117	1	0	4.923361	-1.741451	-4.874612
118	1	0	6.934049	-0.200134	-2.035225
119	1	0	7.054461	-1.275872	-3.436253
120	1	0	6.670617	0.439267	-3.667026
121	6	0	3.593933	-2.740414	1.623388
122	6	0	4.740541	-2.985492	2.625524
123	6	0	2.392692	-3.656112	1.921220
124	1	0	3.247498	-1.711740	1.760341
125	1	0	5.576979	-2.296609	2.459621
126	1	0	4.386868	-2.846370	3.654153
127	1	0	5.129211	-4.007389	2.540455
128	1	0	1.565760	-3.454608	1.234743
129	1	0	2.659095	-4.716857	1.844420
130	1	0	2.038385	-3.481758	2.943843
131	6	0	3.144071	3.584811	-1.438064
132	6	0	4.433247	4.396825	-1.679621
133	6	0	2.052372	3.960130	-2.456207
134	1	0	3.380443	2.530679	-1.605498
135	1	0	5.234062	4.096441	-0.993871
136	1	0	4.791433	4.247295	-2.704901
137	1	0	4.259284	5.470395	-1.538917
138	1	0	1.156902	3.348271	-2.318695
139	1	0	1.769728	5.017090	-2.381809
140	1	0	2.418946	3.788494	-3.474532
141	6	0	2.519441	1.760153	3.338285
142	6	0	1.148176	1.362063	3.914244
143	6	0	3.503486	2.144034	4.460791
144	1	0	2.921908	0.873308	2.840432

145	1	0	0.472324	1.032919	3.118972
146	1	0	1.265092	0.536873	4.626219
147	1	0	0.676949	2.196425	4.447647
148	1	0	4.495551	2.385080	4.061900
149	1	0	3.151279	3.016687	5.023287
150	1	0	3.616095	1.314944	5.169140

----

full(p-2)

Standard orientation:

Center Number	Atomic Number	Atomic Type	Coordinates (Angstroms) X	Y	Z
1	6	0	-0.987660	-1.184454	-1.181272
2	5	0	-1.544596	0.099397	-0.297419
3	5	0	-1.381334	0.247990	-2.075086
4	5	0	0.591468	-1.493680	-1.147732
5	5	0	-0.128661	-0.877716	-2.646539
6	5	0	-0.591287	1.493504	-1.148463
7	5	0	1.544658	-0.099399	-0.297384
8	5	0	0.128723	0.876833	-2.647002
9	5	0	1.381395	-0.248535	-2.075018
10	6	0	0.987792	1.184185	-1.181912
11	1	0	-1.625035	-2.053483	-1.306860
12	1	0	-0.956937	0.264610	0.745371
13	1	0	-2.426471	0.352069	-2.642992
14	1	0	0.958751	-2.627652	-1.090716
15	1	0	-0.297827	-1.547927	-3.619034
16	1	0	-0.958619	2.627451	-1.091568
17	1	0	0.957110	-0.263531	0.745651
18	1	0	0.297900	1.546532	-3.619850
19	1	0	2.426574	-0.352869	-2.642823
20	1	0	1.625228	2.053133	-1.307756
21	6	0	3.125152	-0.204007	0.017396
22	7	0	4.113416	0.741501	-0.106310
23	6	0	5.322685	0.265310	0.391717
24	1	0	6.210047	0.877009	0.387008
25	7	0	3.761018	-1.273907	0.593723
26	6	0	5.101665	-0.992432	0.830092
27	1	0	5.757229	-1.718178	1.282935
28	6	0	-3.125047	0.203997	0.017410
29	7	0	-4.113430	-0.741384	-0.106271
30	6	0	-5.322617	-0.265075	0.391876
31	1	0	-6.210034	-0.876692	0.387258
32	7	0	-3.760767	1.273944	0.593866
33	6	0	-5.101412	0.992611	0.830320
34	1	0	-5.756852	1.718377	1.283315
35	6	0	3.210966	-2.570365	0.933420
36	6	0	3.342937	-3.621746	0.003657
37	6	0	2.669766	-2.752741	2.219630
38	6	0	2.886821	-4.884783	0.396742
39	6	0	2.224309	-4.037089	2.555928
40	6	0	2.327624	-5.091692	1.654399

41	1	0	2.967775	-5.717658	-0.294643
42	1	0	1.800828	-4.213925	3.540189
43	1	0	1.977569	-6.081636	1.935492
44	6	0	-3.210513	2.570314	0.933535
45	6	0	-2.669232	2.752558	2.219722
46	6	0	-3.342348	3.621743	0.003805
47	6	0	-2.223403	4.036781	2.556004
48	6	0	-2.885928	4.884675	0.396901
49	6	0	-2.326530	5.091420	1.654497
50	1	0	-1.799773	4.213468	3.540225
51	1	0	-2.966815	5.717613	-0.294413
52	1	0	-1.976207	6.081277	1.935562
53	6	0	4.028563	2.106268	-0.589544
54	6	0	4.465813	2.388378	-1.899969
55	6	0	3.615804	3.112396	0.306216
56	6	0	4.465319	3.729309	-2.303323
57	6	0	3.632216	4.433878	-0.154856
58	6	0	4.051390	4.742606	-1.444864
59	1	0	4.791711	3.980793	-3.307750
60	1	0	3.312195	5.231424	0.509205
61	1	0	4.055345	5.775651	-1.782399
62	6	0	-4.028828	-2.106157	-0.589534
63	6	0	-4.466388	-2.388228	-1.899857
64	6	0	-3.616037	-3.112319	0.306182
65	6	0	-4.466195	-3.729179	-2.303157
66	6	0	-3.632770	-4.433816	-0.154830
67	6	0	-4.052264	-4.742513	-1.444746
68	1	0	-4.792838	-3.980655	-3.307506
69	1	0	-3.312742	-5.231389	0.509194
70	1	0	-4.056466	-5.775566	-1.782253
71	6	0	2.619343	-1.632904	3.254669
72	6	0	3.705402	-1.837810	4.331050
73	6	0	1.230915	-1.482745	3.898433
74	1	0	2.833932	-0.689435	2.744484
75	1	0	4.707165	-1.884635	3.889034
76	1	0	3.692378	-1.011807	5.052209
77	1	0	3.542374	-2.770373	4.884387
78	1	0	0.465231	-1.310684	3.136765
79	1	0	0.949713	-2.370211	4.477706
80	1	0	1.228561	-0.629007	4.586018
81	6	0	4.005959	-3.436320	-1.359522
82	6	0	3.227946	-4.100833	-2.509345
83	6	0	5.460884	-3.951746	-1.326044
84	1	0	4.033776	-2.364511	-1.577259
85	1	0	2.197588	-3.738983	-2.544760
86	1	0	3.709264	-3.860007	-3.464233
87	1	0	3.214894	-5.193722	-2.419092
88	1	0	6.058785	-3.445308	-0.560027
89	1	0	5.490314	-5.027365	-1.113786
90	1	0	5.946408	-3.787016	-2.295212
91	6	0	3.172751	2.822596	1.736334
92	6	0	1.753516	3.352144	2.012108
93	6	0	4.187282	3.382618	2.753533

94	1	0	3.139324	1.737888	1.871663
95	1	0	1.030957	2.930968	1.307009
96	1	0	1.444140	3.079665	3.027726
97	1	0	1.707100	4.444977	1.936392
98	1	0	5.189274	2.968603	2.591693
99	1	0	4.262640	4.474201	2.682852
100	1	0	3.879026	3.134780	3.776242
101	6	0	4.955324	1.311297	-2.864243
102	6	0	6.490523	1.359889	-3.010031
103	6	0	4.274397	1.405855	-4.242352
104	1	0	4.689746	0.336641	-2.445359
105	1	0	6.996449	1.228420	-2.046567
106	1	0	6.835132	0.565423	-3.682407
107	1	0	6.817735	2.319173	-3.428700
108	1	0	3.185544	1.376020	-4.148472
109	1	0	4.552553	2.323910	-4.773334
110	1	0	4.584510	0.560233	-4.867075
111	6	0	-2.619199	1.632715	3.254783
112	6	0	-3.705362	1.837912	4.331002
113	6	0	-1.230918	1.482189	3.898790
114	1	0	-2.833962	0.689294	2.744575
115	1	0	-4.707097	1.884463	3.888895
116	1	0	-3.692322	1.012185	5.052480
117	1	0	-3.542469	2.770703	4.883988
118	1	0	-0.465073	1.310417	3.137229
119	1	0	-0.949807	2.369372	4.478545
120	1	0	-1.228777	0.628134	4.585981
121	6	0	-4.005635	3.436538	-1.359279
122	6	0	-3.227748	4.101076	-2.509185
123	6	0	-5.460505	3.952095	-1.325504
124	1	0	-4.033651	2.364751	-1.577112
125	1	0	-2.197457	3.739072	-2.544862
126	1	0	-3.709307	3.860497	-3.464013
127	1	0	-3.214493	5.193945	-2.418748
128	1	0	-6.058185	3.445933	-0.559129
129	1	0	-5.489812	5.027784	-1.113575
130	1	0	-5.946329	3.787090	-2.294473
131	6	0	-3.172706	-2.822504	1.736207
132	6	0	-1.753732	-3.352734	2.012035
133	6	0	-4.187503	-3.381810	2.753540
134	1	0	-3.138658	-1.737792	1.871332
135	1	0	-1.031001	-2.932106	1.306784
136	1	0	-1.444153	-3.080099	3.027551
137	1	0	-1.707867	-4.445613	1.936658
138	1	0	-5.189295	-2.967339	2.591624
139	1	0	-4.263392	-4.473371	2.683052
140	1	0	-3.879124	-3.133927	3.776202
141	6	0	-4.955957	-1.311104	-2.864048
142	6	0	-6.491204	-1.359443	-3.009459
143	6	0	-4.275417	-1.405848	-4.242335
144	1	0	-4.690102	-0.336476	-2.445274
145	1	0	-6.996883	-1.227950	-2.045870
146	1	0	-6.835855	-0.564894	-3.681714

147	1	0	-6.818653	-2.318658	-3.428100
148	1	0	-3.186534	-1.376211	-4.148747
149	1	0	-4.553882	-2.323879	-4.773197
150	1	0	-4.585550	-0.560202	-4.867017

----

full(o-2a)

Standard orientation:

Center Number	Atomic Number	Atomic Type	Coordinates (Angstroms) X	Y	Z
1	5	0	-1.559644	0.208056	-0.374338
2	5	0	-1.395012	-0.328519	-2.061570
3	5	0	0.998577	1.213276	-1.893807
4	5	0	0.032765	0.186706	-2.969882
5	5	0	1.616316	0.049843	-0.367881
6	5	0	-0.047981	-1.436323	-2.232466
7	5	0	1.403239	-0.464067	-2.058267
8	6	0	0.725923	-1.291922	-0.712639
9	1	0	-1.420894	2.248001	-2.291018
10	1	0	-0.915860	0.849201	0.398636
11	1	0	-2.427831	-0.603487	-2.565480
12	1	0	1.658214	2.114313	-2.287107
13	1	0	0.036434	0.274124	-4.155002
14	1	0	-1.305005	-2.148480	-0.465422
15	1	0	1.043512	0.750095	0.409606
16	1	0	-0.097829	-2.478385	-2.793898
17	1	0	2.406559	-0.848827	-2.562500
18	1	0	1.115082	-2.268758	-0.453244
19	6	0	3.170710	-0.092476	0.066508
20	7	0	3.830369	-1.193356	0.534057
21	6	0	5.141608	-0.874833	0.862036
22	1	0	5.817949	-1.616343	1.255129
23	7	0	4.091246	0.917582	0.121589
24	6	0	5.304687	0.441002	0.597298
25	1	0	6.161218	1.087276	0.694712
26	6	0	-3.099296	0.250089	0.099189
27	7	0	-4.082743	-0.700117	0.087213
28	6	0	-5.216599	-0.227158	0.734591
29	1	0	-6.099610	-0.836438	0.834908
30	7	0	-3.651240	1.320976	0.748160
31	6	0	-4.948087	1.035369	1.141498
32	1	0	-5.550033	1.755966	1.669666
33	6	0	-0.812218	-1.217087	-0.718577
34	5	0	-0.835482	1.298184	-1.895482
35	6	0	-4.084304	-2.029393	-0.512447
36	6	0	-3.644005	-3.123992	0.262507
37	6	0	-4.643084	-2.178705	-1.800403
38	6	0	-3.726112	-4.395041	-0.319018
39	6	0	-4.699203	-3.476134	-2.324684
40	6	0	-4.240078	-4.570691	-1.600005
41	1	0	-3.394011	-5.260979	0.244013
42	1	0	-5.114922	-3.630532	-3.314737

43	1	0	-4.294996	-5.566719	-2.030071
44	6	0	3.321513	-2.536326	0.757722
45	6	0	3.524903	-3.511599	-0.241828
46	6	0	2.730889	-2.826056	2.003742
47	6	0	3.060553	-4.805234	0.027302
48	6	0	2.283020	-4.136771	2.211378
49	6	0	2.437866	-5.114572	1.233398
50	1	0	3.195686	-5.583141	-0.717127
51	1	0	1.821073	-4.396680	3.159032
52	1	0	2.087536	-6.126264	1.417669
53	6	0	-3.048725	2.629563	0.957453
54	6	0	-2.397730	2.912290	2.176537
55	6	0	-3.209034	3.595261	-0.066115
56	6	0	-1.816566	4.184402	2.300932
57	6	0	-2.615678	4.846903	0.130500
58	6	0	-1.909245	5.135890	1.293387
59	1	0	-1.297725	4.429936	3.223934
60	1	0	-2.709913	5.606815	-0.636883
61	1	0	-1.448160	6.111212	1.421393
62	6	0	3.976521	2.277521	-0.392187
63	6	0	4.359907	2.483616	-1.736446
64	6	0	3.599248	3.335946	0.465743
65	6	0	4.289643	3.788205	-2.238732
66	6	0	3.533149	4.613262	-0.110053
67	6	0	3.861307	4.842097	-1.441956
68	1	0	4.574394	3.977354	-3.268382
69	1	0	3.235673	5.450442	0.515207
70	1	0	3.803490	5.846791	-1.850923
71	6	0	-4.071457	3.353991	-1.306941
72	6	0	-3.597613	4.119232	-2.556207
73	6	0	-5.543942	3.729096	-1.020073
74	1	0	-4.034493	2.285733	-1.547630
75	1	0	-2.532916	3.971098	-2.753051
76	1	0	-4.155156	3.768475	-3.431104
77	1	0	-3.785497	5.195280	-2.468758
78	1	0	-5.970907	3.155013	-0.192721
79	1	0	-5.626904	4.792206	-0.767162
80	1	0	-6.160382	3.545429	-1.907330
81	6	0	4.905530	1.371629	-2.632771
82	6	0	6.445169	1.459870	-2.717387
83	6	0	4.295021	1.375123	-4.046787
84	1	0	4.654048	0.406759	-2.181732
85	1	0	6.916340	1.395697	-1.730459
86	1	0	6.839901	0.644103	-3.333546
87	1	0	6.757897	2.406267	-3.172885
88	1	0	3.202893	1.342087	-4.016625
89	1	0	4.594815	2.259507	-4.619560
90	1	0	4.647632	0.498298	-4.600977
91	6	0	-2.352593	2.043960	3.441935
92	6	0	-2.177736	0.525552	3.292587
93	6	0	-3.568022	2.358380	4.343257
94	1	0	-1.466422	2.396846	3.984654
95	1	0	-1.375461	0.272266	2.594835

96	1	0	-1.920447	0.102357	4.270307
97	1	0	-3.097788	0.032497	2.965859
98	1	0	-3.662591	3.432752	4.529966
99	1	0	-4.502868	2.011257	3.888230
100	1	0	-3.463524	1.851794	5.309561
101	6	0	4.262176	-3.225665	-1.547996
102	6	0	3.527457	-3.776973	-2.783934
103	6	0	5.702977	-3.776708	-1.485634
104	1	0	4.328243	-2.140016	-1.671724
105	1	0	2.498099	-3.410549	-2.840260
106	1	0	4.048250	-3.461366	-3.694411
107	1	0	3.501480	-4.872247	-2.789376
108	1	0	6.267884	-3.351426	-0.648660
109	1	0	5.702356	-4.866113	-1.366291
110	1	0	6.240689	-3.540111	-2.410486
111	6	0	2.608437	-1.801239	3.127209
112	6	0	3.516400	-2.177454	4.316001
113	6	0	1.150748	-1.612238	3.583635
114	1	0	2.954435	-0.833994	2.750974
115	1	0	4.563830	-2.270159	4.008551
116	1	0	3.458652	-1.410097	5.096107
117	1	0	3.215623	-3.131795	4.762573
118	1	0	0.507364	-1.325586	2.745786
119	1	0	0.743977	-2.528372	4.026632
120	1	0	1.093540	-0.825211	4.343976
121	6	0	-5.239554	-1.017807	-2.595418
122	6	0	-6.776608	-0.992530	-2.443685
123	6	0	-4.862727	-1.045555	-4.088797
124	1	0	-4.847730	-0.082844	-2.180700
125	1	0	-7.088178	-0.903413	-1.397680
126	1	0	-7.198392	-0.143670	-2.993465
127	1	0	-7.222831	-1.908890	-2.846275
128	1	0	-3.781213	-1.113589	-4.235448
129	1	0	-5.332182	-1.885459	-4.612450
130	1	0	-5.214438	-0.128178	-4.573211
131	6	0	-3.166446	-2.975613	1.705491
132	6	0	-1.950224	-3.856666	2.043723
133	6	0	-4.321950	-3.270321	2.686747
134	1	0	-2.860295	-1.936620	1.857678
135	1	0	-1.117280	-3.694209	1.351670
136	1	0	-1.598211	-3.622158	3.054218
137	1	0	-2.198441	-4.923489	2.028629
138	1	0	-5.179155	-2.610030	2.517885
139	1	0	-4.671255	-4.303436	2.578546
140	1	0	-3.989737	-3.132268	3.721998
141	6	0	3.343414	3.266206	1.978520
142	6	0	4.608133	2.922419	2.792843
143	6	0	2.146779	2.412338	2.427005
144	1	0	3.088853	4.297205	2.252424
145	1	0	5.464542	3.527124	2.475114
146	1	0	4.429895	3.127559	3.854601
147	1	0	4.881212	1.866095	2.708216
148	1	0	1.234679	2.683229	1.886056

149	1	0	2.319969	1.343038	2.283088
150	1	0	1.968070	2.576027	3.496642
151	1	0	0.103072	1.746681	-1.101293

----

full(m-2a)

Standard orientation:

Center Number	Atomic Number	Atomic Type	Coordinates (Angstroms) X	Y	Z
1	6	0	1.348646	-0.797730	1.278104
2	5	0	0.450766	-1.500522	0.000000
3	5	0	2.704708	0.154139	0.871120
4	5	0	1.102209	0.787671	1.411780
5	5	0	1.102209	0.787671	-1.411780
6	1	0	1.444488	-1.334284	2.213362
7	1	0	2.940786	-2.285566	0.000000
8	1	0	-0.683114	-1.072257	0.000000
9	1	0	3.641465	0.233945	1.595592
10	1	0	1.017661	1.310020	2.479508
11	1	0	1.444488	-1.334284	-2.213362
12	1	0	3.641465	0.233945	-1.595592
13	1	0	-0.261057	0.999447	0.898548
14	1	0	2.614135	2.543255	-0.000000
15	1	0	1.017661	1.310020	-2.479508
16	6	0	0.364676	-3.152822	0.000000
17	7	0	0.288738	-3.984477	-1.080826
18	7	0	0.288738	-3.984477	1.080826
19	6	0	0.171506	-5.307569	0.675849
20	6	0	0.171506	-5.307569	-0.675849
21	1	0	0.094459	-6.109023	1.392464
22	1	0	0.094459	-6.109023	-1.392464
23	5	0	2.314086	-1.279235	0.000000
24	5	0	0.275546	1.692783	-0.000000
25	5	0	2.006770	1.530780	-0.000000
26	6	0	1.348646	-0.797730	-1.278104
27	5	0	2.704708	0.154139	-0.871120
28	6	0	-0.342929	3.180091	-0.000000
29	7	0	-1.686166	3.422474	-0.000000
30	6	0	-1.940393	4.779588	-0.000000
31	1	0	-2.946431	5.166590	-0.000000
32	7	0	0.237279	4.416538	-0.000000
33	6	0	-0.735692	5.400324	-0.000000
34	1	0	-0.470461	6.444964	-0.000000
35	6	0	0.226604	-3.640377	-2.491734
36	6	0	1.406266	-3.712008	-3.261892
37	6	0	-1.035954	-3.351714	-3.045717
38	6	0	1.295252	-3.411442	-4.624871
39	6	0	-1.081562	-3.053262	-4.413395
40	6	0	0.070360	-3.071813	-5.193240
41	1	0	2.180832	-3.449535	-5.251075
42	1	0	-2.036325	-2.821606	-4.875832
43	1	0	0.011567	-2.839885	-6.252916

44	6	0	0.226604	-3.640377	2.491734
45	6	0	1.406266	-3.712008	3.261892
46	6	0	-1.035954	-3.351714	3.045717
47	6	0	1.295252	-3.411442	4.624871
48	6	0	-1.081562	-3.053262	4.413395
49	6	0	0.070360	-3.071813	5.193240
50	1	0	2.180832	-3.449535	5.251075
51	1	0	-2.036325	-2.821606	4.875832
52	1	0	0.011567	-2.839885	6.252916
53	6	0	1.638554	4.825630	-0.000000
54	6	0	2.257769	5.072532	1.239847
55	6	0	2.257769	5.072532	-1.239847
56	6	0	3.566411	5.567919	1.206288
57	6	0	3.566411	5.567919	-1.206288
58	6	0	4.215970	5.810926	-0.000000
59	1	0	4.082680	5.768306	2.139667
60	1	0	4.082680	5.768306	-2.139667
61	1	0	5.231792	6.195643	-0.000000
62	6	0	-2.767337	2.454932	-0.000000
63	6	0	-3.303275	2.058384	1.241499
64	6	0	-3.303275	2.058384	-1.241499
65	6	0	-4.405752	1.195896	1.208073
66	6	0	-4.405752	1.195896	-1.208073
67	6	0	-4.948080	0.765830	-0.000000
68	1	0	-4.855165	0.870734	2.141122
69	1	0	-4.855165	0.870734	-2.141122
70	1	0	-5.810756	0.105393	-0.000000
71	6	0	-2.785381	2.589487	2.576848
72	6	0	-2.461221	1.470925	3.583390
73	6	0	-3.787965	3.594578	3.182613
74	1	0	-1.851801	3.131621	2.393540
75	1	0	-1.721367	0.769687	3.185052
76	1	0	-2.051958	1.906338	4.501581
77	1	0	-3.355066	0.901570	3.860960
78	1	0	-3.991933	4.426459	2.499281
79	1	0	-4.744423	3.110687	3.410366
80	1	0	-3.392205	4.011812	4.115158
81	6	0	-2.785381	2.589487	-2.576848
82	6	0	-3.787965	3.594578	-3.182613
83	6	0	-2.461221	1.470925	-3.583390
84	1	0	-1.851801	3.131621	-2.393540
85	1	0	-3.991933	4.426459	-2.499281
86	1	0	-3.392205	4.011812	-4.115158
87	1	0	-4.744423	3.110687	-3.410366
88	1	0	-1.721367	0.769687	-3.185052
89	1	0	-3.355066	0.901570	-3.860960
90	1	0	-2.051958	1.906338	-4.501581
91	6	0	1.564831	4.851798	2.581894
92	6	0	1.160908	6.197795	3.219177
93	6	0	2.423696	4.022874	3.555287
94	1	0	0.645734	4.282015	2.405396
95	1	0	0.497865	6.776080	2.565603
96	1	0	0.638609	6.030370	4.167979

97	1	0	2.042464	6.814881	3.426186
98	1	0	2.725889	3.071061	3.109245
99	1	0	3.328353	4.560922	3.858757
100	1	0	1.852080	3.808605	4.465315
101	6	0	1.564831	4.851798	-2.581894
102	6	0	2.423696	4.022874	-3.555287
103	6	0	1.160908	6.197795	-3.219177
104	1	0	0.645734	4.282015	-2.405396
105	1	0	2.725889	3.071061	-3.109245
106	1	0	1.852080	3.808605	-4.465315
107	1	0	3.328353	4.560922	-3.858757
108	1	0	0.497865	6.776080	-2.565603
109	1	0	2.042464	6.814881	-3.426186
110	1	0	0.638609	6.030370	-4.167979
111	6	0	2.750483	-4.150065	-2.684182
112	6	0	3.061675	-5.605916	-3.093291
113	6	0	3.911230	-3.220284	-3.083651
114	1	0	2.677922	-4.121585	-1.592452
115	1	0	2.277038	-6.298891	-2.769360
116	1	0	4.006719	-5.933141	-2.645665
117	1	0	3.155414	-5.697197	-4.181387
118	1	0	3.716112	-2.181339	-2.801839
119	1	0	4.106971	-3.250531	-4.161217
120	1	0	4.829490	-3.538704	-2.578407
121	6	0	-2.333262	-3.433278	-2.245770
122	6	0	-3.152299	-2.133971	-2.311846
123	6	0	-3.175617	-4.641863	-2.704774
124	1	0	-2.080789	-3.595718	-1.193624
125	1	0	-2.576512	-1.283203	-1.936810
126	1	0	-4.054952	-2.228412	-1.697480
127	1	0	-3.472242	-1.907223	-3.335237
128	1	0	-2.615790	-5.579609	-2.617867
129	1	0	-3.486096	-4.536760	-3.750372
130	1	0	-4.081064	-4.729583	-2.093516
131	6	0	-2.333262	-3.433278	2.245770
132	6	0	-3.175617	-4.641863	2.704774
133	6	0	-3.152299	-2.133971	2.311846
134	1	0	-2.080789	-3.595718	1.193624
135	1	0	-2.615790	-5.579609	2.617867
136	1	0	-4.081064	-4.729583	2.093516
137	1	0	-3.486096	-4.536760	3.750372
138	1	0	-2.576512	-1.283203	1.936810
139	1	0	-3.472242	-1.907223	3.335237
140	1	0	-4.054952	-2.228412	1.697480
141	6	0	2.750483	-4.150065	2.684182
142	6	0	3.911230	-3.220284	3.083651
143	6	0	3.061675	-5.605916	3.093291
144	1	0	2.677922	-4.121585	1.592452
145	1	0	3.716112	-2.181339	2.801839
146	1	0	4.829490	-3.538704	2.578407
147	1	0	4.106971	-3.250531	4.161217
148	1	0	2.277038	-6.298891	2.769360
149	1	0	3.155414	-5.697197	4.181387

150	1	0	4.006719	-5.933141	2.645665
151	1	0	-0.261057	0.999447	-0.898548

full(p-2a)

Standard orientation:

Center Number	Atomic Number	Atomic Type	Coordinates (Angstroms) X	Y	Z
1	6	0	-0.904306	-1.185344	-1.299537
2	5	0	-1.624520	-0.055906	-0.364710
3	5	0	-1.475919	0.246415	-2.094784
4	5	0	0.725290	-1.314544	-1.196388
5	5	0	-0.053311	-0.678462	-2.669402
6	5	0	-0.836293	1.530206	-1.110813
7	5	0	1.536214	0.223453	-0.185488
8	5	0	-0.028549	1.081753	-2.663958
9	5	0	1.349608	0.181589	-2.119913
10	6	0	0.736860	1.420749	-1.201499
11	1	0	-1.438162	-2.103813	-1.511743
12	1	0	-1.003844	0.272178	0.615755
13	1	0	-2.497484	0.184952	-2.694020
14	1	0	1.263431	-2.364726	-1.316375
15	1	0	-0.018581	-1.390800	-3.618621
16	1	0	-1.344876	2.587207	-0.927166
17	1	0	1.068215	0.551098	0.881574
18	1	0	0.075650	1.778361	-3.619397
19	1	0	2.402412	0.258264	-2.660336
20	1	0	1.299004	2.346513	-1.143097
21	6	0	3.158438	0.233606	-0.012435
22	7	0	4.079777	-0.767550	-0.083609
23	6	0	5.338547	-0.284127	0.245994
24	1	0	6.200813	-0.930522	0.251659
25	7	0	3.859777	1.341487	0.361614
26	6	0	5.202100	1.034062	0.519526
27	1	0	5.919735	1.783059	0.812134
28	6	0	-3.190450	-0.248065	0.006763
29	7	0	-4.196501	0.673175	0.017101
30	6	0	-5.371031	0.102804	0.488327
31	1	0	-6.277669	0.678801	0.575890
32	7	0	-3.760833	-1.397485	0.471261
33	6	0	-5.100720	-1.192769	0.769526
34	1	0	-5.719248	-1.985123	1.158384
35	1	0	1.022670	-0.976127	0.025726
36	6	0	3.355055	2.681808	0.616859
37	6	0	3.454281	3.642900	-0.411740
38	6	0	2.876006	2.979249	1.908258
39	6	0	3.005133	4.935934	-0.117136
40	6	0	2.438454	4.289446	2.138589
41	6	0	2.494969	5.254851	1.138140
42	1	0	3.061243	5.705014	-0.880594
43	1	0	2.061947	4.559758	3.120109
44	1	0	2.152668	6.265567	1.341382

45	6	0	3.895922	-2.178129	-0.397523
46	6	0	4.211768	-2.622517	-1.696895
47	6	0	3.517472	-3.048724	0.644817
48	6	0	4.093727	-3.995809	-1.944244
49	6	0	3.415395	-4.408994	0.333256
50	6	0	3.693485	-4.878481	-0.947099
51	1	0	4.323562	-4.377884	-2.933583
52	1	0	3.124817	-5.112802	1.106079
53	1	0	3.609653	-5.939505	-1.164696
54	6	0	-3.107935	-2.662786	0.756648
55	6	0	-3.171216	-3.693035	-0.205029
56	6	0	-2.522776	-2.830809	2.026890
57	6	0	-2.573295	-4.913547	0.130050
58	6	0	-1.937647	-4.073570	2.302075
59	6	0	-1.955333	-5.100795	1.363739
60	1	0	-2.597126	-5.731593	-0.582424
61	1	0	-1.479566	-4.242790	3.271982
62	1	0	-1.500481	-6.058656	1.600216
63	6	0	-4.152661	2.083169	-0.340827
64	6	0	-3.754960	3.007638	0.645484
65	6	0	-4.613900	2.470422	-1.615390
66	6	0	-3.797647	4.364417	0.305868
67	6	0	-4.628529	3.842194	-1.896700
68	6	0	-4.222098	4.779163	-0.952459
69	1	0	-3.500325	5.106779	1.039845
70	1	0	-4.972225	4.180921	-2.868563
71	1	0	-4.246705	5.837897	-1.194790
72	6	0	4.712426	-1.699280	-2.804839
73	6	0	3.955344	-1.902495	-4.130736
74	6	0	6.230786	-1.877828	-3.018543
75	1	0	4.541087	-0.664258	-2.492705
76	1	0	2.874437	-1.807140	-3.995387
77	1	0	4.275678	-1.149673	-4.859295
78	1	0	4.159505	-2.885522	-4.569177
79	1	0	6.801017	-1.680103	-2.103810
80	1	0	6.465782	-2.898749	-3.340035
81	1	0	6.588840	-1.191265	-3.793819
82	6	0	3.292475	-2.576606	2.079273
83	6	0	4.563760	-2.796439	2.927402
84	6	0	2.079324	-3.245823	2.748541
85	1	0	3.092960	-1.499290	2.061490
86	1	0	5.428296	-2.269234	2.509900
87	1	0	4.409593	-2.434581	3.950413
88	1	0	4.816489	-3.861434	2.980272
89	1	0	1.175783	-3.153968	2.137383
90	1	0	2.253704	-4.311131	2.935594
91	1	0	1.886892	-2.776055	3.719240
92	6	0	4.074772	3.341731	-1.775672
93	6	0	5.514292	3.896207	-1.844467
94	6	0	3.240909	3.877598	-2.954690
95	1	0	4.132661	2.254329	-1.893674
96	1	0	6.150351	3.487754	-1.051383
97	1	0	5.972959	3.645803	-2.807420

98	1	0	5.518585	4.987338	-1.743016
99	1	0	2.214304	3.499585	-2.936602
100	1	0	3.200658	4.972234	-2.963418
101	1	0	3.696691	3.562899	-3.899569
102	6	0	2.892211	1.970591	3.054004
103	6	0	1.546970	1.888339	3.797079
104	6	0	4.040370	2.289781	4.034874
105	1	0	3.087595	0.977686	2.636164
106	1	0	0.725035	1.660841	3.111126
107	1	0	1.588658	1.099255	4.556311
108	1	0	1.308221	2.824309	4.313957
109	1	0	5.013184	2.295225	3.530866
110	1	0	3.901084	3.272827	4.498484
111	1	0	4.077906	1.542447	4.835494
112	6	0	-3.909264	-3.536828	-1.534208
113	6	0	-5.322047	-4.153959	-1.442499
114	6	0	-3.149706	-4.139203	-2.731140
115	1	0	-4.028244	-2.465849	-1.733045
116	1	0	-5.917405	-3.700006	-0.643197
117	1	0	-5.861474	-4.012545	-2.385661
118	1	0	-5.265113	-5.230313	-1.244262
119	1	0	-2.130846	-3.747462	-2.814065
120	1	0	-3.087013	-5.231060	-2.668076
121	1	0	-3.677856	-3.899258	-3.660063
122	6	0	-2.572221	-1.758925	3.113207
123	6	0	-1.178632	-1.403765	3.660482
124	6	0	-3.518308	-2.186716	4.254561
125	1	0	-2.985132	-0.844375	2.677108
126	1	0	-0.512078	-1.067790	2.859642
127	1	0	-1.260805	-0.596732	4.397495
128	1	0	-0.708936	-2.257953	4.160315
129	1	0	-4.527889	-2.395248	3.883795
130	1	0	-3.155029	-3.091394	4.754936
131	1	0	-3.590763	-1.393503	5.007208
132	6	0	-3.342172	2.594818	2.055489
133	6	0	-4.476913	2.882384	3.060723
134	6	0	-2.030148	3.262046	2.506247
135	1	0	-3.166105	1.514424	2.060592
136	1	0	-5.401187	2.361725	2.786547
137	1	0	-4.190430	2.556127	4.067245
138	1	0	-4.700439	3.954312	3.105590
139	1	0	-1.223826	3.081166	1.788583
140	1	0	-2.142619	4.345691	2.622778
141	1	0	-1.724260	2.861594	3.479491
142	6	0	-5.145997	1.482000	-2.650841
143	6	0	-4.599594	1.746782	-4.066219
144	6	0	-6.689975	1.488704	-2.667096
145	1	0	-4.820352	0.477231	-2.362222
146	1	0	-3.506911	1.788104	-4.074956
147	1	0	-4.916488	0.944092	-4.741252
148	1	0	-4.980727	2.686389	-4.481062
149	1	0	-7.113701	1.239186	-1.688070
150	1	0	-7.074182	2.474636	-2.952296

151	1	0	-7.066443	0.758588	-3.392474
